# Construction and curation of a data set of historical mental health incidence in Norway

**DOI:** 10.1038/s41597-025-05795-y

**Published:** 2025-08-28

**Authors:** A. O. Blinkova, U. Khakurel, H. G. Gaddy, S.-E. Mamelund, M. Bekker-Nielsen Dunbar

**Affiliations:** 1 Centre for Research on Pandemics & Society, Oslo Metropolitan University, Oslo, Norway; 2 Department of Economic History, London School of Economics, London, UK

**Keywords:** Psychiatric disorders, Signs and symptoms

## Abstract

We present a structured data set allowing opportunity for insights into mental health admissions to Norwegian facilities covering the period 1872 to 1929. This resource enables quantitative analysis of historical mental health trends across multiple decades and may provide a deeper understanding of the burden of post-viral mental health conditions, which are of renewed interest following the coronavirus disease pandemic of the early twenty-first century. Our data set includes records from 29 facilities, comprising council, private, incarceration, state, and hospital facilities. To construct and curate our data set, we used optical character recognition using ABBYY Finereader to extract tables from historical reports. It was followed by manual validation, harmonization of facility names, and mapping of historical diagnostic terms to Bertillon’s classification of causes. In addition, sex and geography were incorporated as explanatory variables. We believe our data set offers a foundation for comparative historical studies and will contribute additional evidence to understanding long-term mental health patterns.

## Background & Summary

As a result of increased capability and capacity in digital storage of archival records, a wealth of information is now readily available. If we consider the traditional physical archive to be a gold mine, the digital archive is equivalent to panning for gold. The gold ore in the mine is under the surface and has great requirements for extraction in terms of human effort. Panning requires fewer resources, as the digital archive is accessible off site. No travel is required though a good internet connection and certain software needs do exist. While available, the information contained in the digital archive may be under-utilised in research which means gaps in our knowledge can be but are not being filled. While data is available, it is not provided in a format that can be easily used in quantitative research. We describe our approach to extracting this digital data and making it available for analysis.

This project was motivated by a desire to understand the link between physical and mental disease. The impact of an outbreak of infectious disease is not determined solely by mortality but also morbidity reflecting longer-term disability, complications, and sequelae. We are particularly interested in the extended morbidity following viral infections. Known examples are given in Table 1, namely myalgic encephalomyelitis and post-acute coronavirus disease 2019 syndrome. Both are examples of somatic symptom disorders. A somatic symptom disorder relates to the body/physical symptoms and has subjective evidence of disease only perceived by the patient. The symptom is opposed to signs which are objective evidence that can be observed by the medic such as bleeding. The umbrella term for somatic symptom disorders is post-viral mental health conditions. Many have been identified throughout time, including but not limited to febricula (1750), neurasthenia (1869), post-influenza psychosis (1889), and encephalitis lethargica (1920)^1^.

**Table 1:** Three known but not fully determined post-viral health conditions.

Somatic symptom disorder	Suggested causes (infectious)
myalgic encephalomyelitis (colloquially known as “chronic fatigue syndrome”) • Pain in a group of muscles • Relating to the brain • Inflammation of the spinal cord	Pneumonia mononucleosis
Fibromyalgia syndrome • Fibrous (connective) tissues • Pain in a group of muscles • Recognisable complex of symptoms	hepatitis
Post-acute coronavirus disease 2019 syndrome (colloquially known as “long COVID”) • After • Severe and sudden in onset • Recognisable complex of symptoms	Severe acute respiratory syndrome coronavirus 2

Decoration has been added to the text to aid the reader.

Note: pneumonia is a bacterium rather than a virus.

An understanding of historical mental health is therefore important to determine the full impact of historical infectious disease outbreaks. In this work, we consider the annual reported operations at Norwegian mental health institutions; historically these facilities were referred to with several terms that are no longer considered appropriate, e.g., asylum (archaic term for mental health facilities, see supporting information for full names of reports used). As “asylum” is loaded term with certain negative connotations, in the construction of our data and henceforth we instead refer to the various mental healthcare institutions as *facilities* apart from where we need to distinguish them. We are interested in both specific mental health admissions that may help us understand the medically unexplained physical symptoms common in post-viral mental health conditions but include all causes for admissions in this work to allow for greatest reuse. The data created in this work allows us to investigate classification of mental illness found in the facility records at greater granularity than considered previously. Mental health conditions such as depression and anxiety are a leading cause of the global burden of disease and there may be evidence to be found about their occurrence and our understanding of them historically.

**Fig. 1:**
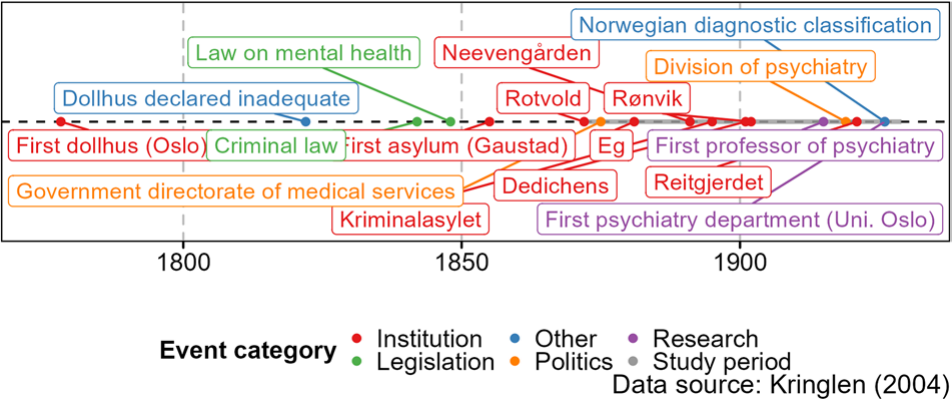
Key events related to Norwegian mental health historically leading up to and including our study period (marked in grey).

Understanding the historical development of mental health care infrastructure and terminology is therefore essential to accurately interpret the records and admission data used in this study. To situate our work in with a broader context, we briefly outline the origins and transformation of mental health facilities in Norway. A push for a Mental Health Act in Norway was led by the male legislator Herman Wedel Major, who was inspired by the *Irrenanstalt* in *Schleswig* (initiated in 1817, open from 1820). He and his niece and partner (“søsterdatter”) Fanny Rahbek were mentally ill so he had personal experience with mental health, which contributed to his personal engagement with mental health care in Norway^2^. In 1848, a Norwegian Mental Health Act was instated (“sinnsjukeloven”), declaring the responsibility of the care of the mentally ill rested with the government. The first mental health facility in Norway (Gaustad) was opened in 1855 following the Act^2^. Before the advent of facilities, treatment was either at home, in mental health colonies (“sinssykekolonier”) or at specific rooms in hospitals. Our work does not consider *dollhus* (“madhouse”); the predecessor to facilities (Fig. 1). Before *dollhus*, there was *dårekiste*, a type of coffin with bars to contain patients. This is reflective of the understanding of treatment at the time which is very different than contemporary understandings. The history of the psychological health landscape in Norway spans multiple decades and underwent significant transformations^3,4^. Placed in their historical context, we see some of the earliest years of facilities being in use (namely years between 1855 and 1871, both years included) are not recorded in the digital archive we are using. Kristiana and Bergen were upgraded from *dollhus* to asylum^3^.

Our study period covers the decades from 1870s to and including 1920s, a period of time where the understanding of disease changes. With the advent of germ theory, our understanding of physical illness changes, and psychiatry in the 1920s seeks an event (such as schizophrenia) and considers different clinical paths for organic versus reactive psychosis. We provide an overview of some of the ideas considered at the time in Table 6.

When working with historical records, we need to bear in mind that our understanding of medicine changes throughout history and be careful not to force our contemporary understanding on the historical causes of different something and maybe consider an effect of stress. For instance, we know now that stomach ulcers are caused by helicobacter infections but that was not known in 1912, so the understanding is different and may be considered an effect of stress. As a result, the directors of the facilities may not be considering it a cause for admission linked to infectious diseases. Awareness of these factors is critical when using this dataset for further research.

## Methods

### Demarcation

The reports cover 1872 to 1929, both years included (58 years). The first two years were the responsibility of the *Departmentet for det Indre* (Interior Ministry) while the later reports were the responsibility of the *Medisinaldirektøren* (Medical Director). The earliest report is available in 1872 (see the supporting information for an overview of the sources used). The cutoff date is 1929 because no admission records stratified by cause for admission are available after this year due to reporting changes. This change is evidenced from the next available report on the facilities after 1929 (emphasis and translation ours).

“Beretningen for året 1935 er gitt en noe annen form enn før vesentlig på grunn av at innberetningsskjemaet for asylene er blitt forandret. (…) Av tabellene over de patienter som for første gang er innlagt i norske asyl, er sloifet tabell 8 I over sykdommens varighet ved innleggelsen og tabell 11 over de opgitte årsaker til sinnssykdom.» [The reporting for the year 1935 has a different format than previously as a result of changes to the reporting form used in facilities. Of the tables of patients who are admitted to Norwegian facilities, table 8 concerning the duration of illness at admission has been removed as well as table 11 concerning the reasons provided regarding mental illness.] – Sinnssykeasylenes virksomhet 1935 (NOS IX, emphasis and translation ours)

Additionally, medical reports of the time note that the record from 1930 is not available.

“I medisinalstatistikken for 1930 er tatt inn noget flydigere Oplysninger om sinnssyke enn tidligere på grunn av Byrået ikke har anledning til å trykke beretningen om sinnssykeasylenes virksomhet for 1930» [The medical statistics for 1930 include more extensive information on mentally ill (patients, ed.) than previously because the city council does not have reason to publish reports on the activities of facilities for 1930] – Sundhetstilstanden og medisinalforholdene 1930 (NOS IX 2, emphasis and translation ours)

Each report we use contains some twenty tables covering all aspects of treatments (constraints), catchment areas, deaths, accounting etc. as well as written descriptions by the directors of each facility. We are interested in the table related to admissions stratified by cause.

### Data extraction and post-processing

The reports are obtained as a scanned portable document format (PDF) data from Statistics Norway (https://www.ssb.no/historisk-statistikk/emner/helse-og-sosiale-forhold)^5–61^. Each file was named according to its year and stored in a dedicated project directory. These reports included a table containing information on cause for admission, location, capacity of the facility and its classification. These reports are listed in full in the Supplementary Data Appendix.

Although PDF documents conform to an open data standard that ensures readability, they are not provided in a format that can be directly imported to analysis software for quantitative analysis. Thus we need to first extract from each report the table of interest (see Supplementary Data Appendix). We created two variables in the data: one which denotes which report the information is sourced from and one which denotes which table in the report was used.

### Optical character recognition processing

As there are a large amount of tables, we extracted the data using optical character recognition (OCR) techniques rather than manual extraction. OCR is a technology that converts different types of texts, such as handwritten, printed or scanned documents, into editable text. Regarding OCR, we initially attempted to create an open source, in-house solution using Tesseract. However as a result of time constraints and the horizontal divisions in the table not being read well, we found it necessary to instead utilise an “out of box” solution. For this we used the proprietary software ABBYY Finereader. The in-built OCR feature provides text recognition which was applied on all the tables (we printed the tables in A3 format before using OCR).

### Manual transcription

While the OCR feature works well on text, our extraction of tables turned out to be more complicated for recognition, namely: in lots of cases columns were merged and needed to be manually split and for certain years the scanned documents were of poor quality and so were output as images rather than extracted which required a fully manual transcription.

### Post-OCR review and correction

All OCR outputs were systematically reviewed for merged or misaligned columns, missing cells, and character misrecognition which was manually corrected. Where uncertainty remained, values were cross-checked against the original PDF scans.

### Footnotes

We added information contained in footnotes below the tables. Only where additional information was found did we add it: this means when counts for admissions are not provided in the footnotes, we do not include them. The information in the footnotes was not granular enough to adjust the counts in the original data set to incorporate the information from the footnotes so we have included them in addition. This means the counts are slightly higher than when the footnotes are not included, however, we determined the additional information provided was useful as it gives nuance to causes similarly to that found earlier in the data. We created a variable denoting whether the information was from the footnote (value “YES”) or solely from the table (value “NO”) which allows users of the data to filter out the footnotes if they want. The number of inclusions by source are given in Table 2.

**Table 2:** Data sources and dimensions following extraction and cleaning. The count for observations includes the footnotes.

Source	Years	Period	Observations	Footnotes
NOS I	8	1872–1879	8354	18
NOS II	4	1880–1883	5637	18
NOS III	15	1885–1898	24756	303
NOS IV	3	1899–1903	9003	122
NOS V	8	1904–1911	12432	38
NOS VI	6	1912–1917	10750	1
NOS VII	7	1918–1924	13310	19
NOS VII	5	1925–1929	2261	11

### Classification of facilities and capacity at facilities

The reports contain additional information which enables us to determine how the facilities are run. We added five categories of facility type: 1) Council (17 facilities), 2) Private (three facilities), 3) State (four facilities), 4) Incarceration (two facilities), and 5) Hospital (two facilities). Incarceration refers to what would now be known as a high-security psychiatric hospitals of which two are known to be such a type. The remaining facilities’ types were determined from the full name with the exception of *Lier* which was never listed with a full name but is contemporarily a hospital and so has been classified as such. The classification can be found in Table 3. Other researchers have used information on funding of the facilities to classify them into three economic levels (wealthy, mid, and poor)^1^.

**Table 3:** Facilities and their location as well as the first and last years they occur in the data set.

Facility	Location	Full Name	Type	First	Last
Bergen	Teatergaten 43, 5010 Bergen	Bergen kommunale Asyl	Council	1872	1890
Blakstad	Strandveien 35, 1392 Vettre	Blakstad Akershus Amts kommunale Asyl i Asker	Council	1904	1929
Bratsberg	Hakasteinvegen, 3728 Skien	Bratsberg amtskommunes asyl i Solum	Council	1909	1917
Dale	Daleveien 581, 4329 Sandnes	Dale Stavanger amtskommunes og Stavanger bys asyl i Hetland	Council	1913	1929
Dedichens	Dr. Dedichens vei 28, 0675 Oslo	Dr. Dedichens Asyl, privat, ved Kristiania (Oslo)	Private	1901	1929
Dikemark	Sykehusveien 15, 1385 Asker	Dikemark Kristiania kommunale Asyl i Asker	Council	1905	1929
Eg	Andreas Kjærs Vei 93, 4615 Kristiansand	Eg Statsasyl ved Kristiansand	State	1881	1929
Faret	Ulefossvegen 55, 3710 Skien	Faret Telemark fylkeskommunes asyl i Solum	Council	1918	1929
Fastings Minde	Haakon Sheteligs plass 11, 5007 Bergen	Fastings minde kommunalt asyl i Bergen	Council	1918	1923
Gaustad	Scognsvannsveien 21, 0372 Oslo	Gaustad Statsasyl ved Kristiania (Oslo)	State	1872	1929
Kriminalasylet	Kongens gate 95, 7012 Trondheim	Kriminalasylet i Trondhjem	Incarceration	1895	1929
Kristiana	Storgata 36, 0182 Oslo	Kristiania kommunale Asyl	Council	1872	1908
Kristiansand	Trondenskjolds gate 52, 4612 Kristiansand	Kristiansands kommunale Asyl	Council	1873	1929
Lier	Fossbakken 38, 3403 Lier		Hospital	1926	1929
Møllendal	Møllendalsveien 69, 5009 Bergen	Møllendals Asyl privat ved Bergen	Private	1872	1929
Neevengården	Sandviksleitet 1, 5036 Bergen	Berens kommunale Asyl i Bergen	Council	1891	1929
Opdøl	Opdølvegen, 6450 Hjelset	Oppdøl Romsdals amtskommunes asyl i Bolsø	Council	1913	1929
Oslo	Grønland 28, 0188 Oslo	Oslo Hospitalstiftelses Asyl i Kristiania (Oslo)	Hospital	1873	1929
Østmarken	Østmarkveien 15, 7040 Trondheim	Østmark kommunalt asyl for trondhjem by og de 2 Trøndelagfylker	Council	1919	1929
Prestsæter	Presteseter 1, 2840 Reinsvoll	Prestsæter Kristians amtskommunes asyl i Vestre Toten	Council	1913	1929
Reitgjerdet	Brøsetvegen 100, 7046 Trondheim	Reitgjerdet statsasyl ved Trondhjem	Incarceration	1923	1929
Rønvik	Kløveråsveien 1, 8076 Bodø	Rønvik Statsasyl ved Bodø	State	1902	1929
Rosenbergs*	Øvergaten 29, 5003 & Møhlenprisbakken 1, 5007 Bergen	Rosenbergs private Asyl i Bergen	Private	1872	1929
Rotvold	Arkitekt Ebbells veg 22, 7053 Ranheim	Rotvolds Statsasyl ved Trondhjem	State	1872	1929
Sanderud	Peter Skredders veg 34, 2312 Ottestad	Sanderud Hedemarkens amtskommunes asyl i Stange	Council	1908	1929
Stavanger	Klubbgata/Hospitalsgata, 4013 Stavanger	Stavangers kommunale Asyl	Council	1873	1893
Trondhjem	Erling Skakkes gate 66, 7012 Trondheim	Trondhjems (Hospitalsstiftelse) kommunale Asyl	Hospital	1872	1919
Valen	Sjukehusvegen 26, 5451 Valen	Valen Søndre Bergenhus amtskommunes asyl i Fjelberg	Council	1910	1929
Veum	Veum alle 3, 1615 Fredrikstad	Veum Smaalenenes amtskommunes asyl i Glemming	Council	1914	1929

*Rosenbergs consists of two buildings Rosenberg and Øvregaten and thus has two addresses but was run by the same manager.

**Fig. 2:**
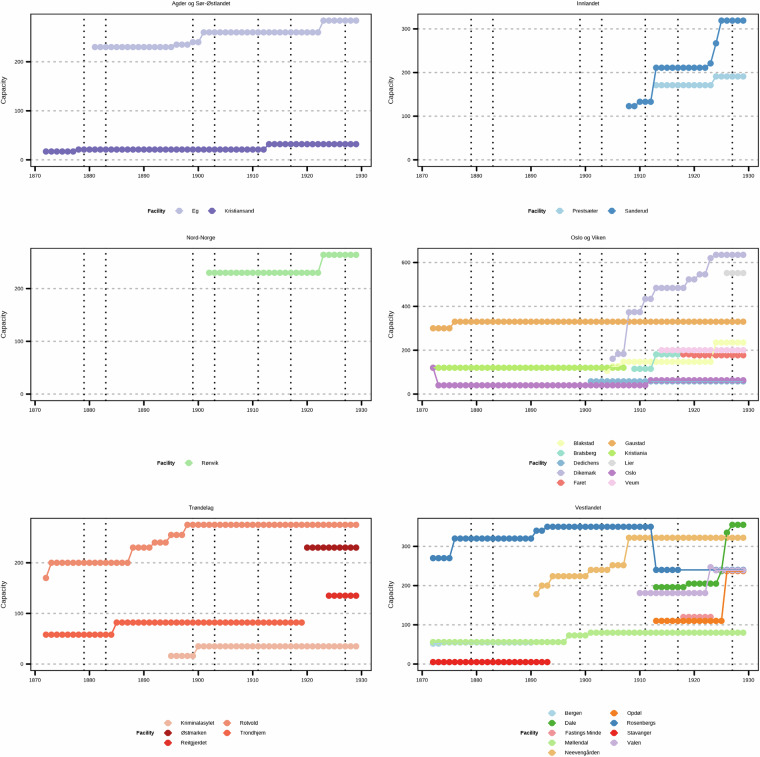
Capacity (for all patients, not stratified by biological sex) at facilities grouped by NUTS region.

The reports contain additional information on the capacity of the facilities. This provides us with estimates of how much treatment is possible to provide. To provide a sense of comparison with our data, the capacity at *dollhus* was said to be 72 patients in 1814. Note that the total admission may exceed the capacity of how much treatment is possible to provide as some patients are discharged. The capacity is important if we wish to consider rates rather than counts for admissions, as it provides us with a denominator. The population of Norway during the study period is given as supporting information. We showcase the capacity in Fig. 2 (please see the Supplementary Data Appendix Figure 9 for the total capacity) where we see that some areas in Norway do not have any capacity for mental health treatment in the first decades of the study period. The following three quotes reflect the concerns about limited capacity at the time.

“*The want of accommodation is more and more* felt, although during these latter years great sacrifices have been made both by the Government and by certain cities to meet the difficulty.” – (emphasis ours^62^)

“Consequently in the more prosperous and densely populated sections of the country it has been necessary to place the lunatics, *who cannot be received in the asylums*, under the care of persons making a business of boarding a greater number of patients in rooms that are especially arranged for this purpose. These establishments, commonly called “colonies,” do not always satisfy the requirements of modern nursing of such patients.” – (emphasis ours^63^)

“We are striving to abolish these “colonies” and to *provide room in asylums for most of these patients*, so that only those who are suitable should get private care in families.” – (emphasis ours^64^)

The facilities have differences in terms of patients (women-focused, men-focused, and non-segregated) as well as temporal differences as they open and close at different points in the study period (Fig. 2 and Table 3). For reporting gaps of a single year, we can assume that it is not the case that the facility closed and then re-opened but rather that the data is not reported for that specific year.

### Cleaning facility names

**Fig. 3:**
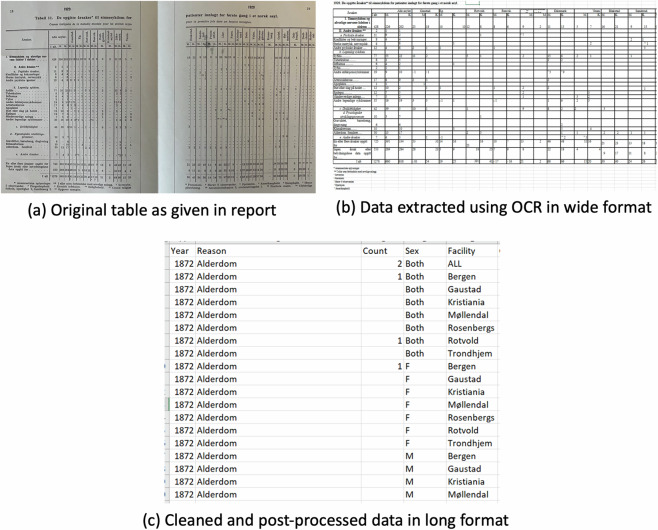
Example of data extraction process (year 1872 shown). The table is printed (3a) and read for OCR, which gives us an electronic version of the Table (3b), and finally all manually cleaned data in data base format (3c).

**Fig. 4:**
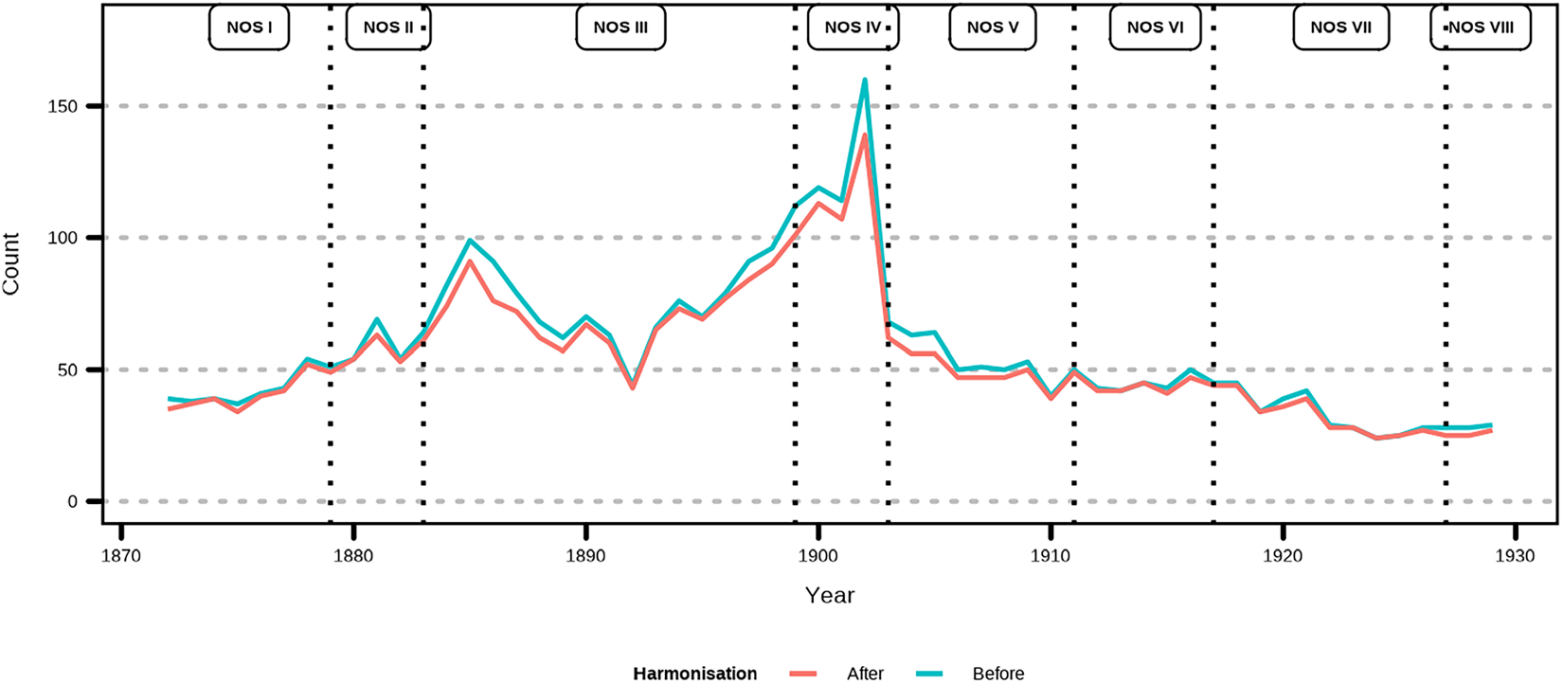
Number of unique causes listed in the tables as extracted (before) and after harmonizing terms (after).

We obtained a rich data set for examining the historical epidemiology of mental health which spans multiple decades and regions and contains information on multiple causes (Fig. 3). Each row in the data set tells us the number of admissions for specific cause stratified by year, biological sex, cause for admission (reason), and facility. We also note the sources as we suspect the different sources affect the record keeping. For instance, from NOS V onwards (i.e., from 1903), the categorization is simplified (Fig. 4), which gives a lower number of causes. This is partially rectified by the inclusion of information from the footnotes.

Within the records, the facility names were not consistent throughout. Our cleaning of facility names was done as outlined in Table 4. The cleaning of facility names involved manual review of all unique facility names, mapping historical and modern spellings, and assigning standard names used consistently across data set (e.g., “Christianssand” was standardized to “Kristiansand”). After extraction and cleaning, the data contains 29 unique facilities distinguished by the names given in Tables 3, 4.

**Table 4:** Harmonisation of facility names.

Facility	Alternative spellings	Amendments
Bergen	Consistent throughout	
Blakstad	Consistent throughout	
Bratsberg	Bratsbergs	Additional “s” removed
Dale	Consistent throughout	
Dedichens	Consistent throughout	
Dikemark	Consistent throughout	
Eg	Consistent throughout	
Faret	Consistent throughout	
Fastings Minde	Fastings minde	Capitalised
Gaustad	Consistent throughout	
Kriminalasylet	Kriminal asylet, Kriminalasylet, Krimi-nal-asylet	Removed superfluous hyphens and spaces
Kristiania	Cliristiania, Christiania	Replaced misreading of “li” as “h” and amended spelling from “ch” to “k” to match first instance which is “Kristiania”
Kristiansand	Kristiansands, Christianssand, Chris-tiansand, Cliristianssand, Christians-sand	Replaced misreading of “li” as “h”, additional “s” removed, and amended spelling from “ch” to “k” to match first instance which is “Kristiansand”
Lier	Consistent throughout	
Møllendal	Mallendal, Møllen dal, Mallendal, Møllen-dal	Removed superfluous hyphens and spaces and amended misreading of “a” and “o” as “ø”
Neevengården	Newen-gaarden, Newengaarden, Neevengaar-den	Removed superfluous hyphens, amended misreadings of “w” as “v”, and replaced “aa” with “å”
Opdøl	Opd|øl, Opdal	Removed superfluous bar and amended misreadings of “a” as “ø”
Oslo	Oslo hospit.	Removed reference to “hospital”
Prestsæter	Presteseter	Amended to match first instance which is “Prestsæter”
Reitgjerdet	Consistent throughout	
Rosenbergs	Rosenberg, Rosen bergs, Rosenborgs, Kosenbergs	Amended to match “Rosenbergs”
Rotvold	Rotvoid, Rolvold	Amended misreadings of “I” as “i” and “t” as “I”
Rønvik	Consistent throughout	
Sanderud	Consistent throughout	
Stavanger	Consistent throughout	
Trondhjem	Trond hjem, Trondhjems, Trond-hjem, Trondhjems, Trond-hjem-, Trondjein, Trondljem	Removed superfluous hyphens and spaces, additional “s” removed, and amended misreadings of “in” and “m” and “li” as “h”
Valen	Consistent throughout	
Veum	Consistent throughout	
Østmarken	Østmark	Added “en” to the end

### Post-processing

#### Understanding specific causes for admissions

The causes listed in the reports cannot necessarily be understood as given but require an understanding of the field of mental health at the time. Many of the causes are the same as those found in Leidinger *et al*.^65^, which makes sense as Norwegian mental health ideals were inspired by Germany as well as both being Germanic languages. Sweden’s approach to mental health was in turn inspired by Norway^66^. Weakness is a general term often used in the nineteenth century^65,67^. It may thus not necessarily correspond to fatigue. Other terms for post-viral mental health conditions were in use in the 1800s in addition to those already mentioned. They are according to Honigsbaum and Krishnan^68^: neuralgia, nerve exhaustion, psychoses, anxiety, and *influenza nervosa*. The work by Abdalla *et al*.^69^ on textual analysis of medical journals across centuries suggest that (assuming the United States of America dominance present in the data source used) part of what makes up the “hereditary” category is abortions. Additionally, *slag* refers to stroke rather than a knock^70^. However, *trauma* is likely related to physical rather than mental injury. For counts and translation of the terms, please see the supporting information.

### Harmonisation of causes for admissions

In addition to harmonizing the facility names, we harmonized certain causes for admissions found within the records. Examples of what we did are found in Table 5 and an example of what this looks like for certain causes is shown graphically in the supporting information. Because we are considering admissions, we do not distinguish between chronic and acute conditions and have merged those thus removing the distinction. The full list of terms before and after harmonization are provided as supporting information in Supplementary Data Appendix.

**Table 5:** Types of harmonization applied to causes for admission.

Harmonisation	
Spelling	Example 1: Syfilis > Syphilis
Update older terms	Example 1: Skindsot > Tuberkulose
	Example 2: Klimakterium > Overgangsalder
Sensitivity	Example 1: Idioti > Mental Handicap
Simplify terms	Example 1: Forkjølelse efter Falden i Vandet > Forkjølelse
	Example 2: Stærk Hede paa hovedet i (i et Bageri) > Solstik
	Example 3: Sinnssykdom og alvorlige nervøse lidelser i slekten > Arv
Collapsing	Example 1: Onani > Masturbation
	Example 2: Selvbesmittelse > Masturbation

We chose to harmonise *blegsot* to be the same as *klorose*, which was a women’s health concern at the time according to Bondevik^71^ who further notes that *neurasthenia* and *hysteria* are also women’s health concerns. Others corroborate this and suggest they be considered as part of the same^72^, but we have chosen to keep them separate. We have merged typhus and typhoid^73,74^.

### Addition of auxiliary information

#### Classification of causes

Contemporary hospital records contain classification codes used to determine which health problems are found among the patient. The earliest attempts at standardisations of causes are the Nomenclature of Diseases by the Royal College of Physicians from 1885 and the International Classification of Diseases (ICD) from 1901^75^. The ICD is still in use today and we classify our admissions data by the main headings from Bertillon (from which it was developed, see the supporting information for the categories). As researchers based in a research institute focused on infectious disease, we find Bertillon’s rule number 3 to be particularly useful:

“when among the two causes of death are in transmittable disease, it is preferable to assign the death to it, for statistics of infectious diseases are particularly interesting to sanitarian and it is important that they shall be as complete as possible” - as quoted in^75^.

To gain a deeper understanding of some of the obsolete medical terms for mental illness, the ideas of the time must be investigated. It should be noted that many of the terms used (and the ideas contained therein) are precursors of pseudoscience used to discriminate such as eugenics, phrenology, and social Darwinism and this may be a difficult topic to navigate. We have attempted to remove all offensive terminology from the data set. Some of the ideas championed by male psychiatrists are outlined in Table 6. Many of these ideas have since been abandoned but would impact the decision made with regards to admitting patients. Others such as Peritz^76^, Pinel^77^, and Kahlbaum^78^ have attempted to classify mental disorders. This provides a historical analogy of the Diagnostic and Statistical Manual of Mental Disorders (DSM), which is a classification scheme focused on mental health disorders, with a narrower scope than the ICD. The two schemes may not be in alignment as their scope and use differs as well as their publishers (the World Health Organization is responsible for ICD and the American Psychiatric Association is responsible for DSM).

**Table 6:** Suggested ideas of mental health.

Year	Proponent	Idea
1810	Esquirol	monomania
1817	Parkinson	paralysis agitans (shaking palsy)
1835	Prichard	moral insanity
1843	Sweetzer	mental hygiene**
1843	Braid	hypnosis
1854	Bailarger	dual-form innsanity*
1854	Falret	circular insanity*
1857	Morel	(social)degeneration theroy
1859	Briquet	somatisation disorder
1869	Beard	neurastenia
1870s	Charcot	hysteria
1873	Clouston	adolescent insanity†
1887	Kraepelin	dementia precox → Kraepelinian dichotomy
1892	Pick	frontotemporal dementia
1899	Freud	psychoanalysis
1901	Sommer	psychohygiene**
1911	Bleuler	schizophrenia
1912	Jung	analytical psychology
1913	Watson	behaviourism
1912	Stern	intelligence quotient testing

*same condition.

**same condition.

^†^Kahlbaum considered “juvenile insanity”.

In addition to this, much of the understanding has changed and so the causes cannot be interpreted *prima facie*. The temporal coverage of the data contains multiple medical paradigms of miasma, contagion and bacteriology. Syphilis likely does not mean syphilis^76^. Uterine diseases are considered to be a mental health rather than physical condition^79^. Fractures may have a deeper meaning^80^. Roberts^74^ suggests that hydrocephaly is meningitis, laryngitis is diphtheria, and puerperal disease (called puerperium in our data) is streptococcus infections. It should be noted that the data covers the period immediately following Semmelweis and their ideas on infection control. These ideas took time to permeate but have reduced puerperium in the decades following significantly. We merged typhus with typhoid fever following Smith^73^. As with the causes for admission, listing the idea considered by male psychiatrists at the time is not to be seen as an endorsement of medical ideas considered at the time but rather an attempt to provide transparency.

### Location of facilities

We added the locations of facilities (longitudinal and latitudinal positions) as well as their contemporary nomenclature of territorial units for statistics (NUTS) regions. This is due in part to their potentially being changes in regional definitions (administrative divisions such as “amt”) in the data set and we are unaware of a good method for dealing with temporal changes in regional definitions.

## Data Records

The structured data on mental health admissions to Norwegian mental health facilities from 1872 to 1929 described in this Data Descriptor is available from Zenodo at 10.5281/zenodo.15101866^81^. It contains. The dataset was constructed through OCR extracted and manual curation of historical government reports. The data is harmonized to support historical epidemiological analysis, particularly around post-viral mental health conditions.

### Directory structure and file contents

**/data**

- mental_health_cleaned.csv: Final harmonized dataset. Each row represents admissions grouped by year, biological sex, facility and cause for admission. Cause terms are standardized using Bertillon classification.
- precleanedasylumdata.csv: OCR-extracted tables from the original reports (Fig. 3), prior to cleaning and harmonization. Useful for tracing transformation and transparency.
- bertillon_mapping.csv: Mapping of historical cause descriptions to Bertillon categories and primary themes (e.g., infectious disease, women’s health).
- cause_categories.csv: Thematic grouping of causes used in visualization and analysis.

**/reference_data**

- comparison_sweden.csv, comparision_wales.csv, comparison_oregon.csv: Benchmark datasets used for historical comparison in Fig. 7 of the manuscript.

**/scripts**

- script.R, reason.R, facility.R, timeline.R: R scripts used for data cleaning harmonization, and figure generation. All scripts were annotated and reproducible.

**/docs**

- README.txt: Documentation of file structure, data dictionary, processing steps, and software dependencies.

All datasets are provided in UTF-8 encoded.csv format. No proprietary software is required. The R scripts were developed in R version $\ge$4.2 and use open-source libraries.

Missing values for locations (NUTS, addresses, and latitude/longitude coordinates) are due to the facility total counts given in the original tables (named “ALL”). This is also the case for type of facility (captured in variable “type”), as when considered combined the facilities lose this nuance.

## Technical Validation

To ensure the reliability and consistency of the dataset, several validation procedures were carried out during data extraction, cleaning, and harmonization.

### OCR accuracy and manual correction

The primary source data consisted of scanned PDF versions of historical annual reports from Norwegian mental health facilities (1872 to 1929). OCR was performed using ABBYY FineReader due to its superior performance over open-source alternatives (e.g., Tesseract) in handling complex tables with horizontal and vertical lines. Despite this, many tables required manual post-processing to correct misread columns, merged cells, or non-standard table formats.

For reports with poor scan quality (e.g., smudging, skewed pages), OCR failed entirely, and a manual transcription process was undertaken. These cases were flagged internally to ensure transparency. Additionally, footnotes below the tables were extracted and included where possible; a binary variable was added to denote the source of each observation.

### Consistency checks and harmonisation

Facility names were standardised to ensure consistency across the dataset. Common OCR errors and historical spelling variations (e.g., “Christianssand” vs “Kristansand”) were cleaned using a predefined mapping (see Table 4). Similarly, cause of admission terms were harmonised using Bertillon’s classification of causes of death. Ambiguous or obsolete medical terms were reviewed using secondary sources and medical lexicons to ensure appropriate categorisation.

The dataset was further validated by cross-checking total admission counts per year and per facility against the source tables. Known missing years (e.g., 1899) were documented, and the lack of data for certain periods (e.g., 1930–1935) is due to archival changes in reporting format, as discussed in the Methods section.

### Dataset structure and completeness

The final cleaned dataset contains 89,438 observations and 27 variables. Each row represents an admission record stratified by year, facility, biological sex, and cause(s) of admission. The completeness and structure of the dataset allows for reuse and historical comparison. Metadata and a detailed data dictionary are provided in an accompanying README.txt file.

## Usage Notes

In this work we have attempted to provide a transparent description of our work extracting data from reports and creating a dataset from this information that can be used in further historical epidemiology investigations. This is to enable other researchers to quality control as well as replicate the work performed. However, the report from 1899 is truncated and to obtain the table, this required an on-site visit to the Statistics Norway offices. This illustrates an issue with panning for gold in the digital archives, if the pan is broken, the gold cannot be extracted even if it is known to be there.

Because we are a pandemics research centre, we are particularly interested in admissions due to infectious disease. This means our categorisation and collapsing of causes will be different than researchers from other disciplines. We provide in the supporting information an overview of our harmonisation, both such that others can reproduce our work but also so researchers with other interests can determine where to change our method to develop a different version of the data for themselves.

An analysis of a similar temporal period (1850 to 1912) determined that temporal changes in terminology can be rectified by mapping onto ICD codes^82^. However, Anderton and Hautaniemi Leonard^77^ note in the changing understanding of phthisis and consumption becoming tuberculosis, “changing certainty of the diagnosis of obscure by using ICD codes rather than literal causes.” For this reason, we consider also the ICD-4 codes^83^, giving us a sense of the understanding after Bertillon. An attempt to provide historical ICD-10 codes known as ICD-10-H is ongoing. ICD-10-H was not available at the time of writing but was mentioned to the authors at a conference as being released later in the year^84^. While useful, it seems to have greater relevance for projects with study periods which are not covered by ICD (we have Bertillon followed by ICD-1 in 1900, ICD-2 in 1909, ICD-3 in 1929, and ICD-4 in 1929). Additionally, ICD-10-H is constructed with deaths in focus while our interest is morbidity rather than mortality. For the sake of completeness, we note that similar work is ongoing for classifying occupation (HISCO; Historical International Social Class Scheme) historically which may be an interesting line of inquiry for researchers interested in other tables from the reports. A comparison between admissions and contemporary ICD-10 codes has been done for Sweden^66^ and Wales^85^ and would be possible for our data also.

### Comparisons with other locations

Similar records from Oregon, US^86^ provide a sense of the burden of disease of mental health, though for comparison we consider the proportion of the total admissions as we have multiple facilities. We examine the years 1895 and 1896 and compare the proportions for those causes which exist in the American records also. Of those causes that are listed in the Oregon data (Fig. 7, middle panel), we see patterns that are not too dissimilar while including also the ICD-forwarded data from Sweden and Wales, we see a more pronounced difference. Writing at the same time as the data, Habgood^87^ suggests the burden of mental health in Norway is lower than it was in England and that the requirements for being admitted to a facility were stricter in the latter.

## Discussion

Regarding Norway, researchers have noted “there are valid reasons why one should use the present-day administrative divisions by back-projecting them to earlier periods: Access to the sources might be easier, the prevalence principle decreeing that source material is to be organised by the present-day administrative divisions. Thus, a discussion of the choice of region is imperative, but is often lacking in commissioned research”^88^. We have elected to use the contemporary NUTS regions for lack of an obvious historical choice. Efforts to create geographical information systems data bases that can handle administration boundary region changes are ongoing^89,90^ but not available for all of Norway yet. For this reason, we leave determination of catchment areas like the facilities as an exercise for further investigation. The information should be available in the tables not extracted however this requires additional panning of digital archive, and the information may not be available for years.

**Fig. 5:**
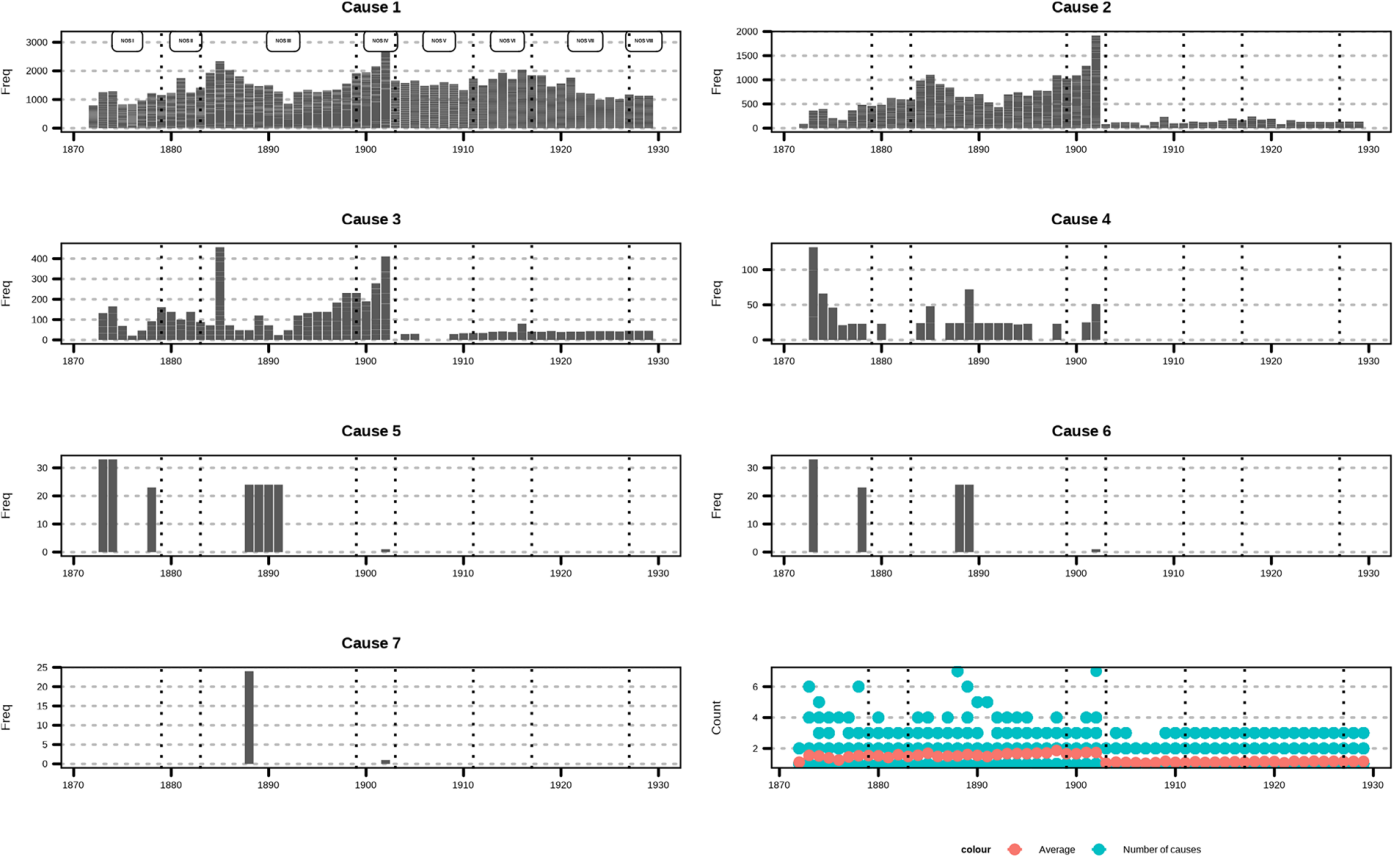
Number of causes listed.

As regards classification of causes, we have considered the first cause (an overview of the number of causes listed can be found in Fig. 5, where the structure introduced by NOS V in 1903 is again evident) as the primary cause. If we were working with morbidity data this would be the case, as the final cause listed on the death certificate is a primary cause. We provide all the causes listed as the original list reported (variable “Reason”) as well as the causes individually in order.

The particular line of inquiry of interest to our research group, the link between influenza infection and mental health disorders is currently not fully established but speculated to exist^91^. Other research groups are also investigating such questions historically^92^ and our data may provide useful for determining the historical burden of mental health. To the best of our knowledge, while there are efforts to provide historical classifications of statistics (see previous mentions of e.g., HISCO), there is not effort to extend the global burden of disease study backwards in time. We believe this work to be of particular interest to other researchers who have used the same data sources for specific questions^93–99^.

**Fig. 6:**
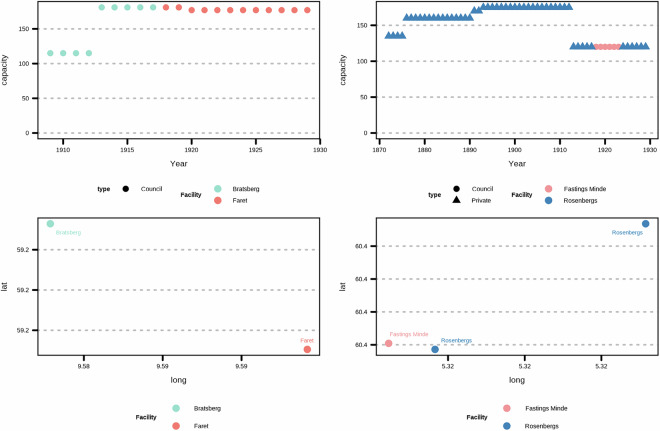
Facilities with similar capacity but different locations.

Based on inspection of data alone, you may be tempted to conclude based on reported capacity (Fig. 6) that some of the facilities are the same (namely Bratsberg and Faret (left panels) and Fastings Minde and Rosenberg (right panels), however further inspection of their location (Table 3) illustrates the necessity of subject matter expertise when working with historical data as additional information is required before it is possible to conclude that they are the same. As supporting information, we provide a list of further reading reflecting the research landscape at the time of writing intended to be helpful for contextualising the dataset for uses (secondary literature references). We also suggest Norsk Kulturråd^100^ for greater understanding of archives and laws in Norway.

**Fig. 7:**
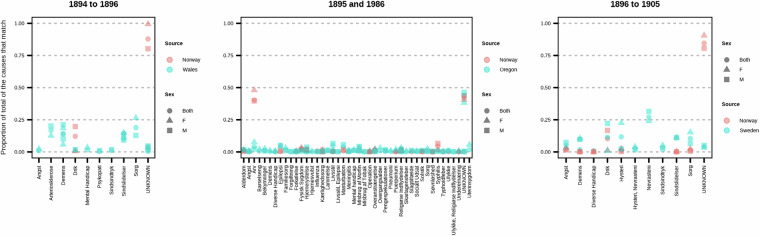
Comparison of matching causes for admission for select years and locations, sources^66,85,86^.

## Supplementary information

Construction and curation of a data set of historical mental health incidence in Norway

## Data Availability

All R scripts used for data extraction, cleaning, harmonisation, and figure generation are included in the Zenodo archive alongside the dataset^81^. These include the scripts script.R, facility.R, reasons.R, and timeline.R. The code is annotated and organised to ensure full reproducibility. No external code repositories were used.
