# Supplementary material for: Construction and curation of a data set of historical mental health incidence in Norway
Source: Sci Data. 2025 Aug 28;12:1510. doi: 10.1038/s41597-025-05795-y (PMC12394546; doi:10.1038/s41597-025-05795-y)
Supplement: Supplementary file 1 — Construction and curation of a data set of historical mental health incidence in Norway [file 41597_2025_5795_MOESM1_ESM.pdf]

Construction and curation of a data set of historical mental health incidence in Norway

A. O. Blinkova[1], U. Khakurel[1], H. G. Gaddy[1,2], S.-E. Mamelund (svenn-erik.mamelund@oslomet.no)[1], and M. Bekker-Nielsen Dunbar[1]

[1]*Centre for Research on Pandemics & Society, Oslo Metropolitan University*
[2]*Department of Economic History, London School of Economics*

This document provides additional information in support of the manuscript.

1 Sources used

1.1 Data sources from Statistics Norway (main source)

From each report below is extracted the table of admission to mental health facility by cause for admission and location (using optical character recognition) as well as the capacity of the facility and its classification (manually extracted). The specific tables used are noted in the data set (and shown in the lower panel of Figure 10).

1. Norway's official statistics (NOS) I
   (a) Oversigt over Sindssygeasylernes Virksomhed i Aaret 1872 (NOS I C5B 1872) [Departementet for det Indre, 1873]
   (b) Oversigt over Sindssygeasylernes Virksomhed i Aaret 1873 (NOS I C5B 1873) [Departementet for det Indre, 1874]
   (c) Oversikt over Sindssygeasylernes Virksomhed i Aaret 1874 (NOS I C5B 1874) [Departementet for det Indre, 1875]
   (d) Oversigt over Sindssygeasylernes Virksomhed i Aaret 1875 (NOS I C5B 1875) [direktøren for det Civile Medicinalvæsen, 1876]
   (e) Oversigt over Sindssygeasylernes Virksomhed i Aaret 1876 (NOS I C5B 1876) [direktøren for det Civile Medicinalvæsen, 1877]
   (f) Oversigt over Sindssygeasylernes Virksomhed i Aaret 1877 (NOS I C5B 1877) [direktøren for det Civile Medicinalvæsen, 1878]
   (g) Oversigt over Sindssygeasylernes Virksomhed i Aaret 1878 (NOS I C5B 1878) [direktøren for det Civile Medicinalvæsen, 1879]
   (h) Oversigt over Sindssygeasylernes Virksomhed i Aaret 1879 (NOS I C5B 1879) [direktøren for det Civile Medicinalvæsen, 1880]

2. NOS II

   (a) Oversigt over Sindssygeasylernes Vriksomhed i Aaret 1880 (NOS II C5B 1880) [direktøren for det Civile Medicinalvæsen, 1883]
   (b) Oversigt over Sindssygeasylernes Virksomhed i Aaret 1881 (NOS II C5B 1881) [direktøren for det Civile Medicinalvæsen, 1884a]
   (c) Oversigt over Sindssygeasylernes Virksomhed i Aaret 1882 (NOS II C5B 1882) [direktøren for det Civile Medicinalvæsen, 1884b]
   (d) Oversigt over Sindssygeasylernes Virksomhed i Aaret 1883 (NOS II C5B 1883) [direktøren for det Civile Medicinalvæsen, 1884c]

3. NOS III

   (a) Oversigt over Sindssygeasylernes Virksomhed i Aaret 1884 (NOS III 9) [direktøren for det Civile Medicinalvæsen, 1885]
   (b) Oversigt over Sindssygeasylenes Virksomhed i Aaret 1885 (NOS III 28) [direktøren for det Civile Medicinalvæsen, 1886]
   (c) Oversigt over Sindssygeasylernes Virksomhed i Aaret 1886 (NOS III 48) [direktøren for det Civile Medicinalvæsen, 1887]
   (d) Oversigt over Sindssygeasylernes Virksomhed i Aaret 1887 (NOS III 77) [direktøren for det Civile Medicinalvæsen, 1888]
   (e) Oversigt over Sindssygeasylernes Virksomhed i Aaret 1888 (NOS III 103) [direktøren for det Civile Medicinalvæsen, 1890]
   (f) Oversigt over Sindssygeasylernes Virksomhed i Aaret 1889 (NOS III 125) [Medicinalstyrelsen, 1890]
   (g) Oversigt over Sindssygeasylernes Virksomhed i Aaret 1890 (NOS III 145) [Medicinaldirektøren, 1891]
   (h) Oversigt over Sindssygeasylernes Virksomhed i Aaret 1891 (NOS III 164) [Medicinaldirektøren, 1894a]
   (i) Oversigt over Sindssygeasylernes Virksomhed i Aaret 1892 (NOS III 189) [Medicinaldirektøren, 1894b]
   (j) Oversigt over Sindssygeasylernes Virksomhed i Aaret 1893 (NOS III 209) [Medicinaldirektøren, 1895]
   (k) Oversigt over Sindssygeasylernes Virksomhed i Aaret 1894 (NOS III 239) [Medicinaldirektøren, 1896]
   (l) Oversigt over Sindssygeasylernes Virksomhed i Aaret 1895 (NOS III 273) [Medicinaldirektøren, 1897]

(m) Oversigt over Sindssygeasylernes Virksomhed i Aaret 1896 (NOS III 296) [Medicinaldirektøren, 1898]

(n) Oversikt over Sindssygeasylernes Virksomhed i Aaret 1897 (NOS III 320) [Medicinaldirektøren, 1899]

(o) Oversigt over Sindssygeasylernes Virksomhed i Aaret 1898 (NOS III 331) [Medicinaldirektøren, 1900]

4. NOS IV

(a) Oversigt over Sindssygeasylernes Virksomhed i Aaret 1899 (NOS IV 2) [Medicinaldirektøren, 1901]

(b) Oversigt over Sindssygeasylernes Virksomhed i Aaret 1900 (NOS IV 29) [Medicinaldirektøren, 1902]

(c) Oversigt over Sindssygeasylernes Virksomhet i Aaret 1901 (NOS IV 58) [Medicinaldirektøren, 1903a]

(d) Oversigt over Sindsygeasylernes Virksomhet i Aaret 1902 (NOS IV 74) [Medicinaldirektøren, 1903b]

(e) Oversigt over Sindssygeasylernes Virksomhed Aaret 1903 (NOS IV 105) [Medicinaldirektøren, 1905]

5. NOS V

(a) Sindssygeasylernes Virksomhed 1904 (NOS V 1) [Medicinaldirektøren, 1906]

(b) Sindssygeasylernes Virksomhed, 1905 (NOS V 28) [Medicinaldirektøren, 1907]

(c) Sindssykeasylernes Virksomhet, 1906 (NOS V 56) [Medicinaldirektøren, 1908]

(d) Sindssykeasylernes Virksomhet, 1907 (NOS V 78) [Medicinaldirektøren, 1909a]

(e) Sindssykeasylernes Virksomhet, 1908 (NOS V 102) [Medicinaldirektøren, 1909b]

(f) Sindssykeasylernes Virksomhet, 1909 (NOS V 140) [Medicinaldirektøren, 1911]

(g) Sindsykeaylenes Virksomhet, 1910 (NOS V 158) [Medicinaldirektøren, 1912]

(h) Sindsykeasylenes Virksomhet 1911 (NOS V 190) [Medicinaldirektøren, 1913]

6. NOS VI

(a) Sindssykeasylenes virksomhet 1912 (NOS VI 6) [Medicinaldirektøren, 1914]

(b) Sindsykeasylenes virksomhet 1913 (NOS VI 42) [Medicinaldirektøren, 1915]

(c) Sindssykeasylenes virksomhet, 1914 (NOS VI 85) [Medicinaldirektøren, 1916]

(d) Sindssykeasylenes virksomhet, 1915 (NOS VI 109) [Medicinaldirektøren, 1917]

(e) Sinnssykeasylenes virksomhet, 1916 (NOS VI 166) [Medisinaldirektøren, 1920a]

(f) Sinnssykeasylenes virksomhet 1917 (NOS VI 185) [Medisinaldirektøren, 1920b]

7. NOS VII
   (a) Sinnssykeasylenes virksomhet 1918 (NOS VII 4) [Medisinaldirektøren, 1921]
   (b) Sinnssykeasylenes virksomhet 1919 (NOS VII 71) [Medisinaldirektøren, 1923a]
   (c) Sinnssykeasylenes virksomhet 1920 (NOS VII 88) [Medisinaldirektøren, 1923b]
   (d) Sinnssykeasylenes virksomhet 1921 (NOS VII 143) [Medisinaldirektøren, 1923c]
   (e) Sinnssykeasylenes virksomhet 1922 (NOS VII 156) [Medisinaldirektøren, 1925]
   (f) Sinnssykeasylenes virksomhet 1923 (NOS VII 162) [Overlægen for Sinnssykevesenet, 1925a]
   (g) Sinnssykeasylenes virksomhet 1924 (NOS VII 179) [Overlægen for Sinnssykevesenet, 1925b]
8. NOS VIII
   (a) Sinnssykeasylenes virksomhet 1925 (NOS VIII 17) [Overlægen for Sinnssykevesenet, 1927]
   (b) Sinnssykeasylenes virksomhet 1926 (NOS VIII 50) [Medisinaldirektøren, 1928]
   (c) Sinnssykeasylenes virksomhet 1927 (NOS VIII 84) [Medisinaldirektøren, 1929]
   (d) Sinnssykeasylenes virksomhet 1928 (NOS VIII 115) [Medisinaldirektøren, 1930]
   (e) Sinnssykeasylenes virksomhet 1929 (NOS VIII 162) [Medisinaldirektøren, 1931]

1.2 Additional data sources

The Nomenclature of Territorial Units for Statistics (NUTS) regions are administrative subregions used for statistical purposes. As Norway has a long, narrow shape, hexbins may be utilised for ease of visualisation (Figure 1) as they ensure the subregions are equisized. The full temporal change of admissions stratified by region is shown in Figure 2. Names of the NUTS regions are given in Table 1.

1.2.1 Bertillon

The categorisation groups considered by Bertillon are given in Table 2.

2 Admission causes

A translation of the causes in the data from Norwegian to English is provided in Table 3.

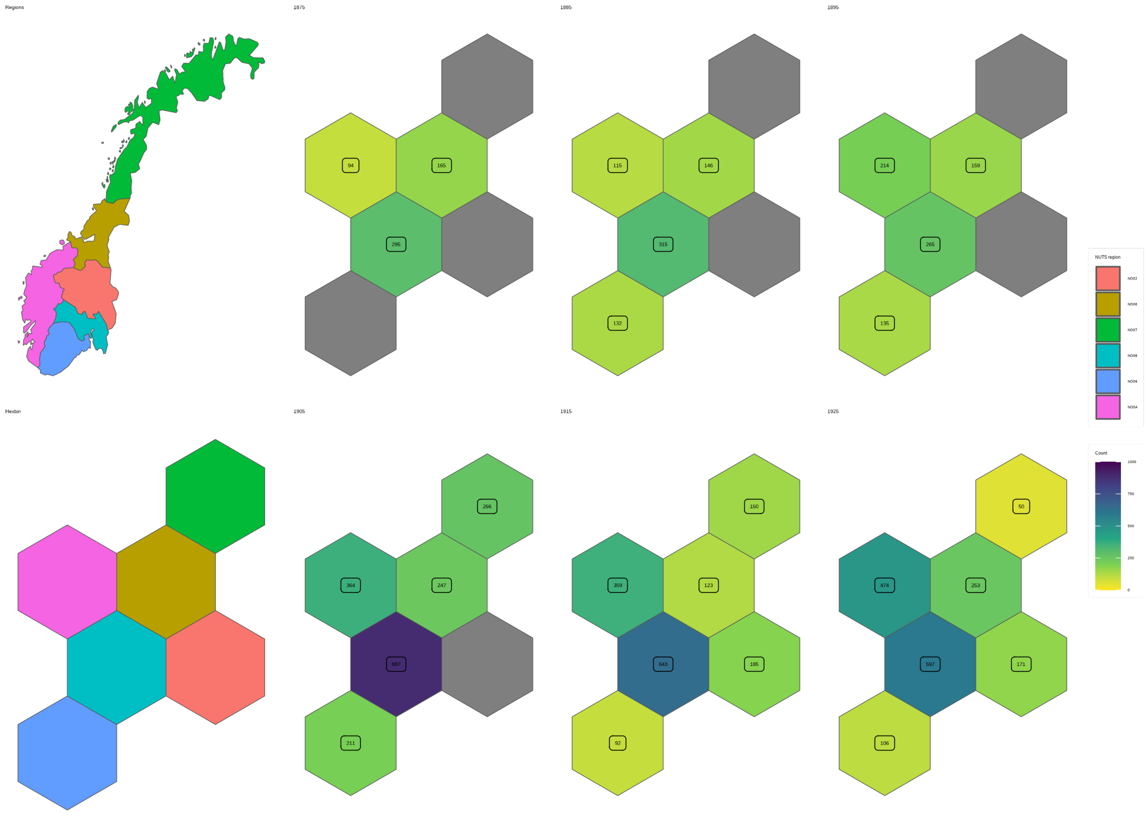

Figure 1: Snapshot (per decade) of admissions by NUTS region

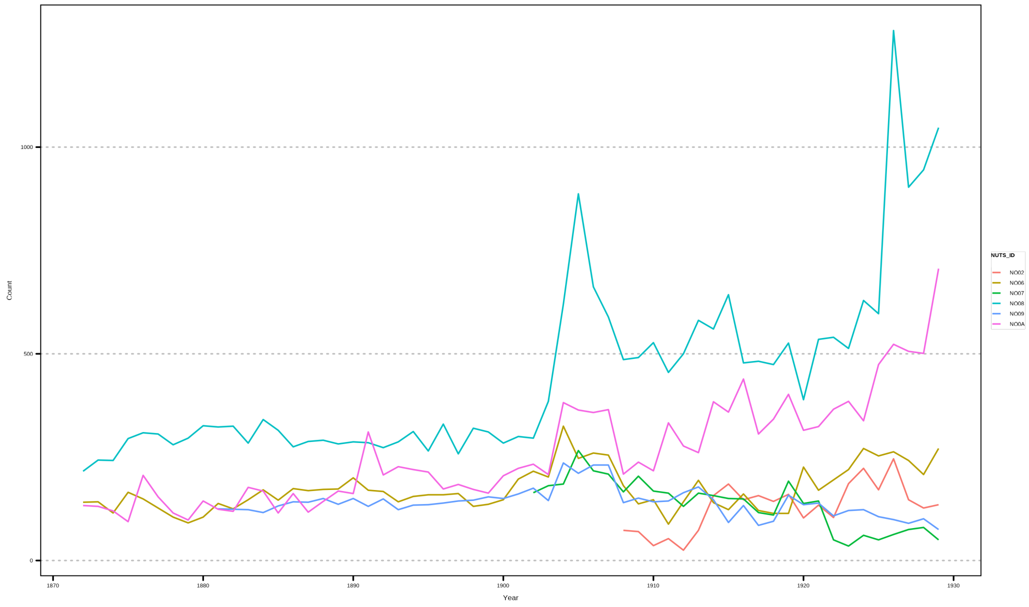

Figure 2: Admissions by NUTS region

Table 3: The frequency is the total frequency (all occurrences)

Cause	Frequency	Percentage	EN
Abort	522	0.442	Abortion
Absces	1	0.001	Abscess

Table 3: The frequency is the total frequency (all occurrences) *(continued)*

Cause	Frequency	Percentage	EN
Ærgelse	636	0.538	Regret
Afkræftelse	510	0.432	Ruled out
Alderdom	2430	2.056	Old age
ALL	1686	1.427	ALL
Amerikaophold	760	0.643	Emigration to the US
Anæmi	1362	1.153	Anemia
Angina	1	0.001	Angina
Angst	1530	1.295	Anxiety
Ankylose	40	0.034	Ankylosis
Anstrængelse	48	0.041	Physical exertion
Appendicit	110	0.093	Appendicitis
Årebetændelse	26	0.022	Phlebitis
Åreknuder	34	0.029	Varicose veins
Arteriosklerose	907	0.768	Arteriosclerosis
Artrit	1	0.001	Arthritis
Arv	11002	9.311	Hereditary
Ascaris	1	0.001	Worms
Asthma	99	0.084	Asthma
Atheromasi	251	0.212	
Bekymringer	635	0.537	Worries
Beri-Beri	105	0.089	Beri-Beri
Betændelse i Haanden	196	0.166	Infection in the hand
Blodstigning til Hovedet	25	0.021	Blodrush to the head
Bronkit	196	0.166	Bronchitis
Brystbetændelse	22	0.019	Breast infection
Brystsygdom	51	0.043	Breast disease
Cholera	23	0.019	Cholera
Chorea	66	0.056	Spasms
COMPLETION	27	0.023	COMPLETION
Demens	1065	0.901	Dementia
Diabetes	32	0.027	Diabetes
Diarré	2	0.002	Diarrhoea
Diegivning	2137	1.809	Breastfeeding
Difteri	58	0.049	Diphtheria
Diverse Handicap	182	0.154	Miscellaneous disability
Drik	5822	4.927	Drinking
Endometriose	39	0.033	Endometriosis
Enterit	3	0.003	Enteritis
Epilepsi	2204	1.865	Epilepsy
Erysipelas	3	0.003	Skin infection
Fængselsophold	1625	1.375	Incarceration
Familiesorg	628	0.531	Familial issues
Fattigdom	1725	1.460	Poverty
Forbrænding	64	0.054	Burn
Fordøjelsesproblemer	908	0.768	Digestive issues/indigestion
Forfrysning	66	0.056	Freeze
Forgiftning	58	0.049	Poisoning
Forkølelse	1063	0.900	Common cold
Fraktur	207	0.175	Fracture
Fysisk Handicap	75	0.063	Physical disability
Fysisk Sygdom	3341	2.827	Physical disorder
Fysiske Smerter	33	0.028	Physical pain
Gastrit	173	0.146	Gastritis
Gastroenterit	62	0.052	Gastroenteritis
Gigtfeber	483	0.409	Rheumatic fever
Gjennemgaaet	30	0.025	Undergone (procedure)
Gonorrhoe	79	0.067	Gonorrhoea
Græmmelse	47	0.040	Disgust
Graviditet	1834	1.552	Pregnancy
Gul Feber	24	0.020	Yellow fever
Halssygdom	86	0.073	Throat disease

Table 3: The frequency is the total frequency (all occurrences) *(continued)*

Cause	Frequency	Percentage	EN
Herpes zoster	69	0.058	Herpes zoster
Hjernebetændelse	974	0.824	Brain infection
Hjernebetændelse i Barndommen	25	0.021	Brain infection during childhood
Hjernefejl	284	0.240	Brain problem
Hjernerystelse	3412	2.888	Concussion
Hjerneskallebrud	87	0.074	Cranial fracture
Hjernesvulst	144	0.122	Brain tumour
Hjernesygdom	291	0.246	Cerebral disease
Hjernesygdom i Barndommen	24	0.020	Cerebral disease during childhood
Hjertefejl	541	0.458	Heart problem
Hjertesygdom	111	0.094	Cardio-vascular disorder
Hørehæmmet	704	0.596	Hearing loss
Hovedpine	122	0.103	Headache
Hyperthyreose	270	0.228	Hypothyrodism
Hysteri	847	0.717	Hysteria
Infektionssygdom	914	0.774	Infectious disease
Inflammation	128	0.108	Inflammation
Influenza	1010	0.855	Influenza
Intoxikationer	173	0.146	Intoxication
Kærlighedssorg	1885	1.595	Heartbreak
Kikhoste	33	0.028	Whooping cough
Konflikter	306	0.259	Conflict
Kosanguinitet	82	0.069	Cosanguinity
Kræft	229	0.194	Cancer
Kusma	104	0.088	Mumps
Kyfose	40	0.034	Kyphosis
Læseri	99	0.084	Religious devotion
Lammelse	77	0.065	Paralysis
Leprosi	55	0.047	Leprosy
Livsstil	1151	0.974	Lifestyle
Lungebetændelse	755	0.639	Pneumonia or plurisy
Lupus	74	0.063	Lupus
Lymphangitis	65	0.055	Lymphangitis
Mæslinger	299	0.253	Measles
Malaria	181	0.153	Malaria
Marasmus	70	0.059	Malnutrition
Masturbation	3558	3.011	Masturbation
Mavesaar	109	0.092	Stomach ulcer
Mavesygdom	24	0.020	Stomach disorder
Menorrhagi	124	0.105	Menorrhagia
Menstruationsuordener	748	0.633	Menstrual problems
Mental Handicap	1867	1.580	Mental disability
Microcephali	57	0.048	Microcephaly
Militærtjeneste	269	0.228	Military
Misbrug af Chloral	73	0.062	Substance abuse of sedatives
Misbrug af Kaffe	24	0.020	Substance abuse of coffee
Misbrug af Merkur	21	0.018	Substance abuse of mercury
Misbrug af Morfin	135	0.114	Substance abuse of morphine
Misbrug af Tobak	71	0.060	Substance abuse of tobacco
Mishandling	182	0.154	Abuse
Mishandling under Opvækst	125	0.106	Abuse in childhood
MULTIPLE	1019	0.862	MULTIPLE
Nefrit	97	0.082	Nephritis
Nervechok	6264	5.301	Shock
Neuralgi	49	0.041	Neuralgia
Neurasteni	505	0.427	Neurastenia
Neurosyphilis	25	0.021	Neurosyphilis
Nyrebetændelse	40	0.034	Renal infection
Nyresygdom	512	0.433	Kidney disease
Observation	113	0.096	Observation
Øienbetændelse	62	0.052	Eye infection

Table 3: The frequency is the total frequency (all occurrences) *(continued)*

Cause	Frequency	Percentage	EN
Øiensygdom	106	0.090	Eye disease
Operation	960	0.812	Operation
Ophold i utlandet	165	0.140	Emigration
Ørebetændelse	219	0.185	Ear infection
Øresygdom	329	0.278	Ear disease
Osteomyelit	30	0.025	Osteomyelitis
OTHER	388	0.328	OTHER
Overanstrængelse	4737	4.009	Overexertion
Overgangsalder	751	0.636	Menopause
Parkinsons	23	0.019	Parkinsons
Pårørende	66	0.056	Relative
Pengespekulation	72	0.061	Financial worries
Phlegmone	26	0.022	Skin infection
Poliomyelit	104	0.088	Poliomyelitis
Politisk Ekstremisme	46	0.039	Political extremism
Prolaps	68	0.058	Prolapse
Psykisk Sygdom	335	0.284	Mental disease
Psykopati	163	0.138	Psychopathy
Pubertetsutvikling	68	0.058	Puberty
Puerperium	3785	3.203	Puerperal disease
Pyæmi	68	0.058	Blood infection
Rakit	644	0.545	Rickets
Religiøse Grublerier	1060	0.897	Religious brooding
Religiøse Indflydelser	1882	1.593	Religious influence
Rheumatisme	86	0.073	Rheumatic disease
Rosen	173	0.146	Skin infection
Rygmarvssygdom	115	0.097	Spinal bone marrow disorder
Rygsmerte	104	0.088	Back pain
Scabies	32	0.027	Scabies
Scoliose	25	0.021	Scoliosis
Seksualitet	1135	0.961	Sexuality
Sexarbejde	69	0.058	Sex work
Sindsindtryk	26	0.022	Mental images
Sindslidelser	1208	1.022	Mental suffering
Skarlagensfeber	209	0.177	Scarlet fever
Skinnebenssår	137	0.116	Shin splints
Slagtilfælde	1250	1.058	Stroke
Smittefrygt	21	0.018	Germophobia
Socialt Udsat	266	0.225	Socially vulnerable
Solstik	716	0.606	Sunstroke
Sorg	1609	1.362	Grief
Søvnløshed	1507	1.275	Insomnia
Sterilitet	24	0.020	Sterile
SUBTOTAL	279	0.236	SUBTOTAL
Svækkelse	1295	1.096	Weakness
Svulst paa Halsen	24	0.020	Tumour on the neck/throat
Sygdom	70	0.059	Disease
Synshæmmet	110	0.093	Visually impaired
Syphilidophobi	166	0.140	Syphilidophobia
Syphilis	3730	3.157	Syphilis
Talehæmmet	74	0.063	Speech impaired
Talrige Fødsler	52	0.044	Multiple births
Tidligere Sindssygdom	1135	0.961	Previous mental health issues
Tuberkulose	1164	0.985	Tuberculosis
Typhoidfeber	807	0.683	Typhoid fever
Udviklingshæmmet	1160	0.982	Developmental disorder
Ulykke	848	0.718	Accident
Underernæring	350	0.296	Malnourishment
Underlivssygdom	218	0.184	Pelvic disorder
UNKNOWN	1955	1.655	UNKNOWN
Urinorgansygdom	190	0.161	Urinary disorder

Table 3: The frequency is the total frequency (all occurrences) *(continued)*

Cause	Frequency	Percentage	EN
Uterinsygdom	434	0.367	Uterine disease
Vaadeskud	23	0.019	Unintentional discharge (gun not bodily fluid)
Vold	77	0.065	Violence
Voldtægtsforsøg	25	0.021	Rape

2.1 Harmonisation of causes for admissions

The harmonisation of causes is provided in Table 4 for reproducibility followed by Sankey diagrams coloured by information source (Figure 3, whose caption is uncharacteristically at the top due to being broken over multiple pages).

Table 1: Facilities classified by region

Facility	NUTS region	Region name
Bergen	NO0A	Vestlandet
Blakstad	NO08	Oslo og Viken
Bratsberg	NO08	Oslo og Viken
Dale	NO0A	Vestlandet
Dedichens	NO08	Oslo og Viken
Dikemark	NO08	Oslo og Viken
Eg	NO09	Agder og Sør-Østlandet
Faret	NO08	Oslo og Viken
Fastings Minde	NO0A	Vestlandet
Gaustad	NO08	Oslo og Viken
Kriminalasylet	NO06	Trøndelag
Kristiania	NO08	Oslo og Viken
Kristiansand	NO09	Agder og Sør-Østlandet
Lier	NO08	Oslo og Viken
Møllendal	NO0A	Vestlandet
Neevengården	NO0A	Vestlandet
Opdøl	NO0A	Vestlandet
Oslo	NO08	Oslo og Viken
Østmarken	NO06	Trøndelag
Prestsæter	NO02	Innlandet
Reitgjerdet	NO06	Trøndelag
Rønvik	NO07	Nord-Norge
Rosenbergs	NO0A	Vestlandet
Rosenbergs	NO0A	Vestlandet
Rotvold	NO06	Trøndelag
Sanderud	NO02	Innlandet
Stavanger	NO0A	Vestlandet
Trondhjem	NO06	Trøndelag
Valen	NO0A	Vestlandet
Veum	NO08	Oslo og Viken

Table 2: Bertillon's main classifications

Code	Description
I	General diseases
I-A	Epidemic diseases
I-B	Other general diseases
II	Diseases of nervous system and sense organs
III	Diseases of circulatory system
IV	Diseases of respiratory system
V	Diseases of digestive system
VI	Diseases of genito-urinary system
VII	Puerperal diseases
VIII	Diseases of skin and annexes
IX	Diseases of locomotor organs
X	Malformations
XI	Diseases of early infancy
XII	Diseases of old age
XIII	Effects of external causes
XIV	Ill-defined diseases

Table 4: Terms harmonised

Before: 1455 unique categories	After: 705 unique categories
8) Lungesvindsot 1 organisk Hjertesygdom	Tuberkulose, Hjertesygdom
Aarebetændelse	Årebetændelse
Aarsager er opgivet for	COMPLETION
Abort	Abort
Absces	Absces
Absces	Inflammation
Ængstelse for Operation	Angst
Ærgrelse	Ærgelse
Ærgrelse og Disposition efter tidligere Anfald	Ærgelse, Tidligere Sindssygdom
Afkræftelse	Afkræftelse
Afkræftelse ved Diarrhoe og Nyresygdom	Fordøjelsesproblemer, Nyresygdom
Akut Infektionssygdom	Infektionssygdom
Alderdom	Alderdom
Alderdom og Apoplexi	Alderdom, Slagtilfælde
Alderdom og Arv	Alderdom, Arv
Alderdom og Drik	Alderdom, Drik
Alderdom og Familiesorg, religiøse Indflydelser	Alderdom, Familiesorg, Religiøse Indflydelser
Alderdom og legemlig Svækkelse	Alderdom, Svækkelse
Alderdom og legemlig Sygdom	Alderdom, Fysisk Sygdom
Alderdom og Lues	Alderdom, Syphilis
Alderdom og Mangel	Alderdom, Fattigdom
Alderdom og Modgang	Alderdom, Fattigdom
Alderdom og Ondtliden	Alderdom, Forkølelse
Alderdom og Operation	Alderdom, Operation
Alderdom og Senilitet	Alderdom, Demens
Alderdom og Sindsindtryk	Alderdom, Nervechok
Alderdom og Stød paa Hovedet	Alderdom, Hjernerystelse
Alderdom og tidligere Sindssygdom	Alderdom, Tidligere Sindssygdom
Alderdom og Udsvævelser	Alderdom, Seksualitet
Alderdom, Tidligere Sindssygdom	Alderdom, Tidligere Sindssygdom
Alderdomssvækkelse	Alderdom
Alderdomssvækkelse og Søvnløshed	Alderdom, Søvnløshed
Alderdomssvaghed	Alderdom
All	ALL
Amerikaophold	Amerikaophold
Amerikaophold og Kjærestesorg	Amerikaophold, Kærlighedssorg
Anæmi	Anæmi
Anæmi og Blegsot	Anæmi
Anæmi og Hjertesygdom	Anæmi, Hjertesygdom

Table 4: Terms harmonised *(continued)*

Before: 1455 unique categories	After: 705 unique categories
Anæmi og Hysteri	Anæmi, Hysteri
Anæmi og Mavekatarr	Anæmi, Mavesaar
Anæmi og Nervesvækkelse	Anæmi, Svækkelse
Anæmi og Nervesvaghed	Anæmi, Svækkelse
Anæmi og Sindsindtryk	Anæmi, Sindsindtryk
Andre aarsaker	OTHER
Andre aarsaker	UNKNOWN
Andre Aarsaker	OTHER
Andre årsaker i denne gruppe: slett forhold til overordnede, ”slett” selskap, ensomt opholdssted, selvbe-breidelse	Socialt Udsat
Andre benbrud	Fraktur
Andre infeksjonssygdommer	Infektionssygdom
Andre infeksjonssykdommer	Infektionssygdom
Andre legemlige sygdom mer	Fysisk Sygdom
Andre legemlige sykdommer	Fysisk Sygdom
Andre ophold (Spitsbergen, Kristiania fengsel)	Fængselsophold
Andre psykiske årsaker	Psykisk Sygdom
Andre sygdommer	OTHER
Anemi	Anæmi
Anger	Ærgelse
Angina og Bronkit	Angina, Bronkit
Ankylose	Ankylose
Anomalier i sanseorganerne (blindhet, døvhet etc)	Fysisk Handicap
Ansigtsrosen	Rosen
Antornitoksikasjon	Forgiftning
Apopleksi	Slagtilfælde
Apoplexi	Slagtilfælde
Apoplexi, hjerneslag	Slagtilfælde
Apoplexi; hjerneslag	Slagtilfælde
Appendicit	Appendicit
Arbeidsskyhet	Overanstrængelse
Arrest	Fængselsophold
Arteiiosklerose	Arteriosklerose
Arteriosclerose	Arteriosklerose
Arteriosklerose	Arteriosklerose
Arteriosklerose og Ateromasi	Arteriosklerose, Atheromasi
Arv	Arv
Arv og Abscessus abdominalis	Arv, Mavesaar
Arv og Alderdom	Arv, Alderdom

Table 4: Terms harmonised *(continued)*

Before: 1455 unique categories	After: 705 unique categories
Arv og Amerikaophold	Arv, Amerikaophold
Arv og Anæmi	Arv, Anæmi
Arv og Asthma	Arv, Asthma
Arv og Barselfeber	Arv, Puerperium
Arv og Barselseng	Arv, Puerperium
Arv og Blodtab	Arv, Anæmi
Arv og Bronkit	Arv, Bronkit
Arv og Cephalalg	Arv, Hovedpine
Arv og Cirrhosis hepatis	Arv, Nyresygdom
Arv og Drik	Arv, Drik
Arv og Dysmenorrhoe	Arv, Menorrhagi
Arv og Dyspepsi	Arv, Fordøjelsesproblemer
Arv og Enterit	Arv, Enterit
Arv og Epilepsi	Arv, Epilepsi
Arv og Erysipelas	Arv, Erysipelas
Arv og Febersygdom	Arv, Infektionssygdom
Arv og Fraktur	Arv, Fraktur
Arv og Furunkulose	Arv, Inflammation
Arv og Gastro-enterit	Arv, Gastroenterit
Arv og Gonorré og Sindsindtryk	Arv, Gonorrhoe, Sindsindtryk
Arv og Graviditet	Arv, Graviditet
Arv og Hjernebetændelse	Arv, Hjernebetændelse
Arv og Hjernebetændelse i Barnealderen	Arv, Hjernebetændelse i Barndommen
Arv og Hjernekongestion	Arv, Slagtilfælde
Arv og Hjertesygdom	Arv, Hjertesygdom
Arv og Hysteri	Arv, Hysteri
Arv og Hysteri og Influenza	Arv, Hysteri, Influenza
Arv og Infektion	Arv, Infektionssygdom
Arv og Influenza	Arv, Influenza
Arv og Influenza og Sindsindtryk	Arv, Influenza, Sindsindtryk
Arv og Klimakterium	Arv, Overgangsalder
Arv og Klorose	Arv, Anæmi
Arv og langvarig Diegivning	Arv, Diegivning
Arv og legemlig Svækkelse	Arv, Svækkelse
Arv og legemlig Sygdom	Arv, Fysisk Sygdom
Arv og Masturbation	Arv, Masturbation
Arv og Næringssorger	Arv, Fattigdom
Arv og Nattevaagen	Arv, Søvnløshed
Arv og Nefrit	Arv, Nefrit

Table 4: Terms harmonised *(continued)*

Before: 1455 unique categories	After: 705 unique categories
Arv og Nervefeber	Arv, Nervechok
Arv og Nervesvækkelse og Sindsindtryk	Arv, Svækkelse, Sindsindtryk
Arv og Nervøsitet	Arv, Angst
Arv og Neurastheni	Arv, Neurasteni
Arv og Nyresygdom	Arv, Nyresygdom
Arv og Ondtliden	Arv, Forkølelse
Arv og Overanstrængelse	Arv, Overanstrængelse
Arv og Pengespekulation	Arv, Pengespekulation
Arv og Pleurit	Arv, Lungebetændelse
Arv og Pneumoni	Arv, Lungebetændelse
Arv og Puerperal Parametrit	Arv, Puerperium
Arv og religiøs Paavirkning	Arv, Religiøse Indflydelser
Arv og Rheum Acut	Arv, Rheumatisme
Arv og Rheumatismus acutus	Arv, Rheumatisme
Arv og Sindsindtryk	Arv, Nervechok
Arv og Sindslidelse	Arv, Sindslidelser
Arv og Skræk	Arv, Angst
Arv og Sorg	Arv, Sorg
Arv og Stød paa Hovedet	Arv, Hjernerystelse
Arv og Svækkelsestilstande	Arv, Svækkelse
Arv og Svangerskab	Arv, Graviditet
Arv og Syfilis	Arv, Syphilis
Arv og Syphilis	Arv, Syphilis
Arv og Tandbyld	Arv, Inflammation
Arv og tidligere Sindssygdom	Arv, Tidligere Sindssygdom
Arv og Tuberculosis testis	Arv, Tuberkulose
Arv og Tuberkulose	Arv, Tuberkulose
Arv og Uterussygdom med Blodtab	Arv, Uterinsygdom, Anæmi
Arv og Vulneration af Øiet med paafølgende Enukleation	Arv, Operation
Arv, Barselseng og leg Sygdom	Arv, Puerperium, Fysisk Sygdom
Arv, Barselseng og Sindsindtryk	Arv, Puerperium, Nervechok
Arv, Drik og Fængselsstraf	Arv, Drik, Fængselsophold
Arv, Drik og leg Sygdom	Arv, Drik, Fysisk Sygdom
Arv, Drik og legemlig Sygdom	Arv, Drik, Fysisk Sygdom
Arv, Drik og Stød paa Hovedet	Arv, Drik, Hjernerystelse
Arv, Drik og Syphilis	Arv, Drik, Syphilis
Arv, Drik, legemlig Sygdom og Stød paa Hovedet	Arv, Drik, Fysisk Sygdom, Hjernerystelse
Arv, legemlig Sygdom og Sindsindtryk	Arv, Fysisk Sygdom, Nervechok
Arv, legemlig Sygdom og Stød paa Hovedet	Arv, Fysisk Sygdom, Hjernerystelse

Table 4: Terms harmonised *(continued)*

Before: 1455 unique categories	After: 705 unique categories
Arv, Masturbation og Sindsindtryk	Arv, Masturbation, Nervechok
Arv, Overanstrængelse og Anæmi	Arv, Overanstrængelse, Anæmi
Arv, Overanstrængelse og Nattevaagen	Arv, Overanstrængelse, Søvnløshed
Arv, Overanstrængelse og religiøs Paavirkning	Arv, Overanstrængelse, Religiøse Indflydelser
Arv, Overanstrængelse og Sindsindtryk	Arv, Overanstrængelse, Nervechok
Arv, Sindsindtryk og langvarig Diegivning	Arv, Nervechok, Diegivning
Arv, Sindsindtryk og legemlig Sygdom	Arv, Nervechok, Fysisk Sygdom
Arv, Sindsindtryk og Ondtliden	Arv, Nervechok, Forkølelse
Arv, Sindsindtryk og Overanstrængelse	Arv, Nervechok, Overanstrængelse
Arv, Sindsindtryk og Stød paa Hovedet	Arv, Nervechok, Hjernerystelse
Arv, Sindsindtryk og Syphilis	Arv, Nervechok, Syphilis
Arv, Stød paa Hovedet og Drik	Arv, Hjernerystelse, Drik
Arv, tidligere Sindssygdom og Sindsindtryk	Arv, Tidligere Sindssygdom, Nervechok
Arvelighed i Forbindelse med andre Aarsager	Arv
arveligt Anlæg	Arv
Arveligt Anlæg	Arv
Arveligt Anlæg i Forbindelse med andre Aarsager som smertelige Sindsindtryk, Drik, Selvbesmittelse	Arv, Nervechok, Drik, Masturbation
Arveligt Anlæg i Forbindelse med andre Aarsager som smertelige Sindsindtryk, Selvbesmittelse, Abort	Arv, Nervechok, Masturbation, Abort
Arveligt Anlæg i Forbindelse med andre Aarsager som smertelige Sindsindtryk, Selvbesmittelse, Gigt-feber, Menstruationsuordener, Ondtliden	Arv, Nervechok, Masturbation, Gigtfeber, Menstruationsuordener, Forkølelse
Arveligt Anlæg i Forbindelse med andre Aarsager som smertelige Sindsindtryk, Selvbesmittelse, Ondtli-den	Arv, Nervechok, Masturbation, Forkølelse
Arveligt Anlæg i Forbindelse med andre Aarsager, saasom smertelige Sindsindtryk, Drik, Selvbesmittelse, Barselseng etc etc	Arv, Nervechok, Drik, Masturbation, Puerperium
Arveligt Anlæg i Forbindelse med andre Aarsager, saasom smertelige Sindsindtryk, Drik, Selvbesmittelse, Barselseng Menstruationsuordener etc etc	Arv, Nervechok, Drik, Masturbation, Puerperium, Menstruationsuordener
Arveligt Anlæg i Forbindelse med en af de opførte Aarsager	Arv
Arveligt Anlæg i Forbindelse med legemlig svækkende Aarsager som Sygdom og Selvbesmittelse	Arv, Sygdom, Masturbation
Arveligt Anlæg i Forbindelse med legemlig svækkende Aarsager, som Sygdom og Selvbesmittelse	Arv, Sygdom, Masturbation
Arveligt Anlæg i Forbindelse med legemligt svækkende Aarsager som Sygdom, Selvbesmittelse	Arv, Svækkelse
Arveligt Anlæg i Forbindelse med legemligt svækkende Aarsager som Sygdom, Selvbesmittelse, Over-anstrængelse	Arv, Svækkelse
Arveligt Anlæg i Forbindelse med Sindsindtryk og religiøse Indflydelser	Arv, Nervechok, Religiøse Indflydelser
Arveligt Anlæg og Mæslinger	Arv, Mæslinger
Arveligt Anlæg og aandelig Overanstrengelse	Arv, Overanstrængelse
Arveligt Anlæg og Abort	Arv, Abort
Arveligt Anlæg og Abscessus abdominis	Arv, Mavesaar
Arveligt Anlæg og Afkræftelse	Arv, Afkræftelse
Arveligt Anlæg og Alderdom	Arv, Alderdom

Table 4: Terms harmonised *(continued)*

Before: 1455 unique categories	After: 705 unique categories
Arveligt Anlæg og Amerikaophold	Arv, Amerikaophold
Arveligt Anlæg og Anæmi og Sindsindtryk	Arv, Anæmi, Sindsindtryk
Arveligt Anlæg og Anæmi, almindelig Svækkelse, Influenza, Lungebetændelse, Nervefeber, Spondylarthrocace	Arv, Anæmi, Svækkelse, Influenza, Lungebetændelse, Nervechok, Rygsmerte
Arveligt Anlæg og anden legemlig Sygdom	Arv, Fysisk Sygdom
Arveligt Anlæg og Apoplexi	Arv, Slagtilfælde
Arveligt Anlæg og Ascaris	Arv, Ascaris
Arveligt Anlæg og Asthma	Arv, Asthma
Arveligt Anlæg og Astma og Sindsindtryk	Arv, Asthma, Sindsindtryk
Arveligt Anlæg og Barselseng	Arv, Puerperium
Arveligt Anlæg og Barselseng, langvarig Diegivning	Arv, Puerperium, Diegivning
Arveligt Anlæg og Beskadigelse (Hugsaar i Læggen)	Arv, Ulykke
Arveligt Anlæg og Besvangring	Arv, Graviditet
Arveligt Anlæg og Chorea	Arv, Chorea
Arveligt Anlæg og Degeneration	Arv, Mental Handicap
Arveligt Anlæg og Diegivning	Arv, Diegivning
Arveligt Anlæg og Difteri	Arv, Difteri
Arveligt Anlæg og Digestionsuordener	Arv, Fordøjelsesproblemer
Arveligt Anlæg og Døvhed	Arv, Hørehæmmet
Arveligt Anlæg og Drik	Arv, Drik
Arveligt Anlæg og Drik og Stød paa Hovedet	Arv, Drik, Hjernerystelse
Arveligt Anlæg og Dyspepsi	Arv, Fordøjelsesproblemer
Arveligt Anlæg og Dyspepsi og Sindsindtryk	Arv, Fordøjelsesproblemer, Sindsindtryk
Arveligt Anlæg og Eksercitie	Arv, Militærtjeneste
Arveligt Anlæg og Epilepsi	Arv, Epilepsi
Arveligt Anlæg og Fængselsstraf	Arv, Fængselsophold
Arveligt Anlæg og Febersygdom	Arv, Infektionssygdom
Arveligt Anlæg og forudgaaende Sindssygdom	Arv, Tidligere Sindssygdom
Arveligt Anlæg og Hjernebetændelse	Arv, Hjernebetændelse
Arveligt Anlæg og Hjerneslag	Arv, Slagtilfælde
Arveligt Anlæg og Hjernesvulst	Arv, Hjernesvulst
Arveligt Anlæg og Hjertefeil	Arv, Hjertefejl
Arveligt Anlæg og Hjertesygdom	Arv, Hjertesygdom
Arveligt Anlæg og huslige Bekymringer	Arv, Fattigdom
Arveligt Anlæg og Hysteri	Arv, Hysteri
Arveligt Anlæg og Imbecillitet	Arv, Mental Handicap
Arveligt Anlæg og Influenza	Arv, Influenza
Arveligt Anlæg og Influenza og Abort	Arv, Influenza, Abort
Arveligt Anlæg og Influenza og Sindsindtryk	Arv, Influenza, Sindsindtryk

Table 4: Terms harmonised *(continued)*

Before: 1455 unique categories	After: 705 unique categories
Arveligt Anlæg og Kjærlighedssorg	Arv, Kærlighedssorg
Arveligt Anlæg og kjønslige Udskeielser	Arv, Livsstil
Arveligt Anlæg og Klimakterium	Arv, Overgangsalder
Arveligt Anlæg og Klorose	Arv, Anæmi
Arveligt Anlæg og legemlig Svækkelse	Arv, Svækkelse
Arveligt Anlæg og legemlig Svækkelse og Sindsindtryk	Arv, Svækkelse, Sindsindtryk
Arveligt Anlæg og legemlig svækkende Aarsager	Arv, Svækkelse
Arveligt Anlæg og legemlig Svaghed	Arv, Svækkelse
Arveligt Anlæg og legemlig Sygdom	Arv, Fysisk Sygdom
Arveligt Anlæg og legemlig Sygdom 1)	Arv, Fysisk Sygdom
Arveligt Anlæg og Lungebetændelse	Arv, Lungebetændelse
Arveligt Anlæg og Mæslinger	Arv, Mæslinger
Arveligt Anlæg og Masturbation	Arv, Masturbation
Arveligt Anlæg og Menstruationsuordener	Arv, Menstruationsuordener
Arveligt Anlæg og Metrorrhagi	Arv, Menorrhagi
Arveligt Anlæg og Næringssorg	Arv, Fattigdom
Arveligt Anlæg og Nattevaagen	Arv, Søvnløshed
Arveligt Anlæg og Nervesygdom	Arv, Nervechok
Arveligt Anlæg og Neuralgi	Arv, Neuralgi
Arveligt Anlæg og Nyresygdom	Arv, Nyresygdom
Arveligt Anlæg og og flere Svangerskaber	Arv, Talrige Fødsler
Arveligt Anlæg og Ondtliden	Arv, Forkølelse
Arveligt Anlæg og Øresygdom	Arv, Øresygdom
Arveligt Anlæg og Overanstrængelse	Arv, Overanstrængelse
Arveligt Anlæg og Pengespekulationer	Arv, Pengespekulation
Arveligt Anlæg og Pneumoni	Arv, Lungebetændelse
Arveligt Anlæg og religiøs Indflydelse	Arv, Religiøse Indflydelser
Arveligt Anlæg og religiøs Paavirkning	Arv, Religiøse Indflydelser
Arveligt Anlæg og religiøse Indflydeiser	Arv, Religiøse Indflydelser
Arveligt Anlæg og religiøse Indflydelser	Arv, Religiøse Indflydelser
Arveligt Anlæg og religiøse Skrupler	Arv, Religiøse Grublerier
Arveligt Anlæg og Rosen	Arv, Rosen
Arveligt Anlæg og Selvbesmittelse	Arv, Masturbation
Arveligt Anlæg og Selvbesrnittelse	Arv, Masturbation
Arveligt Anlæg og Senescens	Arv, Demens
Arveligt Anlæg og sexuelle Indflydelser (Selvbesmittelse, Impotents)	Arv, Seksualitet
Arveligt Anlæg og Sindsindtryk	Arv, Nervechok
Arveligt Anlæg og Sindsindtryk, religiøse Indflydelser	Arv, Nervechok, Religiøse Indflydelser
Arveligt Anlæg og Sindslidelser	Arv, Sindslidelser

Table 4: Terms harmonised *(continued)*

Before: 1455 unique categories	After: 705 unique categories
Arveligt Anlæg og Skarlagensfeber	Arv, Skarlagensfeber
Arveligt Anlæg og Skræk	Arv, Angst
Arveligt Anlæg og slet Behandling	Arv, Mishandling
Arveligt Anlæg og slet Opdragelse	Arv, Socialt Udsat
Arveligt Anlæg og smertelige Sindsindtryk	Arv, Nervechok
Arveligt Anlæg og smertelige Sindsindtryk (Kjærlighedssorg, økonomiske Bekymringer, religiøse Indfly-delser etc)	Arv, Nervechok
Arveligt Anlæg og smertelige Sindsindtryk (som ulykkeligeFamilieforhold, økonom Bekymringer, Kjærlighedssorg osv)	Arv, Nervechok
Arveligt Anlæg og smerteligt Sindsindtryk	Arv, Nervechok
Arveligt Anlæg og Solstik	Arv, Solstik
Arveligt Anlæg og Sorg	Arv, Sorg
Arveligt Anlæg og Søvnløshed	Arv, Søvnløshed
Arveligt Anlæg og Stod paa Hovedet	Arv, Hjernerystelse
Arveligt Anlæg og Stød paa Hovedet	Arv, Hjernerystelse
Arveligt Anlæg og Svækkelsestilstand	Arv, Svækkelse
Arveligt Anlæg og Svangerskab	Arv, Graviditet
Arveligt Anlæg og Svangerskab, Barselseng, Diegivning	Arv, Graviditet, Puerperium, Diegivning
Arveligt Anlæg og Syfilis	Arv, Syphilis
Arveligt Anlæg og Syphilis	Arv, Syphilis
Arveligt Anlæg og tidligere Anfald	Arv, Tidligere Sindssygdom
Arveligt Anlæg og tidligere Sindssygdom	Arv, Tidligere Sindssygdom
Arveligt Anlæg og Trauma capitis	Arv, Hjernerystelse
Arveligt Anlæg og Trauma Capitis	Arv, Hjernerystelse
Arveligt Anlæg og Tuberculose	Arv, Tuberkulose
Arveligt Anlæg og Tyfoidfeber og Influenza	Arv, Typhoidfeber, Influenza
Arveligt Anlæg og Tyfoidfeber og Pleurit	Arv, Typhoidfeber, Lungebetændelse
Arveligt Anlæg og Tyfus	Arv, Typhoidfeber
Arveligt Anlæg og Typhoidfeber	Arv, Typhoidfeber
Arveligt Anlæg og Udsvævelse	Arv, Seksualitet
Arveligt Anlæg og uheldige Familieforhold	Arv, Familiesorg
Arveligt Anlæg og ulykkeligt Ægteskab	Arv, Kærlighedssorg
Arveligt Anlæg og uordentlig Levnet	Arv, Livsstil
Arveligt Anlæg og uordentligt Liv	Arv, Livsstil
Arveligt Anlæg og Værkefinger	Arv, Betændelse i Haanden
Arveligt Anlæg, legemlig Sygdom og Sindsindtryk	Arv, Fysisk Sygdom, Nervechok
Arveligt Anlæg, Anæmi og Sindsindtryk	Arv, Anæmi, Sindsindtryk
Arveligt Anlæg, anden legemlig Sygdom og Sindsindtryk	Arv, Fysisk Sygdom, Nervechok
Arveligt Anlæg, Barselseng og Nattevaag	Arv, Puerperium, Søvnløshed

Table 4: Terms harmonised *(continued)*

Before: 1455 unique categories	After: 705 unique categories
Arveligt Anlæg, Barselseng og Sorg	Arv, Puerperium, Sorg
Arveligt Anlæg, Barselseng, Afkræftelse og Sorg	Arv, Puerperium, Afkræftelse, Sorg
Arveligt Anlæg, Drik og Sindsindtryk	Arv, Drik, Nervechok
Arveligt Anlæg, Drik og Epilepsi	Arv, Drik, Epilepsi
Arveligt Anlæg, Drik og Fængselsstraf	Arv, Drik, Fængselsophold
Arveligt Anlæg, Drik og Forfrysning	Arv, Drik, Forfrysning
Arveligt Anlæg, Drik og Kronisk Gastrit	Arv, Drik, Gastrit
Arveligt Anlæg, Drik og legemlig Sygdom	Arv, Drik, Fysisk Sygdom
Arveligt Anlæg, Drik og Masturbation	Arv, Drik, Masturbation
Arveligt Anlæg, Drik og Nyresygdom	Arv, Drik, Nyresygdom
Arveligt Anlæg, Drik og Selvbesmittelse	Arv, Drik, Masturbation
Arveligt Anlæg, Drik og Stød paa Hovedet	Arv, Drik, Hjernerystelse
Arveligt Anlæg, Drik og Syfilis	Arv, Drik, Syphilis
Arveligt Anlæg, Drik og tidligere Sindssygdom	Arv, Drik, Tidligere Sindssygdom
Arveligt Anlæg, Epilepsi og Sindsindtryk	Arv, Epilepsi, Sindsindtryk
Arveligt Anlæg, legemlig svækkende Aarsager og Sindsindtryk	Arv, Svækkelse, Nervechok
Arveligt Anlæg, legemlig Svaghed og Sindsindtryk	Arv, Svækkelse, Nervechok
Arveligt Anlæg, legemlig Sygdom og foregaaende Sindssygdom	Arv, Fysisk Sygdom, Tidligere Sindssygdom
Arveligt Anlæg, legemlig Sygdom og Overanstrængelse	Arv, Fysisk Sygdom, Overanstrængelse
Arveligt Anlæg, legemlig Sygdom og religiøs Inflydelse	Arv, Fysisk Sygdom, Religiøse Indflydelser
Arveligt Anlæg, legemlig Sygdom og Sindsindtryk	Arv, Fysisk Sygdom, Nervechok
Arveligt Anlæg, legemlig Sygdom og Sindsindtryk, religiøse Indflydelser	Arv, Fysisk Sygdom, Nervechok, Religiøse Indflydelser
Arveligt Anlæg, legemlig Sygdom og Sindslidelser	Arv, Fysisk Sygdom, Sindslidelser
Arveligt Anlæg, legemlig Sygdom og Stød paa Hovedet	Arv, Fysisk Sygdom, Hjernerystelse
Arveligt Anlæg, legemlig Sygdom, Sindsindtryk og Imbecillitet	Arv, Fysisk Sygdom, Nervechok, Mental Handicap
Arveligt Anlæg, Overanstrængelse og huslige Sorger	Arv, Overanstrængelse, Fattigdom
Arveligt Anlæg, Overanstrængelse og legemlig Sygdom	Arv, Overanstrængelse, Fysisk Sygdom
Arveligt Anlæg, Overanstrængelse og Sindsindtryk	Arv, Overanstrængelse, Nervechok
Arveligt Anlæg, Sindsindtryk og Barselseng	Arv, Nervechok, Puerperium
Arveligt Anlæg, Sindsindtryk og foregaaende Sindssygdom	Arv, Nervechok, Tidligere Sindssygdom
Arveligt Anlæg, Sindsindtryk og Kjærlighedssorg	Arv, Nervechok, Kærlighedssorg
Arveligt Anlæg, Sindsindtryk og legeml Sygd	Arv, Nervechok, Fysisk Sygdom
Arveligt Anlæg, Sindslidelser, Kjærlighedssorg, religiøs Paavirkning	Arv, Sindslidelser, Kærlighedssorg, Religiøse Indflydelser
Arveligt Anlæg, Sindslidelser, smertelige Sindsindtryk, Kjærlig-hedssorg, huslige Bekymringer etc	Arv, Sindslidelser, Nervechok, Kærlighedssorg, Fattigdom
Arveligt Anlæg, Sindslidelser, smertelige Sindsindtryk, Kjærlighedssorg, aandelig Anstrængelse	Arv, Sindslidelser, Nervechok, Kærlighedssorg, Anstrængelse
Arveligt Anlæg, Sindslidelser, smertelige Sindsindtryk, Kjærlighedssorg, religiøs Paavirkning, ulykkelige Familieforhold, økonomisk Bekymring	Arv, Sindslidelser, Nervechok, Kærlighedssorg, Religiøse Indflydelser, Familiesorg, Fattigdom
Arveligt Anlæg, Sindslidelser, smertelige Sindsindtryk, Kjærlighedssorg, ulykkelige Familieforhold, aan-delig Anstrængelse	Arv, Sindslidelser, Nervechok, Kærlighedssorg, Familiesorg, Anstrængelse

Table 4: Terms harmonised *(continued)*

Before: 1455 unique categories	After: 705 unique categories
Arveligt Anlæg, smertelige Sindsindtryk, Sorger og Bekymringer	Arv, Nervechok, Sorg, Bekymringer
Arveligt Anlæg, Sorg og Bekymring	Arv, Sorg, Bekymringer
Arveligt Anlæg, Sorg og Bekymringer	Arv, Sorg, Bekymringer
Arveligt Anlæg, Stød paa Hovedet og Sorg	Arv, Hjernerystelse, Sorg
Arveligt Anlæg, Svangerskab og forudgaaende Sindssygdom	Arv, Graviditet, Tidligere Sindssygdom
Arveligt Anlæg, Svangerskab, Barselsseng, langvarig Diegivning	Arv, Graviditet, Puerperium, Diegivning
Arveligt Anlæg, Syphilis og Drik	Arv, Syphilis, Drik
Arveligt Anlæg, Udsvævelse og Barselseng	Arv, Seksualitet, Puerperium
Arveligt Anlæg, Udsvævelse og Drik	Arv, Seksualitet, Drik
Arveligt Anlæg, Udsvævelse og Selvbesmittelse	Arv, Seksualitet, Masturbation
Arveligt Anlæg, Udsvævelse og Skræk	Arv, Seksualitet, Angst
Arveligt Anlæg, Udsvævelse og Stød paa Hovedet	Arv, Seksualitet, Hjernerystelse
Arveligt Anlæg, Udsvævelse og ulykkelig Kjærlighed	Arv, Seksualitet, Kærlighedssorg
Arveligt Anlæg, Udsvævelse, Barselsseng og Diegivning	Arv, Seksualitet, Puerperium, Diegivning
Arveligt Anlæg, Udsvævelse, Selvbesmittelse og Overanstrængelse	Arv, Seksualitet, Masturbation, Overanstrængelse
Arveligt Anlæg, Udsvævelse, Sorg over afdødt Barn og Religionsskrupler	Arv, Seksualitet, Sorg, Religiøse Grublerier
Arveligt Anlæg:	Arv
Asthma	Asthma
Astma	Asthma
Ateromasi	Atheromasi
Atheroma art cerebri	Atheromasi
Atheromaci, Fedthjerte og daarlig Ernæring	Atheromasi, Underernæring
Atheromasi	Atheromasi
Atheromasi, Fedthjerte og daarlig Ernæring	Atheromasi, Underernæring
Atrophia cerebri	Demens
Barselfeber	Puerperium
Barselseng	Puerperium
Barselseng Diegivning	Puerperium, Diegivning
Barselseng og Abort	Puerperium, Abort
Barselseng og Aborter	Puerperium, Abort
Barselseng og Barselfeber	Puerperium
Barselseng og Blodmangel	Puerperium, Anæmi
Barselseng og diegivning	Puerperium, Diegivning
Barselseng og Diegivning	Puerperium, Diegivning
Barselseng og Familietvist	Puerperium, Familiesorg
Barselseng og Febersygdom	Puerperium, Infektionssygdom
Barselseng og langvarig Diegivning	Puerperium, Diegivning
Barselseng og legemlig Sygdom	Puerperium, Fysisk Sygdom
Barselseng og Mastit	Puerperium, Diegivning

Table 4: Terms harmonised *(continued)*

Before: 1455 unique categories	After: 705 unique categories
Barselseng og Nervefeber	Puerperium, Typhoidfeber
Barselseng og Nød	Puerperium, Fattigdom
Barselseng og Onani	Puerperium, Masturbation
Barselseng og Overanstrængelse	Puerperium, Overanstrængelse
Barselseng og Overanstrængelse, Afkræftelse	Puerperium, Overanstrængelse, Afkræftelse
Barselseng og religiøse Skrupler	Puerperium, Religiøse Grublerier
Barselseng og Sindsindtryk	Puerperium, Nervechok
Barselseng og Sindslidelser	Puerperium, Sindslidelser
Barselseng og Skuffelse	Puerperium, Ærgelse
Barselseng og smertelige Indtryk	Puerperium, Nervechok
Barselseng og smertelige Sindsindtryk	Puerperium, Nervechok
Barselseng og Sorg	Puerperium, Sorg
Barselseng og Svangerskab	Puerperium, Graviditet
Barselseng og tidligere Sindssygdom	Puerperium, Tidligere Sindssygdom
Barselseng og ulykkeligt Ægteskab	Puerperium, Kærlighedssorg
Barselseng, Abort	Puerperium, Abort
Barselseng, Diegivning, religiøse Indflydelser	Puerperium, Diegivning, Religiøse Indflydelser
Barselseng, langvarig Diegivning	Puerperium, Diegivning
Barselseng, legemlig Sygdom (Flebit) og Sindsindtryk	Puerperium, Årebetændelse, Nervechok
Barselseng, Svangerskab og Diegivning	Puerperium, Graviditet, Diegivning
Barselseng, Svangerskab og Sindslidelser	Puerperium, Graviditet, Sindslidelser
Basedows sygdom	Hyperthyreose
Bekymringer	Bekymringer
Benbrud	Fraktur
Beri-beri	Beri-Beri
Beri-Beri	Beri-Beri
Beruselse	Intoxikationer
Beruselse, Stød paa Hovedet, Samvittighedsnag	Intoxikationer, Hjernerystelse, Ærgelse
Beslægtede forældre	Kosanguinitet
Beslegtede forældre	Kosanguinitet
Besværlig tangforløsning	Diverse Handicap
Besvangring	Graviditet
Besvangring (udenfor Ægteskab)	Graviditet
Betændelse i Haanden (?)	Betændelse i Haanden
Biindfødt	Synshæmmet
Blærekatarrh	Urinorgansygdom
Blegsot	Anæmi
Blegsot og aandelig Overanstrængelse	Anæmi, Overanstrængelse
Blegsot og Anæmi	Anæmi

Table 4: Terms harmonised *(continued)*

Before: 1455 unique categories	After: 705 unique categories
Blegsot og Anæmi, smertelige Sindsindtryk	Anæmi, Nervechok
Blegsot og Blodfattigdom	Anæmi
Blegsot og Selvbesmittelse	Anæmi, Masturbation
Blegsot og smertelige Sindsindtryk	Anæmi, Nervechok
Blegsot, Anæmi	Anæmi
Blegsot, Blodfattigdom	Anæmi
Blodmangel	Anæmi
Blodmangel og Læseri	Anæmi, Læseri
Blodmangel, Blegsot	Anæmi
Blødning	Ulykke
Blødning (Metrorrhagi)	Menorrhagi
Blodtab	Anæmi
Blodtab (Aareladninger)	Anæmi
Blodtab, chronisk Diarrhoe	Anæmi, Fordøjelsesproblemer
Blyforgiftning	Forgiftning
Bronkit	Bronkit
Brystbetændelse	Brystbetændelse
Brystsygdom	Brystsygdom
Calorisk Indvirkning	Fordøjelsesproblemer
Cancer	Kræft
Cancer uteri	Kræft
Cerebrospinalmeningit	Hjernebetændelse
Chalorisk Indvirkning	Fordøjelsesproblemer
Chirurgisk Operation	Operation
Chirurgisk Sygdom i Underextremiteterne	Operation
Chloralmisbrug	Misbrug af Chloral
Chloroformnarkose	Misbrug af Chloral
Chlorose	Anæmi
Chlorose og anæmi	Anæmi
Chok	Nervechok
Cholera	Cholera
Chorea	Chorea
Contusion ved Ulykkestilfælde	Ulykke
Coxit	Tuberkulose
Cystit	Urinorgansygdom
Daarlig ernæring	Underernæring
Daarlige livsvilkaar	Socialt Udsat
Dårlige livsvilkår	Socialt Udsat
De til Observation indlagte	Observation

Table 4: Terms harmonised *(continued)*

Before: 1455 unique categories	After: 705 unique categories
Debilitet	Diverse Handicap
Debilitet	Mental Handicap
Degenerasjon	Mental Handicap
Degeneration	Mental Handicap
Degeneration og andre Svækkelsestilstande	Mental Handicap, Svækkelse
Degeneration og Drik	Mental Handicap, Drik
Degeneration og Hysteri	Mental Handicap, Hysteri
Degeneration og legemlig Sygdom (Rakit)	Mental Handicap, Rakit
Degeneration og Rakit	Mental Handicap, Rakit
Degeneration og Svækkelsestilstande	Mental Handicap, Svækkelse
Degeneration, Drik og Epilepsi	Mental Handicap, Drik, Epilepsi
Demens	Demens
Dements	Demens
Diabetes	Diabetes
Diarré	Diarré
Diegivning	Diegivning
Diegivning og Afkræftelse	Diegivning, Afkræftelse
Diegivning og Næringssorg	Diegivning, Fattigdom
Diegivning og Overanstrængelse	Diegivning, Overanstrængelse
Diegivning og religiøse Indflydelser	Diegivning, Religiøse Indflydelser
Diegivning og Sorg	Diegivning, Sorg
Diegivning og ulykkelige Familieforhold	Diegivning, Familiesorg
Diegivning, langvarig	Diegivning
Diegivning, smertelige Sindsindtryk	Diegivning, Nervechok
Diegivning, tildels med smertelige Sindsindtryk	Diegivning, Nervechok
Difteri	Difteri
Digestionmordener	Fordøjelsesproblemer
Digestionsuordener	Fordøjelsesproblemer
Diphtheritis	Difteri
direkte Arv	Arv
Disposition efter tidligere Anfald	Tidligere Sindssygdom
Disposition Nervøs konstitution	Angst
Disposition, erhvervet	Angst
Disposition, nervøs konstitution	Angst
Disposition, nervøsitet etc	Angst
Disposition, Nervøsitet etc	Angst
Døvhed	Hørehæmmet
Døvhet	Hørehæmmet
Døvhet og øresygdom	Hørehæmmet

Table 4: Terms harmonised *(continued)*

Before: 1455 unique categories	After: 705 unique categories
Døvstum	Hørehæmmet
Døvstumhet	Hørehæmmet
Døvstumhet, tunghorthet	Hørehæmmet
Døvstumhet, tunghørthet	Hørehæmmet
Drik	Drik
Drik hos Forældre	Pårørende
Drik i Forbindelse med andre Aarsager, saasom smertelige Sindsindtryk, Nervesmerter	Drik, Nervechok
Drik i Forbindelse med andre Aarsager, saasom smertelige Sindsindtryk, umoralsk Vandel, Slag paa Hovedet	Drik, Nervechok, Livsstil, Vold
Drik og aandelig Overanstrængelse	Drik, Overanstrængelse
Drik og akut Sykdom	Drik, Fysisk Sygdom
Drik og Arv	Drik, Arv
Drik og Ateromasi	Drik, Atheromasi
Drik og Bekymringer	Drik, Bekymringer
Drik og Epilepsi	Drik, Epilepsi
Drik og Fængsel	Drik, Fængselsophold
Drik og Fængselsstraf	Drik, Fængselsophold
Drik og Hjertefeil	Drik, Hjertefejl
Drik og Influenza	Drik, Influenza
Drik og legemlig Overanstrængelse	Drik, Overanstrængelse
Drik og legemlig Sygdom	Drik, Fysisk Sygdom
Drik og Lues	Drik, Syphilis
Drik og Masturbation	Drik, Masturbation
Drik og Misbrug af Morfin	Drik, Misbrug af Morfin
Drik og morfin	Drik, Misbrug af Morfin
Drik og Morfinmisbrug	Drik, Misbrug af Morfin
Drik og Næringssorg	Drik, Fattigdom
Drik og Nervøsitet	Drik, Angst
Drik og Øresygdom	Drik, Øresygdom
Drik og Overanstrængelse	Drik, Overanstrængelse
Drik og Rygmarvssygdom	Drik, Rygmarvssygdom
Drik og Selvbesinittelse	Drik, Masturbation
Drik og Selvbesmittelse	Drik, Masturbation
Drik og Selvbesrnittelse	Drik, Masturbation
Drik og Sindsindtryk	Drik, Nervechok
Drik og smertelige Sindsindtryk	Drik, Nervechok
Drik og Sorg	Drik, Sorg
Drik og Stød paa Hovedet	Drik, Hjernerystelse
Drik og Syphilis	Drik, Syphilis

Table 4: Terms harmonised *(continued)*

Before: 1455 unique categories	After: 705 unique categories
Drik og Udsvævelse	Drik, Seksualitet
Drik og ulykkeligt Ægteskab	Drik, Kærlighedssorg
Drik og uordentlig Levnet	Drik, Livsstil
Drik og uordentligt Levnet	Drik, Livsstil
Drik og uordentligt Liv	Drik, Livsstil
Drik og Usædelighed	Drik, Sexarbejde
Drik og Usedelighed	Drik, Sexarbejde
Drik, Arv og forudgaaende Sindssygdom	Drik, Arv, Tidligere Sindssygdom
Drik, Beruselse	Drik
Drik, Sindsindtryk og legemlig Sygdom3)	Drik, Nervechok, Fysisk Sygdom
Drik, Syphilis og Contusion ved Ulykkestilfælde	Drik, Syphilis, Ulykke
Drink og uordentligt Liv	Drik, Livsstil
Dyspepsi	Fordøjelsesproblemer
Dyspepsi og Apoplexi	Fordøjelsesproblemer, Slagtilfælde
Dyspepsi og Blodtab	Fordøjelsesproblemer, Anæmi
Eklampsi	Graviditet
Ektopia vericæ	Fysisk Handicap
Empyem	Lungebetændelse
Empyem og malaria	Lungebetændelse, Malaria
En eller flere af de ovennævnte	MULTIPLE
En eller flere af de ovennævnte Aarsager er opgivet for	MULTIPLE
En eller flere årsaker er opgitt for	MULTIPLE
En eller flere årsaker opgitt for	MULTIPLE
En eller flere årsaker or opgitt for	MULTIPLE
En eller flere av de oven-staaende aarsaker er opgit for	MULTIPLE
En eller flere av de oven stående årsaker er opgitt for	MULTIPLE
En eller flere av de ovenstaaende aarsaker er opgit for	MULTIPLE
En eller flere av de ovenstående årsaker er opgitt for	MULTIPLE
Ensomhed	Familiesorg
Enterit	Enterit
Enteritis chr	Fordøjelsesproblemer
Epilepsi	Epilepsi
Epilepsi og Barselseng	Epilepsi, Puerperium
Epilepsi og Diegivning	Epilepsi, Diegivning
Epilepsi og Drik	Epilepsi, Drik
Epilepsi og Fængselsstraf	Epilepsi, Fængselsophold
Epilepsi og Febersygdom	Epilepsi, Infektionssygdom
Epilepsi og Kjerlighedssorg	Epilepsi, Kærlighedssorg
Epilepsi og Sindsindtryk	Epilepsi, Nervechok

Table 4: Terms harmonised *(continued)*

Before: 1455 unique categories	After: 705 unique categories
Epilepsi og Svangerskab	Epilepsi, Graviditet
Epilepsi, Degeneration og Drik	Epilepsi, Mental Handicap, Drik
Erysipelas	Erysipelas
Erysipelas	Rosen
Erysipelas faciei	Inflammation
Exantematisk Tyfus	Typhoidfeber
Fængselsophold	Fængselsophold
Fængselsstraf	Fængselsophold
Fængselsstraf og Diegivning	Fængselsophold, Diegivning
Fængselsstraf og Sindsindtryk	Fængselsophold, Nervechok
Fængselsstraf og smertelige Sindsindtryk	Fængselsophold, Nervechok
Fængselsstraf og Udsvævelser	Fængselsophold, Seksualitet
Fængselsstraf og uordentligt Liv	Fængselsophold, Livsstil
Fængselsstraf, Arrest	Fængselsophold
Fængselsstraf; tvangsarbeide, forbryderliv	Fængselsophold
Fængselsstraff etc	Fængselsophold
Fængselsstraff; tvangsarbeide, forbryderliv	Fængselsophold
Fængselstraf	Fængselsophold
Fail, støt på hodet etc	Hjernerystelse
Fald under Besvimelse	Anæmi, Ulykke
Fald, støt etc paa hodet	Hjernerystelse
Fald, støt etc paa hodet; commotio cerebri	Hjernerystelse
Fald, støt, etc paa hodet	Hjernerystelse
Fall, støt etc på hodet; commotio cerebri	Hjernerystelse
Fall, støt på hodet etc	Hjernerystelse
Familiedisposition	Arv
Familiesorg	Familiesorg
Familiestrid	Familiesorg
Familiestridigheder	Familiesorg
Familietvist	Familiesorg
Fattigdom etc	Fattigdom
Fattigdom og Ondtliden	Fattigdom, Forkølelse
Fattigdom og Overanstrængelse	Fattigdom, Overanstrængelse
Fattigdom og slit	Fattigdom, Overanstrængelse
Fattigdom, ondtliden	Fattigdom, Forkølelse
Feber	Infektionssygdom
Febersygdom	Infektionssygdom
Febersygdom, Mæslinger etc	Infektionssygdom
Febersygdom, Mæslinger, Skarlagensfeber, Rosen etc	Infektionssygdom

Table 4: Terms harmonised *(continued)*

Before: 1455 unique categories	After: 705 unique categories
Febersygdomme	Infektionssygdom
Feilslagne Forhaabninger	Ærgelse
Flegmone	Inflammation
Forbrænding	Forbrænding
Forbrydelser	Fængselsophold
Forbryderisk Levnet, Fængselsstraf	Fængselsophold
Fordøielsessygdom	Fordøjelsesproblemer
Forførelse	Seksualitet
Forfrysning	Forfrysning
Forkjert Opdragelse og legemlig Svækkelse	Socialt Udsat, Svækkelse
Forkjølelse	Forkølelse
Forkjølelse efter Falden i Vandet	Forkølelse
Forkjølelse efter Falden i Våndet	Forkølelse
Forlis	Ulykke
Forskjellige svækkende indflydelser	Svækkelse
Fortidlig Senescens	Demens
Fosfornekrose	Forgiftning
Fractura cranii	Hjerneskallebrud
Fractura cruris	Fraktur
Fractura femoris	Fraktur
Fractura malleoli og Ondtliden	Fraktur, Forkølelse
Fraktur	Fraktur
Frihetsberøvelse, fængselsstraf	Fængselsophold
Frygt for Aflæggelse af falsk Ed	Angst
Frygt for Straf	Angst
Fysiologiske utviklingsprosesser	Udviklingshæmmet
Gastriske Sygdomme	Fordøjelsesproblemer
Gastrit	Gastrit
Gastrit (kronisk), gastroenterit	Gastrit, Gastroenterit
Gastroenterit	Gastroenterit
Genitalblødning (abort?)	Abort
Gigtfeber	Gigtfeber
Gigtfeber (forudgaaet)	Gigtfeber
Gigtfeber og Struma	Gigtfeber, Hyperthyreose
Giktfeber	Gigtfeber
Gjennemgaaet	Gjennemgaaet
Glaukom og døvhet	Synshæmmet, Hørehæmmet
Gonorré	Gonorrhoe
Gonorré, uretrit	Gonorrhoe, Urinorgansygdom

Table 4: Terms harmonised *(continued)*

Before: 1455 unique categories	After: 705 unique categories
Gonorrhoe	Gonorrhoe
Græmmelse	Græmmelse
Graviditet Besvangring	Graviditet
Graviditet og Besvangring	Graviditet
Graviditet, Barselseng og Dieeivning	Graviditet, Puerperium, Diegivning
Graviditet, barselseng og Diegivning	Graviditet, Puerperium, Diegivning
Graviditet, Barselseng og Diegivning	Graviditet, Puerperium, Diegivning
Graviditet, fødsel, barselseng og diegivning	Graviditet, Puerperium, Diegivning
Gul Feber	Gul Feber
Haardt arbeide i solstik	Solstik
Halssygdom	Halssygdom
Handelsspekulationer, uheldige	Pengespekulation
Herpes zoster	Herpes zoster
Hierneapoplexi	Slagtilfælde
Hjemve	Familiesorg
Hjerneapoplexi	Slagtilfælde
Hjerneatrofi	Demens
Hjernebetændelse	Hjernebetændelse
Hjernebetændelse i Barndommen	Hjernebetændelse i Barndommen
Hjernebetændelse i Barndomraen	Hjernebetændelse i Barndommen
Hjernebetændelse og Abort	Hjernebetændelse, Abort
Hjernebetændelse og Mæslinger	Hjernebetændelse, Mæslinger
Hjernebetændelse og Selvbesmittelse	Hjernebetændelse, Masturbation
Hjernefeil, -svulst og manglende Udvikling	Hjernefejl, Hjernesvulst, Udviklingshæmmet
Hjernefeil, kronisk, organisk	Hjernefejl
Hjernefeil, organisk	Hjernefejl
Hjernefeil, organiske	Hjernefejl
Hjerneforandringer, organiske	Hjernefejl
Hjerneincitation	Hjernesygdom
Hjernekongestioner	Slagtilfælde
Hjernelidelse	Hjernesygdom
Hjernelidelse i Forbindelse med medfødte Innervationsforstyrrelser	Diverse Handicap
Hjernerystelse	Hjernerystelse
Hjerneskallebrud (forudgaaet)	Hjerneskallebrud
Hjerneslag	Slagtilfælde
Hjernesvulst	Hjernesvulst
Hjernesygdom	Hjernesygdom
Hjernesygdom (organisk)	Hjernesygdom
Hjernesygdom i Barndommen	Hjernesygdom i Barndommen

Table 4: Terms harmonised *(continued)*

Before: 1455 unique categories	After: 705 unique categories
Hjernevatersot og Stød paa Hovedet	Hjernebetændelse, Hjernerystelse
Hjertefeil	Hjertefejl
Hjertefeil og Nyresygdom	Hjertefejl, Nyresygdom
Hjertefeil, organisk	Hjertefejl
Hjertesygdom	Hjertesygdom
Hjertesygdom og Nyresygdom	Hjertesygdom, Nyresygdom
Hjertesygdomme	Hjertesygdom
Hodepine	Hovedpine
Hovedpine	Hovedpine
Hovedpine, chronisk	Hovedpine
Hovedpine, kronisk	Hovedpine
Hugormbid	Ulykke
Huslige Bekymringer og Ondtliden	Fattigdom, Forkølelse
Hydrocephalus	Hjernebetændelse
Hyperæmia cerebri	Overanstrængelse
Hysteri	Hysteri
Hysteri og Degeneration	Hysteri, Mental Handicap
Hysteri og Drik	Hysteri, Drik
Hysteri og Kjærlighedssorg	Hysteri, Kærlighedssorg
Hysteri og Mavesaar	Hysteri, Mavesaar
Hysteri og Syphilis	Hysteri, Syphilis
Hysteri og Underlivssygdom og Sindsindtryk	Hysteri, Underlivssygdom, Sindsindtryk
Hysteri uden paavist Degeneration	Hysteri
Hysteri, neurasteni	Hysteri, Neurasteni
Hystero-Epilepsi	Hysteri, Epilepsi
I den fjernere Slægt forekom Sindssygdom	Arv
I den nærmeste Slægt forekom Sindssygdom	Arv
Idioti, medfødt	Mental Handicap
Idiotisme	Mental Handicap
Imbecilitit og Smaa evner	Mental Handicap
Imbecillitet	Mental Handicap
Imbecillitet	Udviklingshæmmet
Imbecillitet og Degeneration	Mental Handicap
Imbecillitet og Smaa evner	Mental Handicap
Imbecillitet Psykisk svakhet	Mental Handicap
Imbecillitet Smaa evner	Mental Handicap
Imbecillitet, Psykisk svakhet	Mental Handicap
indlagt til Observation	Observation
indlagt til Observationer	Observation

Table 4: Terms harmonised *(continued)*

Before: 1455 unique categories	After: 705 unique categories
indlagte til Observation	Observation
Indlagte til Observation	Observation
Infantilisme	Udviklingshæmmet
Infektionssygdom	Infektionssygdom
Influensa	Influenza
Influenza	Influenza
Influenza og Asthma	Influenza, Asthma
Influenza og Chlorose	Influenza, Anæmi
Influenza og Sindsindtryk	Influenza, Sindsindtryk
Innlagt til observasjon	Observation
Insolation	Solstik
Insufficientia renalis	Nyresygdom
Intoxikationer	Intoxikationer
Invaliditet	Udviklingshæmmet
Ischias	Rygsmerte
Jalousi	Kærlighedssorg
Kaffedrikning, overdreven	Misbrug af Kaffe
Kikhoste	Kikhoste
Kirurgisk Operation	Operation
Kjærestesorg	Kærlighedssorg
Kjærestesorg og Klorose	Kærlighedssorg, Anæmi
Kjærlighedssorg	Kærlighedssorg
Kjærlighedssorg og legemlig Sygdom	Kærlighedssorg, Fysisk Sygdom
Kjærlighedssorg og religiøse Indflydelser	Kærlighedssorg, Religiøse Indflydelser
Kjærlighedssorg og Skræk	Kærlighedssorg, Angst
Kjærlighedssorger	Kærlighedssorg
Kjønsudsvævelse	Seksualitet
Klimakteriet	Overgangsalder
Klimakterium	Overgangsalder
Klimatfeber, organisk Hjertesygdom og Tuberkulose	Infektionssygdom, Hjertesygdom, Tuberkulose
Klorose	Anæmi
Klorose og Anæmi	Anæmi
Knæledsbetændelse	Rheumatisme
Konflikter og bekymringer	Konflikter, Bekymringer
konsanguint Ægteskab	Kosanguinitet
Kontusion	Ulykke
Koprostase	Fordøjelsesproblemer
Kræft	Kræft
Kramper	Epilepsi

Table 4: Terms harmonised *(continued)*

Before: 1455 unique categories	After: 705 unique categories
Kramper i barndommen	Epilepsi
Kramper i Barnealderen	Epilepsi
Kreft	Kræft
Krigsfrygt	Angst
Krigsfrygt, militærtjeneste, neutralitetsvagt	Angst, Militærtjeneste
Krigsfrygt, militærtjeneste, nøitralitetsvakt	Angst, Militærtjeneste
Krigsfrykt	Angst
kronisk Bronkit	Bronkit
Kronisk Bronkit	Bronkit
kronisk Diarré	Diarré
Kronisk Enterit	Enterit
kronisk legemlig Sygdom	Fysisk Sygdom
Kronisk mavekatarr og dyspepsi	Gastrit, Fordøjelsesproblemer
Kronisk mavekatarr; dyspepsi	Gastrit, Fordøjelsesproblemer
Kronisk nefrit	Nyrebetændelse
Kronisk Otit	Ørebetændelse
Kronisk Rheumatisme	Rheumatisme
Kusma	Kusma
Kyfose	Kyfose
Læggesaar	Overanstrængelse
Læseri	Læseri
Læsion av hodet	Hjernerystelse
Læsioner af Hovedet	Hjernerystelse
Læsioner av hodet	Hjernerystelse
Læsioner og mangelfuld Pleie	Vold, Mishandling under Opvækst
Lammelse	Lammelse
Langvarig Diegivning	Diegivning
Langvarig Diegivning og tidligere Sindssygdom	Diegivning, Tidligere Sindssygdom
Langvarig nervøsitet	Angst
Langvarig sykeleie	Svækkelse
Lateris	SUBTOTAL
Legemlig Afkræftelse	Afkræftelse
Legemlig Afkræftelse og smertelige Sindsindtryk	Afkræftelse, Nervechok
Legemlig Beskadigelse	Ulykke
Legemlig Overanstrengelse og Sindsindtryk	Overanstrængelse, Nervechok
Legemlig Svækkelse	Svækkelse
Legemlig Svaghed	Svækkelse
Legemlig Svaghed og Anæmi	Svækkelse, Anæmi
Legemlig Svaghed og andre Svækkelsestilstande	Svækkelse

Table 4: Terms harmonised *(continued)*

Before: 1455 unique categories	After: 705 unique categories
Legemlig Svaghed og Apoplexi	Svækkelse, Slagtilfælde
Legemlig Svaghed og Difteri	Svækkelse, Difteri
Legemlig Svaghed og eurastheni	Svækkelse, Neurasteni
Legemlig Svaghed og Gigtfeber	Svækkelse, Gigtfeber
Legemlig Svaghed og Hjernebetændelse	Svækkelse, Hjernebetændelse
Legemlig Svaghed og Hjertesygdom	Svækkelse, Hjertesygdom
Legemlig Svaghed og Hysteri	Svækkelse, Hysteri
Legemlig Svaghed og Influenza	Svækkelse, Influenza
Legemlig Svaghed og Mavekatarrh	Svækkelse, Mavesaar
Legemlig Svaghed og Menstruationsuordener	Svækkelse, Menstruationsuordener
Legemlig Svaghed og Nyresygdom	Svækkelse, Nyresygdom
Legemlig Svaghed og økonomiske Bekymringer	Svækkelse, Fattigdom
Legemlig Svaghed og Otit	Svækkelse, Ørebetændelse
Legemlig Svaghed og Stød paa Hovedet	Svækkelse, Hjernerystelse
Legemlig Svaghed og Sygdom	Svækkelse, Fysisk Sygdom
Legemlig Svaghed og Tæring	Svækkelse, Tuberkulose
Legemlig Svaghed og Underlivssygdom	Svækkelse, Underlivssygdom
Legemlig Svaghed og Uterinsygdom	Svækkelse, Uterinsygdom
Legemlig svakhet og andre svækkelsestilstande	Svækkelse
Legemlig svakhet, avkræftelse	Svækkelse, Afkræftelse
Legemlig svekkelse	Svækkelse
Legemlig sygdom	Fysisk Sygdom
Legemlig Sygdom	Fysisk Sygdom
Legemlig Sygdom og Barselseng	Fysisk Sygdom, Puerperium
Legemlig Sygdom og deprimerende Sindsindtryk	Fysisk Sygdom, Nervechok
Legemlig Sygdom og forudgaaende Sindssygdom	Fysisk Sygdom, Tidligere Sindssygdom
Legemlig Sygdom og Græmmelse derover	Fysisk Sygdom
Legemlig Sygdom og Nervøsitet	Fysisk Sygdom, Angst
Legemlig Sygdom og Overanstrængelse	Fysisk Sygdom, Overanstrængelse
Legemlig Sygdom og Selvbesrnittelse	Fysisk Sygdom, Masturbation
Legemlig Sygdom og Sindsindtryk	Fysisk Sygdom, Nervechok
Legemlig Sygdom og Skræk	Fysisk Sygdom, Angst
Legemlig Sygdom og Skuffelse	Fysisk Sygdom, Ærgelse
Legemlig Sygdom og smertelige Sindsindtryk	Fysisk Sygdom, Nervechok
Legemlig Sygdom, Nervøsitet og Sindsindtryk	Fysisk Sygdom, Angst, Nervechok
Legemlig Sygdom, Overanstrængelse og Sindsindtryk	Fysisk Sygdom, Overanstrængelse, Nervechok
Legemlig Sygdom, Overanstrengelse og Sindsindtryk	Fysisk Sygdom, Overanstrængelse, Nervechok
Legemlig Sygdom, Sindsindtryk og langvarig Diegivning	Fysisk Sygdom, Nervechok, Diegivning
Legemlig Sygdom, Svækkelse, Masturbation og Sindsindtryk	Fysisk Sygdom, Svækkelse, Masturbation, Nervechok

Table 4: Terms harmonised *(continued)*

Before: 1455 unique categories	After: 705 unique categories
Legemlig Sygdom2)	Fysisk Sygdom
Legemlig sykdom	Fysisk Sygdom
Legemlige Smerter	Fysiske Smerter
Legemlige sygdomme	Fysisk Sygdom
Legemlige Sygdomme	Fysisk Sygdom
Liens ventriculi	Mavesaar
Livmoderblødning	Uterinsygdom
Livmodersygdom	Uterinsygdom
Lues	Syphilis
Lues og Alder	Syphilis, Alderdom
Lungebetændelse	Lungebetændelse
Lungebetendelse	Lungebetændelse
Lungesvindsot	Tuberkulose
Lungetuberkulose	Tuberkulose
Lupus	Lupus
Lymfangit	Lymphangitis
Lymphangit	Lymphangitis
Lynnedslag	Ulykke
Mæslinger	Mæslinger
Malaria	Malaria
Malaria og Ondtliden	Malaria, Forkølelse
Mangelfuld Hjerneudvikling	Udviklingshæmmet
Mangelfuld Udvikling	Udviklingshæmmet
Mangelfuld Udvikling af Hjernen	Udviklingshæmmet
Mangelfuld Udvikling af Hjernen (Microcephali)	Microcephali
Marasme	Marasmus
Marasmus	Marasmus
Marasmus sen	Marasmus
Mastit	Brystbetændelse
Masturbation	Masturbation
Masturbation og Drik	Masturbation, Drik
Masturbation og Neurasteni	Masturbation, Neurasteni
Masturbation og Sindsindtryk	Masturbation, Nervechok
Masturbation og Stød paa Hovedet	Masturbation, Hjernerystelse
Masturbation, utsvævelser	Masturbation, Seksualitet
Mavekatarr	Gastrit
Mavekatarr og Sindsindtryk	Mavesaar, Sindsindtryk
Mavekræft	Kræft
Mavesaar	Mavesaar

Table 4: Terms harmonised *(continued)*

Before: 1455 unique categories	After: 705 unique categories
Mavesaar og endometrit	Mavesaar, Endometriose
Mavesygdom, chronisk	Mavesygdom
Medfødt blindhet	Synshæmmet
Medfødt Degeneration	Mental Handicap
Medfødt Svaghed	Udviklingshæmmet
Medfødt Svaghed og Trauma capitis	Udviklingshæmmet, Hjernerystelse
Medfødt Svaghed, Drik og religiøse Skrupler	Udviklingshæmmet, Drik, Religiøse Grublerier
Medfødt svakhet	Udviklingshæmmet
Medfødt Sygdom	Diverse Handicap
Medføtt svakhet	Mental Handicap
Menigit	Hjernebetændelse
Meningit	Hjernebetændelse
Meningitis cerebrospinalis	Hjernebetændelse
Menorrhagi	Menorrhagi
Menostasi	Menstruationsuordener
Menstruationsuordener	Menstruationsuordener
Menstruationsuordener og Livmodersygdom	Menstruationsuordener, Uterinsygdom
Menstruationsuordener og religiøse Indflydelser	Menstruationsuordener, Religiøse Indflydelser
Menstruationsuordener Uterinlidelse	Menstruationsuordener, Uterinsygdom
Menstruationsuordener, Livmodersygdom	Menstruationsuordener, Uterinsygdom
Menstruationsuordner	Menstruationsuordener
Metastase ved Kusma	Kusma
Metorrhagi	Menorrhagi
Metrit	Uterinsygdom
Metrorrhagi	Menorrhagi
Migræne	Hovedpine
Mikrokefali	Microcephali
Militærtjeneste	Militærtjeneste
Militærtjeneste (neutralitetsvagt)	Militærtjeneste
Mindreverdige anlegg	Udviklingshæmmet
Misbrug af Chloral	Misbrug af Chloral
Misbrug af Narcotica	Misbrug af Morfin
Misbruk av tobak	Misbrug af Tobak
Mishandling	Mishandling
Mishandling i Barneaarene	Mishandling under Opvækst
Mishandling og Ondtlidende	Mishandling, Forkølelse
Mishandling, Ondtliden, legemlig Overanstrængelse	Mishandling, Forkølelse, Overanstrængelse
Modgang	Fattigdom
Morbilli	Mæslinger

Table 4: Terms harmonised *(continued)*

Before: 1455 unique categories	After: 705 unique categories
Morbus Basedowi	Hyperthyreose
Morbus Brighti	Nefrit
Morphinisme	Misbrug af Morfin
Motgang	Fattigdom
Næringsorg, økonomisk Bekymring	Fattigdom
Næringssorg	Fattigdom
Næringssorg og Bronchit	Fattigdom, Bronkit
Næringssorg og huslige Bekymringer	Fattigdom
Næringssorg og økonomisk Bekymring	Fattigdom
Næringssorg og ulykkeligt Ægteskab	Fattigdom, Kærlighedssorg
Næringssorg, økonomisk Bekymring	Fattigdom
Næringssorg, økonomiske Bekymringer	Fattigdom
Næringssorg, Sorg, Ærgrelser	Fattigdom, Sorg, Ærgelse
Næringssorger	Fattigdom
Nattevaag og Diegivning	Søvnløshed, Diegivning
Nattevaag og Indvirkning af stærk Hede paa Hovedet	Søvnløshed, Solstik
Nattevaag og Sindsindtryk	Søvnløshed, Nervechok
Nattevaagen	Søvnløshed
Nattevaak	Søvnløshed
Nattevaak og natarbeide	Søvnløshed, Overanstrængelse
Nattevaak og Natarbeide	Søvnløshed, Overanstrængelse
Nattevåken	Søvnløshed
Nefrit	Nefrit
Nervefeber	Nervechok
Nervesvækkelse	Svækkelse
Nervesvækkelse og kronisk Artrit	Svækkelse, Artrit
Nervesvækkelse og legemlig Svaghed	Svækkelse
Nervesvaghed	Svækkelse
Nervøs Disposition	Angst
Nervøs Disposition og Fængselsstraf	Angst, Fængselsophold
Nervøs Konstitution	Angst
Nervøs konstitution Degeneration	Angst, Mental Handicap
Nervøs Svækkelse	Svækkelse
Nervøs Svækkelse og aandelig Overanstrængelse	Svækkelse, Overanstrængelse
Nervøsitet	Angst
Nervøsitet + Blindtarmbetændelse	Angst, Appendicit
Nervøsitet + Tarmkatarr	Angst, Mavesaar
Nervøsitet og Kjærlighedssorg	Angst, Kærlighedssorg
Nervøsitet, Disposition og Nervøs Svækkelse	Angst, Svækkelse

Table 4: Terms harmonised *(continued)*

Before: 1455 unique categories	After: 705 unique categories
Neuralgier i Hovedet	Neuralgi
Neuralgiske Hovedsmerter	Neuralgi
Neurasteni	Neurasteni
Neurasteni og Hysteri	Neurasteni, Hysteri
Neurasteni og Sindsindtryk	Neurasteni, Nervechok
Neurastheni	Neurasteni
Neurastlieni	Neurasteni
Nevrastemi	Neurasteni
Nevrastheni	Neurasteni
Nød og Modgang	Fattigdom
Nyresygdom	Nyresygdom
Nyresygdom og Hjertefeil og Sindsindtryk	Nyresygdom, Hjertefejl, Sindsindtryk
Nyresykdom	Nyresygdom
Observander	Observation
Øien- og øresygdom, døvhet etc	Øiensygdom, Øresygdom, Hørehæmmet
Øienbetændelse	Øienbetændelse
Øienoperation	Operation
Øienoperation (forudgaaet)	Operation
Øiensygdom	Øiensygdom
Øiensygdom og Sindsindtryk	Øiensygdom, Sindsindtryk
Økonomiske bekymringer	Fattigdom
Økonomiske Bekymringer	Fattigdom
Omflakkende liv	Livsstil
Onani	Masturbation
Onani og masturbation	Masturbation
Onani og Masturbation	Masturbation
Ondtliden	Forkølelse
Ondtliden og legemlig Sygdom	Forkølelse, Fysisk Sygdom
Ondtliden og Nød	Forkølelse, Fattigdom
Ondtliden, afkræftende Sygdomme	Forkølelse, Afkræftelse
Operasjon	Operation
Operation	Operation
Operation og Kastration	Operation
Operativt Indgreb	Operation
Operativt Indgreb i Forbindelse med Alkoholisme	Operation, Drik
Ophold i utlandet	Ophold i utlandet
Ørebetendelse	Ørebetændelse
Ørelidelse	Øresygdom
Øresygdom	Øresygdom

Table 4: Terms harmonised *(continued)*

Before: 1455 unique categories	After: 705 unique categories
Organisk Hjerneaffektion	Hjernesygdom
Organisk Hjerneaffektion (Meningit)	Hjernebetændelse
Organisk Hjertesygdom	Hjertesygdom
Organisk Hjertesygdom og Sindsindtryk	Hjertesygdom, Sindsindtryk
Osteomyelit	Osteomyelit
Otit	Ørebetændelse
Overanstrængelse	Overanstrængelse
Overanstrængelse (fysisk eller psykisk)	Overanstrængelse
Overanstrængelse (legemlig eller aandelig)	Overanstrængelse
Overanstrængelse (legemlig eller åndelig)	Overanstrængelse
Overanstrængelse (legemlig og aandelig)	Overanstrængelse
Overanstrængelse (legemlig og aandelig):	Overanstrængelse
Overanstrængelse og (smertelige) Sindsindtryk	Overanstrængelse, Nervechok
Overanstrængelse og Afkræftelse	Overanstrængelse, Afkræftelse
Overanstrængelse og Armod	Overanstrængelse, Fattigdom
Overanstrængelse og Barselseng	Overanstrængelse, Puerperium
Overanstrængelse og Fattigdom	Overanstrængelse, Fattigdom
Overanstrængelse og Kjærestesorg	Overanstrængelse, Kærlighedssorg
Overanstrængelse og Kjærlighedssorg	Overanstrængelse, Kærlighedssorg
Overanstrængelse og legcmlig Sygdom	Overanstrængelse, Fysisk Sygdom
Overanstrængelse og Nattevaag	Overanstrængelse, Søvnløshed
Overanstrængelse og Nattevaag, Søvnløshed	Overanstrængelse, Søvnløshed
Overanstrængelse og Nattevaagen	Overanstrængelse, Søvnløshed
Overanstrængelse og Nød	Overanstrængelse, Fattigdom
Overanstrængelse og Ondtliden	Overanstrængelse, Forkølelse
Overanstrængelse og Operation	Overanstrængelse, Operation
Overanstrængelse og religiøs Paavirkning	Overanstrængelse, Religiøse Indflydelser
Overanstrængelse og religiøse Indflydelser	Overanstrængelse, Religiøse Indflydelser
Overanstrængelse og Selvbesmittelse	Overanstrængelse, Masturbation
Overanstrængelse og Sindsindtryk	Overanstrængelse, Nervechok
Overanstrængelse og Sindslidelser	Overanstrængelse, Sindslidelser
Overanstrængelse og Sindssindtryk	Overanstrængelse, Nervechok
Overanstrængelse og Sorg	Overanstrængelse, Sorg
Overanstrængelse og tidligere Sindssygdom	Overanstrængelse, Tidligere Sindssygdom
Overanstrængelse Ondtliden	Overanstrængelse, Forkølelse
Overanstrængelse Ondtliden med stillesidende Arbeide	Overanstrængelse, Forkølelse
Overanstrængelse, aandelig	Overanstrængelse
Overanstrængelse, legemlig	Overanstrængelse
Overanstrængelse, Nattevaag og Hjemve	Overanstrængelse, Søvnløshed, Familiesorg

Table 4: Terms harmonised *(continued)*

Before: 1455 unique categories	After: 705 unique categories
Overanstrængelse, Nattevaagen og Sindsindtryk	Overanstrængelse, Søvnløshed, Nervechok
Overanstrængelse, Nattevaagen og Sorg	Overanstrængelse, Søvnløshed, Sorg
Overanstrængelse, Nattevaagen, Sindsindtryk	Overanstrængelse, Søvnløshed, Nervechok
Overanstrængelse, Nattevaagen, Sindsindtryk ,	Overanstrængelse, Søvnløshed, Nervechok
Overanstrængelse, Ondtliden, afkræftende Sygdom	Overanstrængelse, Forkølelse, Afkræftelse
Overanstrængelse, Ondtliden, afkræftende Sygdomme	Overanstrængelse, Forkølelse, Afkræftelse
Overanstrængelse, Sindsindtryk og Nattevaag	Overanstrængelse, Nervechok, Søvnløshed
Overanstrengdlse (legemlig eller aandelig)	Overanstrængelse
Overanstrengelse	Overanstrængelse
Overanstrengelse (fysisk eller psykisk)	Overanstrængelse
Overanstrengelse (legemlig eller aandelig)	Overanstrængelse
Overanstrengelse (legemlig eller åndelig)	Overanstrængelse
Overanstrengelse (legemlig og aandelig)	Overanstrængelse
Overanstrengelse og Kjærlighedssorg	Overanstrængelse, Kærlighedssorg
Overanstrængelse	Overanstrængelse
Overanstrængelse og legemlig Sygdom	Overanstrængelse, Fysisk Sygdom
Overanstrængelse og Sindsindtryk	Overanstrængelse, Nervechok
Overanstrængelse og Trauma capitis	Overanstrængelse, Hjernerystelse
Overarbeide	Overanstrængelse
Overført	SUBTOTAL
Pæderasti	Seksualitet
Panaritium	Betændelse i Haanden
Panaritium og Sindsindtryk	Betændelse i Haanden, Sindsindtryk
Paralysis agitans	Parkinsons
Parametrit	Underlivssygdom
Parese	Lammelse
Parotit	Infektionssygdom
Parotit	Kusma
Periodisk Hovedpine	Hovedpine
Periostit	Skinnebenssår
Phlegmone	Phlegmone
Phrenoepilepsia	Epilepsi
Pleurit	Lungebetændelse
Pludselig sindsbevægelse (skræk etc)	Angst
Pludselig Sindsbevægelse (Skræk etc)	Angst
Pludselig sindsbevægelse (skræk, vrede etc)	Angst
Pludselig sindsbevægelse, Skræk, vrede etc	Angst
Pneumoni	Lungebetændelse
Pneumoni og Sindsindtryk	Lungebetændelse, Sindsindtryk

Table 4: Terms harmonised *(continued)*

Before: 1455 unique categories	After: 705 unique categories
Poliomyelit	Poliomyelit
Politik	Politisk Ekstremisme
Politisk Exaltation	Politisk Ekstremisme
Prolapsus recti	Prolaps
Prolapsus uteri	Prolaps
Psychopathisk konstitusjon	Psykopati
Psykisk indvirkning	Psykisk Sygdom
Psykiske årsaker	Psykisk Sygdom
Psykopatisk konstitution	Psykopati
Pubertetsutvikling	Pubertetsutvikling
Pyæmi	Pyæmi
Pyæmi og Sindsindtryk	Pyæmi, Sindsindtryk
Pyo-pneumothorax	Tuberkulose
Pyogen infektion	Inflammation
Rakit	Rakit
Refrigerium	Forkølelse
Religiøs Paavirkning	Religiøse Indflydelser
Religiøs Paavirkning og Overanstrængelse	Religiøse Indflydelser, Overanstrængelse
Religiøse grublerier	Religiøse Grublerier
Religiøse Grublerier	Religiøse Grublerier
Religiøse Grublerier og Bekymringer	Religiøse Grublerier, Bekymringer
Religiøse Grublerier og Næringssorg	Religiøse Grublerier, Fattigdom
Religiøse Grublerier og Selvbesmittelse	Religiøse Grublerier, Masturbation
Religiøse Grublerier og Skrupler	Religiøse Grublerier
Religiøse grublerier, usund religiøs paavirkning	Religiøse Grublerier
Religiøse grublerier, usund religiøs påvirkning	Religiøse Grublerier
Religiøse grublerier; usund, religiøs paavirkning	Religiøse Grublerier
Religiøse Indflydelser	Religiøse Indflydelser
Religiøse Indflydelser og Diegivning	Religiøse Indflydelser, Diegivning
Religiøse Indflydelser og indbildt Næringssorg	Religiøse Indflydelser, Fattigdom
Religiøse Indflydelser og legemlig Sygdom	Religiøse Indflydelser, Fysisk Sygdom
Religiøse Indflydelser og Næringssorg	Religiøse Indflydelser, Fattigdom
Religiøse Indflydelser og Selvbesmittelse	Religiøse Indflydelser, Masturbation
Religiøse Indflydelser og Sorg	Religiøse Indflydelser, Sorg
Religiøse Indflydelser, Grublerier	Religiøse Indflydelser, Religiøse Grublerier
Religiøse Indflydelser, Grublerier, Skrupler	Religiøse Indflydelser, Religiøse Grublerier
Religiøse Indflydelser, Hovedpine, Overanstrængelse	Religiøse Indflydelser, Hovedpine, Overanstrængelse
Religiøse Indflydelser, Skrupler, Grublerier	Religiøse Indflydelser, Religiøse Grublerier
Religiøse Sindsindtryk	Religiøse Indflydelser

Table 4: Terms harmonised *(continued)*

Before: 1455 unique categories	After: 705 unique categories
Religiøse Skrupler	Religiøse Grublerier
Retroflexio nteri	Uterinsygdom
Retsforfølgning og Straf	Fængselsophold
Rhakit	Rakit
Rheum acut	Rheumatisme
Rheum acut, Sindsindtryk og langvarig Diegivning	Rheumatisme, Sindsindtryk, Diegivning
Rheum chr	Rheumatisme
Rheumat acut	Rheumatisme
Rheumatisme	Rheumatisme
Rheumatismus	Rheumatisme
Rheumatismus acutus og Lithiasis og Diabetes mellitus	Rheumatisme, Nyresygdom, Diabetes
Rosen	Rosen
Ruptura urethræ	Urinorgansygdom
Rygmarvssygdom	Rygmarvssygdom
Rygmarvssygdom og Tabes dorsalis	Rygmarvssygdom, Neurosyphilis
Rygmarvsygdom	Rygmarvssygdom
Sammenlagt	SUBTOTAL
Samvittighetsnag	Ærgelse
Scarlatina	Skarlagensfeber
Scarlatina og Sindsindtryk	Skarlagensfeber, Sindsindtryk
Scoliose og Svaghed	Scoliose, Afkræftelse
Seabies	Scabies
Selvbebreidelse	Ærgelse
Selvbesmittelse	Masturbation
Selvbesmittelse i Forbindelse med andre Aarsager, saasom Drik, smertelige Sindsindtryk, Syphilis	Masturbation, Drik, Nervechok, Syphilis
Selvbesmittelse og Drik	Masturbation, Drik
Selvbesmittelse og Drik, smertelige Sindsindtryk	Masturbation, Drik, Nervechok
Selvbesmittelse og Hysteri	Masturbation, Hysteri
Selvbesmittelse og Kjærestesorg	Masturbation, Kærlighedssorg
Selvbesmittelse og Kjærlighedssorg	Masturbation, Kærlighedssorg
Selvbesmittelse og kjønslige Udskeielser	Masturbation, Livsstil
Selvbesmittelse og Misbrug af Merkur	Masturbation, Misbrug af Merkur
Selvbesmittelse og religiøse Indflydelser, smertelige Sindsindtryk	Masturbation, Religiøse Indflydelser, Nervechok
Selvbesmittelse og Samvittighedsskrupler	Masturbation, Ærgelse
Selvbesmittelse og Sindsindtryk	Masturbation, Nervechok
Selvbesmittelse og Skuffelse	Masturbation, Ærgelse
Selvbesmittelse og smertelige Indtryk	Masturbation, Nervechok
Selvbesmittelse og smertelige Sindsindtryk	Masturbation, Nervechok
Selvbesmittelse og Stød paa Hovedet	Masturbation, Hjernerystelse

Table 4: Terms harmonised *(continued)*

Before: 1455 unique categories	After: 705 unique categories
Selvbesmittelse og Sygdom	Masturbation, Fysisk Sygdom
Selvbesmittelse og Udsvævelse	Masturbation, Seksualitet
Selvbesmittelse og Udsvævelser	Masturbation, Seksualitet
Selvbesmittelse Sterilitet og Digestionsuordener	Masturbation, Sterilitet, Fordøjelsesproblemer
Selvbesmittelse, Sindsindtryk og Graviditet	Masturbation, Nervechok, Graviditet
Selvbesrnittelse	Masturbation
Senescens	Demens
Sexuelle Forstyrrelser	Seksualitet
Sexuelle Indflydelser (Selvbesmittelse, Impotents etc)	Seksualitet
Shok	Nervechok
Sinds indtryk	Nervechok
Sindsindtryk	Nervechok
Sindsindtryk (sorg, kjærlighetssorg, ærgrelse, motgang, skrupler)	Nervechok
Sindsindtryk og -lidelser	Nervechok, Sindslidelser
Sindsindtryk og Influenza	Sindsindtryk, Influenza
Sindsindtryk og legemlig Sygdom	Nervechok, Fysisk Sygdom
Sindsindtryk og Nattevaagen	Nervechok, Søvnløshed
Sindsindtryk og nervesjok	Nervechok
Sindsindtryk og Overanstrængelse	Nervechok, Overanstrængelse
Sindsindtryk og religiøse Grublerier	Nervechok, Religiøse Grublerier
Sindsindtryk og religiøse Indflydelser	Nervechok, Religiøse Indflydelser
Sindsindtryk og Sorger	Nervechok, Sorg
Sindsindtryk og Stød paa Hovedet	Nervechok, Hjernerystelse
Sindsindtryk og tidligere Sindssygdom	Nervechok, Tidligere Sindssygdom
Sindsindtryk, legemlig Sygdom og Overanstrengelse	Nervechok, Fysisk Sygdom, Overanstrængelse
Sindsindtryk, nervesjok	Nervechok
Sindsindtryk, Sorger og Bekymringer	Nervechok, Sorg, Bekymringer
Sindsindtryk; nervesjok	Nervechok
Sindslidelse	Sindslidelser
Sindslidelse og religiøse Skrupler	Sindslidelser, Religiøse Grublerier
Sindslidelser	Sindslidelser
Sindslidelser (økonomiske bekymringer, ulykkelige familieforhold, ulykkelig kjærlighet etc)	Sindslidelser
Sindslidelser (økonomiske bekymringer, ulykkelige familieforhold, ulykkelig kjærlighet etc) og sorg	Sindslidelser, Sorg
Sindslidelser (økonomiske bekymringer, ulykkelige familieforhold, ulykkelig kjærlighet etc), sorg	Sindslidelser, Sorg
Sindslidelser (økonomiske bekymringer, ulykkelige familieforhold, ulykkelig kjærligliet etc) og sorg	Sindslidelser, Sorg
Sindslidelser (økonomiske bekymringer,ulykkelige familieforhold, ulykkelig kjærlighet etc)	Sindslidelser
Sindslidelser og legemlig Sygdom	Sindslidelser, Fysisk Sygdom
Sindslidelser og smertelige Sindsindtryk	Sindslidelser, Nervechok
Sindslidelser og tidligere Sindssygdom	Sindslidelser, Tidligere Sindssygdom

Table 4: Terms harmonised *(continued)*

Before: 1455 unique categories	After: 705 unique categories
Sindslidelser, økonomiske bekymringer, ulykkelige familieforhold	Sindslidelser, Fattigdom, Familiesorg
Sindslidelser, smertelige Sindsindtryk	Sindslidelser, Nervechok
Sindssygdom forekom i nærpaararende Familie	Pårørende
Sindssygdom forekom i nærpaarørende Familie	Pårørende
Sindssygdom hos Nærpaarørende	Pårørende
Sindssygdom i nærpaarørende Familie	Pårørende
Sindssygdom, tidligere	Tidligere Sindssygdom
Sindssygdomme eller Nervesygdomme i fjernere Slegt	Pårørende
Sindssygdomme eller Nervesygdomme i nærmeste Slegt	Pårørende
sindssyge Mødre, Fædre eller Bedsteforældre	Pårørende
Sinnsinntryk; nervesjokk	Nervechok
Sinnslidelser (økonomiske bekymringer, ulykkelige familieforhold, ulykkelig kjærlighet etc)	Sindslidelser
Sinnslidelser (økonomiske bekymringer, ulykkelige familieforhold, ulykkelig kjærlighet etc) sorg	Sindslidelser, Sorg
Sinnslidelser (økonomiske bekymringer, ulykkelige familieforhold, ulykkelig kjærlighet etc), sorg	Sindslidelser, Sorg
Skadelig Behandling i Barndom	Mishandling under Opvækst
Skadelig Behandling i Barndom og Opvæxt	Mishandling under Opvækst
Skadelig miljø	Socialt Udsat
Skadet Behandling Barndommen	Mishandling under Opvækst
Skarlagensfeber	Skarlagensfeber
Skarlagensfeber (forudgaaet)	Skarlagensfeber
Skarlagensfeber og Øresygdom	Skarlagensfeber, Øresygdom
Skjægsop	Kusma
Skræk	Angst
Skræk hos Moderen under Svangerskabet	Pårørende
Skræk og anstrængelse under Forlis paa Søen	Angst, Ulykke
Skræk og Overarbeide	Angst, Overanstrængelse
Skræk, Næringssorg og anstrængende Arbeide	Angst, Fattigdom, Overanstrængelse
Skuffelse	Ærgelse
Skuffelse og Ærgrelse	Ærgelse
Skuffelser	Ærgelse
Slag	Slagtilfælde
Slet Ondragelse	Socialt Udsat
Slet opdragelse	Socialt Udsat
Slet Opdragelse	Socialt Udsat
Slet Opdragelse og Sindslidelse	Socialt Udsat, Sindslidelser
Sløvhed (medfødt)	Udviklingshæmmet
Smaa evner	Diverse Handicap
Smaa Evner	Diverse Handicap
Smertelige Sindsindtryk	Nervechok

Table 4: Terms harmonised *(continued)*

Before: 1455 unique categories	After: 705 unique categories
Smertelige sindsindtryk (kjærlighetssorg, ærgrelse etc)	Nervechok
Smertelige Sindsindtryk i Forbindelse med Anæmi, Menstruationsuordener, Selvbesmittelse	Nervechok, Anæmi, Menstruationsuordener, Masturbation
Smertelige Sindsindtryk i Forbindelse med andre Aarsager saasom Anæmi, Barselseng, Menstruation-suordener	Nervechok, Anæmi, Puerperium, Menstruationsuordener
Smertelige Sindsindtryk i Forbindelse med andre Aarsager, saasom Anæmi, Læseri og Selvbesmittelse	Nervechok, Anæmi, Læseri, Masturbation
Smertelige Sindsindtryk og Beruselse	Nervechok, Intoxikationer
Smertelige Sindsindtryk og Gigtfeber	Nervechok, Gigtfeber
Smertelige Sindsindtryk og legemlig Sygdom	Nervechok, Fysisk Sygdom
Smertelige Sindsindtryk og økonomisk Bekymring etc	Nervechok, Fattigdom
Smertelige Sindsindtryk og Ondtliden, Nattevaagen	Nervechok, Forkølelse, Søvnløshed
Smertelige Sindsindtryk og Overanstrængelse	Nervechok, Overanstrængelse
Smertelige Sindsindtryk og Stød paa Hovedet	Nervechok, Hjernerystelse
Smertelige Sindsindtryk, indbildt Næringssorg	Nervechok, Fattigdom
Smertelige Sindsindtryk, Sindslidelser	Nervechok, Sindslidelser
Smertelige Sindsindtryk, ulykkeligt Ægteskab, Næringssorg	Nervechok, Kærlighedssorg, Fattigdom
Smerter i Ryggen, langvarige	Rygsmerte
Smittefrygt	Smittefrygt
Sodomiteri	Seksualitet
Solstik	Solstik
Solstik, Indvirkning af Hede	Solstik
Solstikk	Solstik
Sorg	Sorg
Sorg og Ærgrelse	Sorg, Ærgelse
Sorg og Afkræftelse	Sorg, Afkræftelse
Sorg og Anger	Sorg, Ærgelse
Sorg og Arv	Sorg, Arv
Sorg og Bekymring	Sorg, Bekymringer
Sorg og Blegsot	Sorg, Anæmi
Sorg og Nattevaagen	Sorg, Søvnløshed
Sorg og Overanstrængelse	Sorg, Overanstrængelse
Sorg og religiøse Grublerier	Sorg, Religiøse Grublerier
Sorg og religiøse Skrupler	Sorg, Religiøse Grublerier
Sorg og Skræk	Sorg, Angst
Sorg og Syphilis	Sorg, Syphilis
Sorg, Kummer	Sorg, Bekymringer
Sorger og Ærgrelser	Sorg, Ærgelse
Sorger og Stræv	Sorg, Overanstrængelse
Sorger, Ærgrelser og andre Sindsindtryk	Sorg, Ærgelse, Nervechok
Sovesyke	Søvnløshed

Table 4: Terms harmonised *(continued)*

Before: 1455 unique categories	After: 705 unique categories
Sovnløshed	Søvnløshed
Søvnløshed	Søvnløshed
Søvnløshed, Nattevaagen	Søvnløshed
Søvnløshet	Søvnløshed
Søvnløslied	Søvnløshed
Spastisk spinalparalyse	Lammelse
Spedalskhed	Leprosi
Spondylit	Rygsmerte
Srog, haardt Arbeide og Trauma capitis	Sorg, Overanstrængelse, Hjernerystelse
Stærk Hede paa Hovedet (i et Bageri)	Solstik
Stamhet	Talehæmmet
Sterke inntiyk; nervesjokk	Nervechok
Sterke inntryk; nervesjokk	Nervechok
Sterke inntrykk, nervesjokk	Nervechok
Stød (Trauma) paa Hovedet	Hjernerystelse
Stød eller Beskadigelse af Hovedet	Hjernerystelse
Stod paa Hovedet	Hjernerystelse
Stød paa Hovedet	Hjernerystelse
Stød paa Hovedet og Ærgrelser	Hjernerystelse, Ærgelse
Stød paa Hovedet og Blodstigning til Hovedet	Hjernerystelse, Blodstigning til Hovedet
Stød paa Hovedet og Drik	Hjernerystelse, Drik
Stød paa Hovedet og Drik og Selvbesmittelse	Hjernerystelse, Drik, Masturbation
Stød paa Hovedet og legemlig Sygdom	Hjernerystelse, Fysisk Sygdom
Stød paa Hovedet og Ryggen	Hjernerystelse, Rygsmerte
Stød paa Hovedet og Sindsindtryk	Hjernerystelse, Nervechok
Stød paa Hovedet og Syphilis	Hjernerystelse, Syphilis
Stød paa Hovedet, Brud af Hjerneskallen, Hjernerystelse	Hjernerystelse, Hjerneskallebrud
Stød paa Hovedet, legemlig Sygdom (Syphilis) og Imbecillitet	Hjernerystelse, Syphilis, Mental Handicap
Stød, Trauma paa Hovedet	Hjernerystelse
Støt eller slag på hodet	Hjernerystelse
Straf for fødsel i dølgsmaal	Fængselsophold
Strictura urethræ	Urinorgansygdom
Struma	Hyperthyreose
Sukkersyge	Diabetes
Sult	Underernæring
Suppressio mensium	Menorrhagi
Svækkelse	Svækkelse
Svækkelse efter Puerperium	Puerperium
Svækkelse efter tidligere Sindssygdom	Tidligere Sindssygdom

Table 4: Terms harmonised *(continued)*

Before: 1455 unique categories	After: 705 unique categories
Svækkelse og Overanstrængelse	Svækkelse, Overanstrængelse
Svækkelse, transformeret Hysteri, aandelig Overanstrængelse	Svækkelse, Hysteri, Overanstrængelse
Svækkelsestilstand	Svækkelse
Svækkelsestilstande	Svækkelse
Svage Aandsevner	Mental Handicap
Svakhet i barnealderen	Udviklingshæmmet
Svangerskab	Graviditet
Svangerskab og Degeneration	Graviditet, Mental Handicap
Svangerskab og Familiesorg	Graviditet, Familiesorg
Svangerskap	Graviditet
Svindsot	Tuberkulose
Svulst paa Halsen	Svulst paa Halsen
Syfilis	Syphilis
Syfilis og Sindsindtryk	Syphilis, Sindsindtryk
Sygdom	Fysisk Sygdom
Sygdom (Feber, Phlegmone)	Infektionssygdom, Phlegmone
Sygdom i barselseng	Puerperium
Sygdom i halsen	Halssygdom
Sygdom i Urinorganerne	Urinorgansygdom
Sygdom, indre	Psykisk Sygdom
Sygdom, legemlig	Fysisk Sygdom
Sygdom, legemlig og Sindsindtryk	Fysisk Sygdom, Nervechok
Sygdom, legemlig og Stød paa Hovedet	Fysisk Sygdom, Hjernerystelse
Sygdom, ydre og Vold	Fysisk Sygdom, Vold
Sygdommenes art ikke opgit	UNKNOWN
Sygdommens art ikke opgit	UNKNOWN
Sygdommens art ikke opgitt	UNKNOWN
Sypbilis og Selvbesmittelse	Syphilis, Masturbation
Syphilidophobi	Syphilidophobi
Syphilis	Syphilis
Syphilis og Ærgrelse	Syphilis, Ærgelse
Syphilis og Barselseng	Syphilis, Puerperium
Syphilis og Drik	Syphilis, Drik
Syphilis og Græmmelse	Syphilis, Græmmelse
Syphilis og Hjerneslag	Syphilis, Slagtilfælde
Syphilis og Hysteri	Syphilis, Hysteri
Syphilis og langvarig Diegivning	Syphilis, Diegivning
Syphilis og Næringssorg	Syphilis, Fattigdom
Syphilis og økonomiske Bekymringer	Syphilis, Fattigdom

Table 4: Terms harmonised *(continued)*

Before: 1455 unique categories	After: 705 unique categories
Syphilis og Overanstrængelse	Syphilis, Overanstrængelse
Syphilis og religiøse Indflydelser	Syphilis, Religiøse Indflydelser
Syphilis og Rygmarvssygdom	Syphilis, Rygmarvssygdom
Syphilis og Selvbesmittelse	Syphilis, Masturbation
Syphilis og Sindslidelse	Syphilis, Sindslidelser
Syphilis og Sorg	Syphilis, Sorg
Syphilis og Stød paa Hovedet	Syphilis, Hjernerystelse
Syphilis og Udsvævelser	Syphilis, Seksualitet
Syphilis, Overanstrængelse og Commotio cerebri	Syphilis, Overanstrængelse, Hjernerystelse
Talrige Fødsler	Talrige Fødsler
Temor cetebri	Hjernesvulst
Tendovaginit	Betændelse i Haanden
Thyreotoxikose	Hyperthyreose
Tidligere Anfald	Tidligere Sindssygdom
Tidligere Anfald af Sindssygdom	Tidligere Sindssygdom
Tidligere Anfald og Sindsindtryk	Tidligere Sindssygdom, Nervechok
Tidligere hjernebetændelse	Hjernebetændelse
Tidligere sindssygdom	Tidligere Sindssygdom
Tidligere Sindssygdom	Tidligere Sindssygdom
Tidligere Sindssygdom og Drik	Tidligere Sindssygdom, Drik
Tidligere Sindssygdom og Selvbesmittelse	Tidligere Sindssygdom, Masturbation
Tidligere Sindssygdom og smertelig Sindsindtryk	Tidligere Sindssygdom, Nervechok
Tidligere Sindssygdom og smertelige Indtryk	Tidligere Sindssygdom, Nervechok
Til observation	Observation
Til observation indlagte	Observation
Til Observation indlagte	Observation
Til Observation var indlagc	Observation
Til Observation var indlagt	Observation
tillige arveligt Anlæg	Arv
Tobaksforgiftning	Misbrug af Tobak
Torpedering og forlis	Ulykke
Trauma	Ulykke
Trauma capitis	Hjernerystelse
Trauma testis med Kastration	Operation
Tuberculosis vertebrarum	Tuberkulose
Tuberkuløs Ledbetændelse og Influenza	Tuberkulose, Influenza
Tuberkulose	Tuberkulose
Tuberkulose og Sindsindtryk	Tuberkulose, Sindsindtryk
Tumor abdominis	Kræft

Table 4: Terms harmonised *(continued)*

Before: 1455 unique categories	After: 705 unique categories
Tumor cerebri	Hjernesvulst
Tunghørt	Hørehæmmet
Tunghørthet, døvhet	Hørehæmmet
Tyfoidfeber	Typhoidfeber
Tyfus	Typhoidfeber
Typhoidfeber	Typhoidfeber
Typhoidfeber, religiøse Indflydelser, Overanstrængelse	Typhoidfeber, Religiøse Indflydelser, Overanstrængelse
Udskeielser	Livsstil
Udskeielser og Fængselsstraf	Livsstil, Fængselsophold
Udslagssygdom, akut	Rosen
Udsvævelser	Seksualitet
Udsvævelser og smertelige Sindsindtryk	Seksualitet, Nervechok
Udsvævende Levnet	Seksualitet
Udsvævende Liv	Seksualitet
Udtæring (Marasmus)	Marasmus
Uheldige Familieforhold	Familiesorg
Ulcera cruris	Skinnebenssår
Ulcus corneæ	Øienbetændelse
Ulykkelig Familieforhold	Familiesorg
Ulykkelig Kjærlighed	Kærlighedssorg
Ulykkelig Kjærlighed eller Ægteskab	Kærlighedssorg
Ulykkelig Kjærlighed og Ægteskab	Kærlighedssorg
Ulykkelig Kjærlighed og Familiesorg	Kærlighedssorg, Familiesorg
Ulykkelig Kjærlighed og religiøs Vækkelse	Kærlighedssorg, Religiøse Indflydelser
Ulykkelig Kjærlighed og Selvbesmittelse	Kærlighedssorg, Masturbation
Ulykkelig Kjærlighed, ulykkeligt Ægteskab	Kærlighedssorg
Ulykkelige Familie- eller Kjæresteforhold	Familiesorg, Kærlighedssorg
Ulykkelige Familieforhold	Familiesorg
Ulykkelige forhold, kjærlighet	Kærlighedssorg
Ulykkelige huslige Forhold	Familiesorg
Ulykkeligt Ægteskab	Kærlighedssorg
Ulykkeligt Ægteskab eller Familieforhold	Kærlighedssorg, Familiesorg
Ulykkeligt Ægteskab og Selvbesmittelse	Kærlighedssorg, Masturbation
Ulykkestilfælde	Ulykke
Ulykkestilfælde (Beskadigelse)	Ulykke
Ulykkestilfælde (holdt paa at drukne)	Ulykke
Ulykkestilfælde (solstik)	Solstik
Ulykkestilfælde og legemlig Sygdom	Ulykke, Fysisk Sygdom
Ulykkestilfelle	Ulykke

Table 4: Terms harmonised *(continued)*

Before: 1455 unique categories	After: 705 unique categories
Ulykkestilfelle, trauma	Ulykke
Underernæring	Underernæring
Underlivsbetændelse	Underlivssygdom
Underlivssygdom	Underlivssygdom
Underlivssygdom efter Barselseng	Underlivssygdom
Underlivssygdom og menorrhagi	Underlivssygdom, Menorrhagi
Underlivssygdom og Næringssorg	Underlivssygdom, Fattigdom
Underlivstilfælde	Underlivssygdom
Unknown	UNKNOWN
Uopgit legemlig sygdom	UNKNOWN
Uordentlig liv	Livsstil
Uordentligt Levnet	Livsstil
Uordentligt Liv	Livsstil
Uordentligt og ubehersket Liv, Udsvævelser	Livsstil, Seksualitet
Uræmi, overanstrengelse	Nyresygdom, Overanstrængelse
Usund religiøs påvirkning	Religiøse Indflydelser
Uterinsygdom	Uterinsygdom
Utroskab	Kærlighedssorg
Utskeielser	Livsstil
Vaadeskud	Vaadeskud
Værkefinger	Betændelse i Haanden
Vanførhet	Udviklingshæmmet
Varices med blødninger	Åreknuder
Variolae	Infektionssygdom
Vitia organica cerebri	Hjernefejl
Vitia organica cordis	Hjertefejl
Vitium cordis	Hjertefejl
Vitium organic cerebri	Hjernefejl
Voldtægtsforsøg	Voldtægtsforsøg
Vulneratio	Ulykke
Vulneratio cerebri	Mental Handicap

Figure 3: Sankey diagrams of causes for admissions as we harmonised them

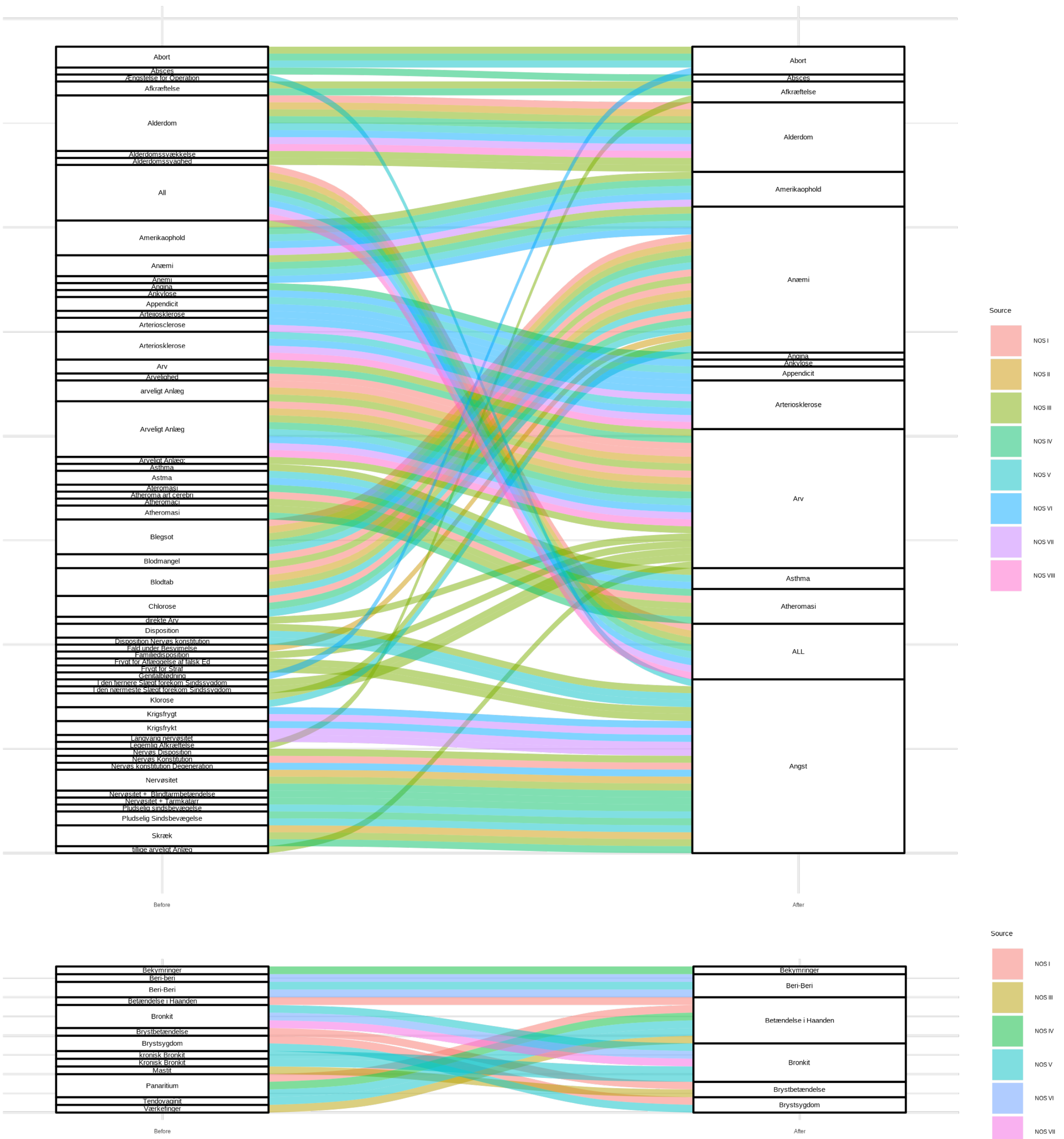

Figure 3 Sankey diagrams of causes for admissions as we harmonised them (*continued)*

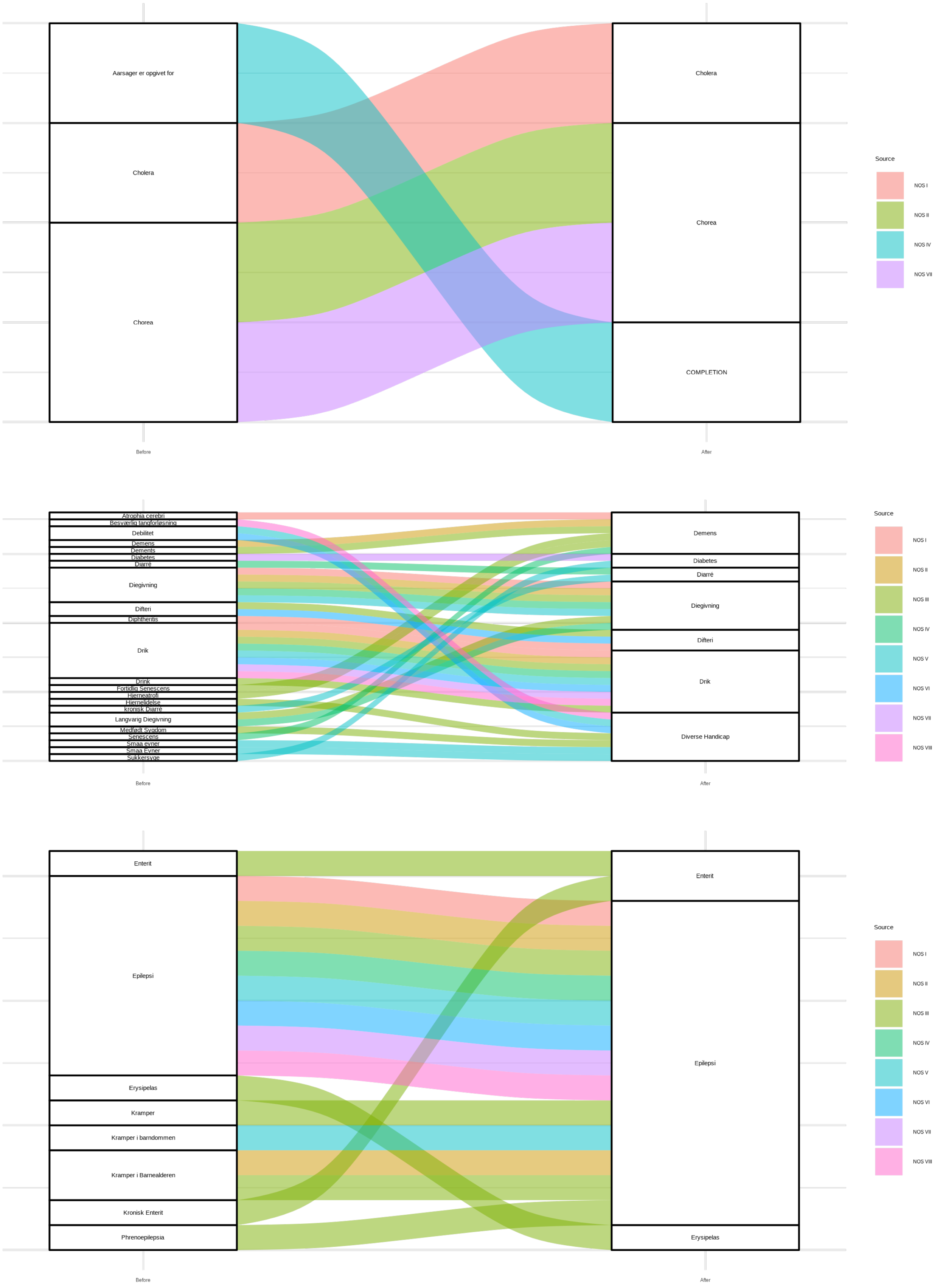

Figure 3 Sankey diagrams of causes for admissions as we harmonised them (*continued)*

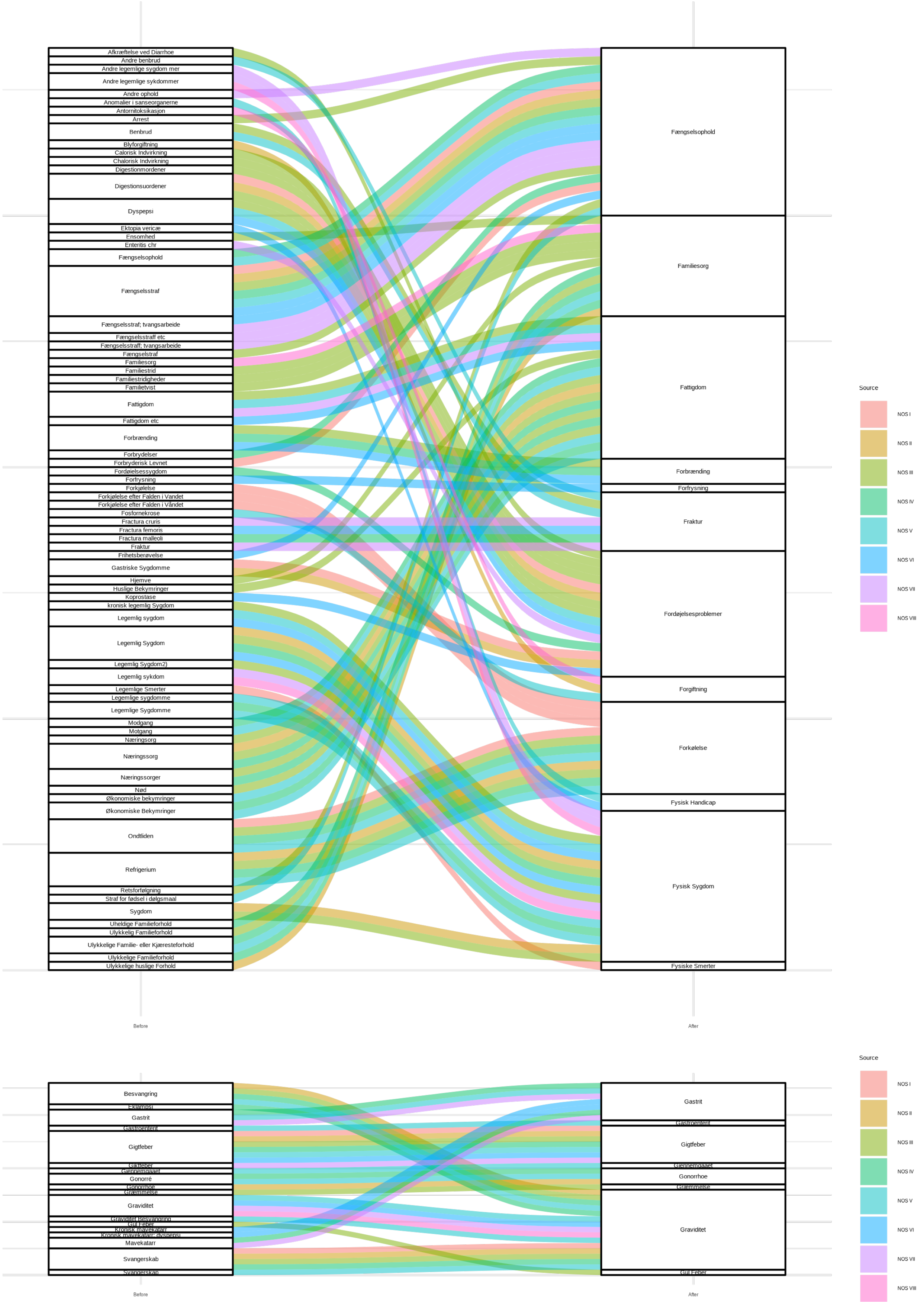

Figure 3 Sankey diagrams of causes for admissions as we harmonised them (*continued)*

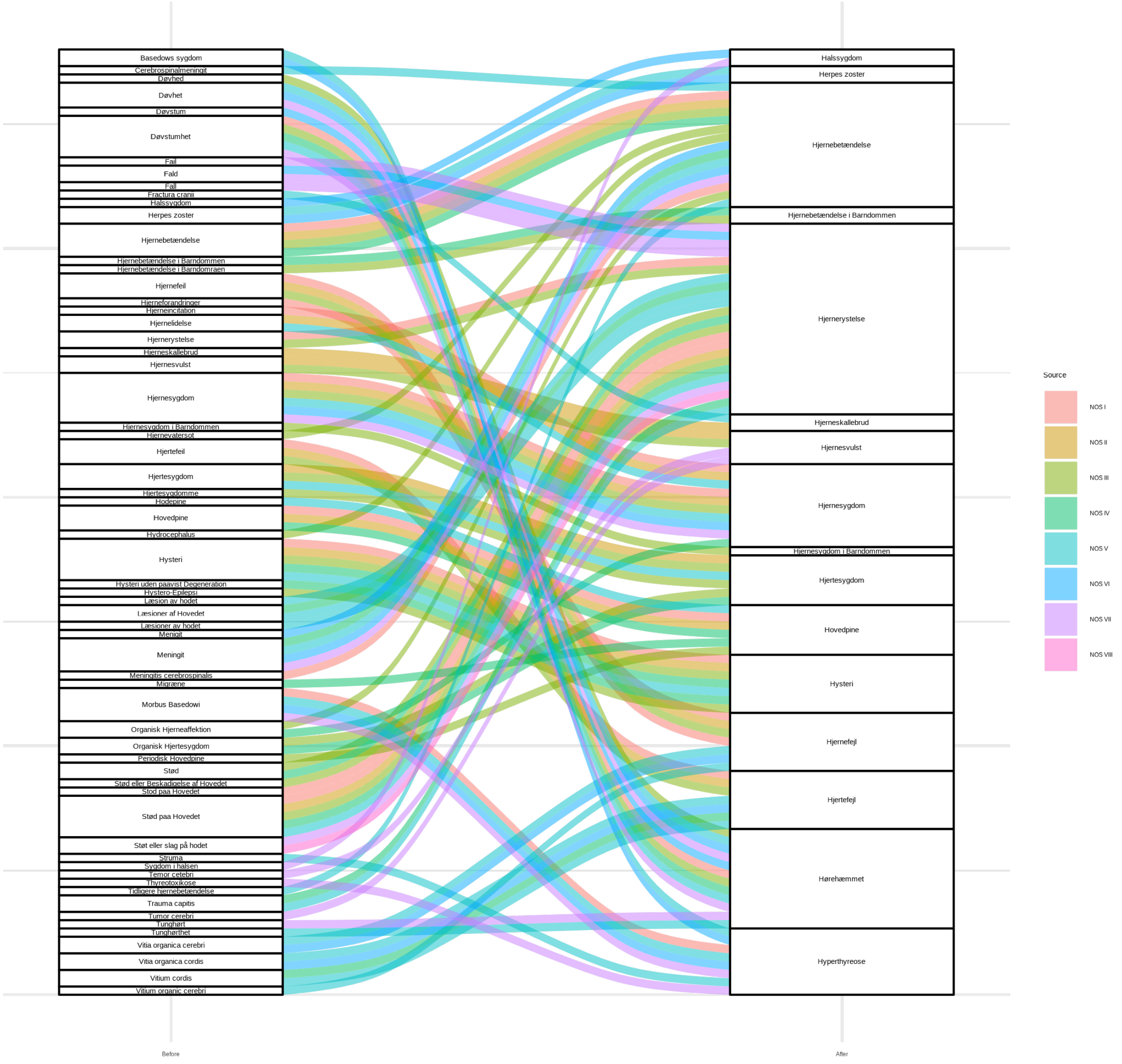

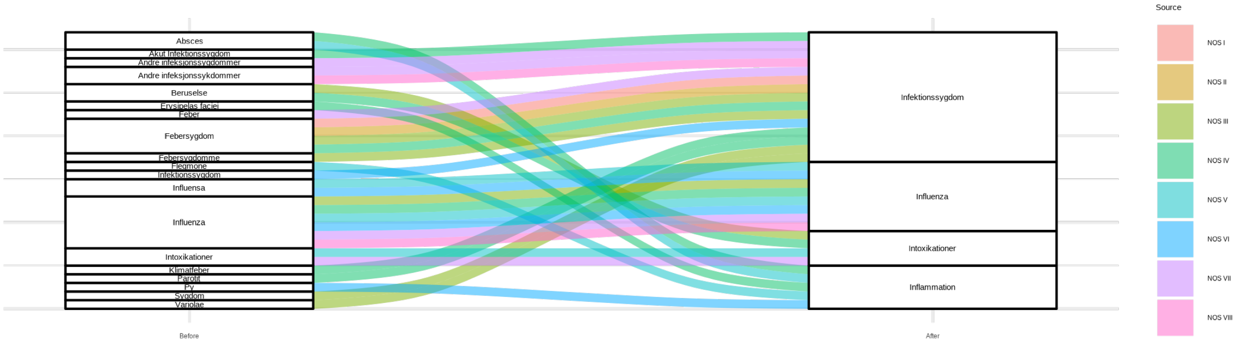

Figure 3 Sankey diagrams of causes for admissions as we harmonised them (*continued)*

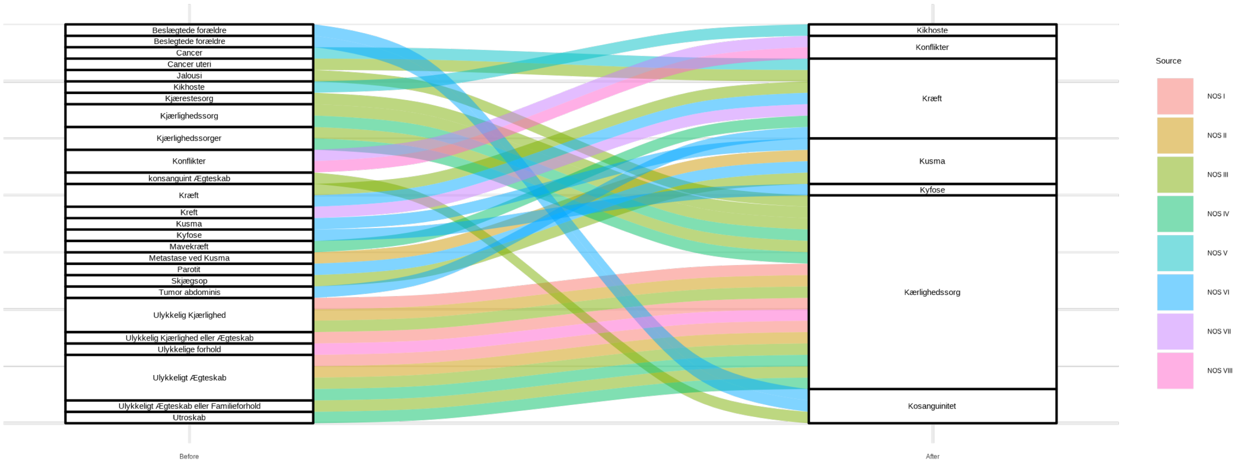

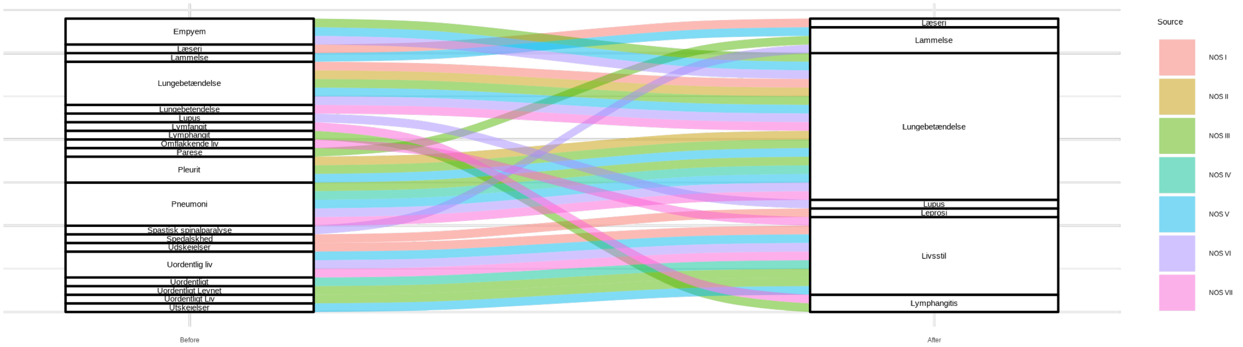

Figure 3 Sankey diagrams of causes for admissions as we harmonised them (*continued)*

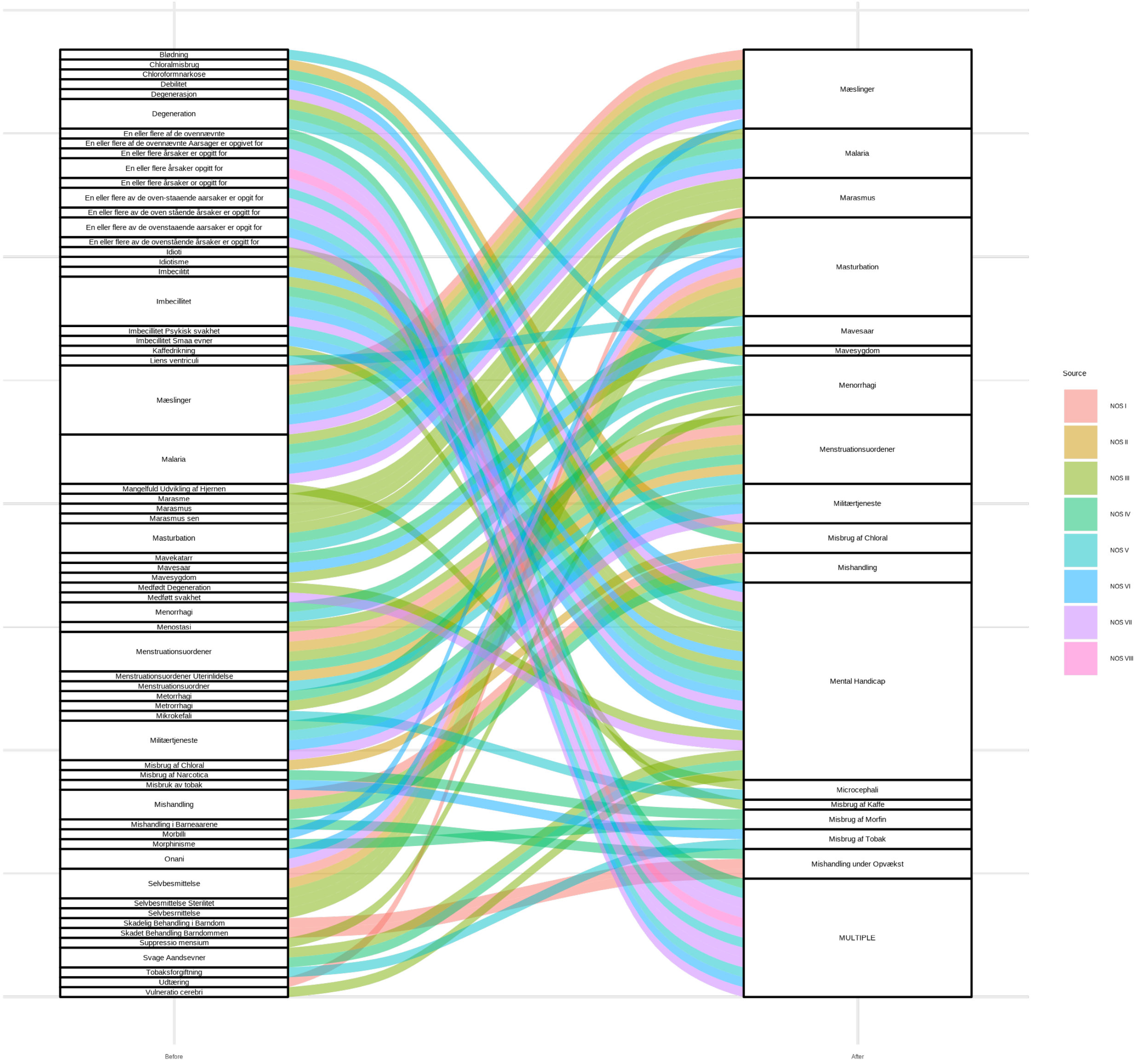

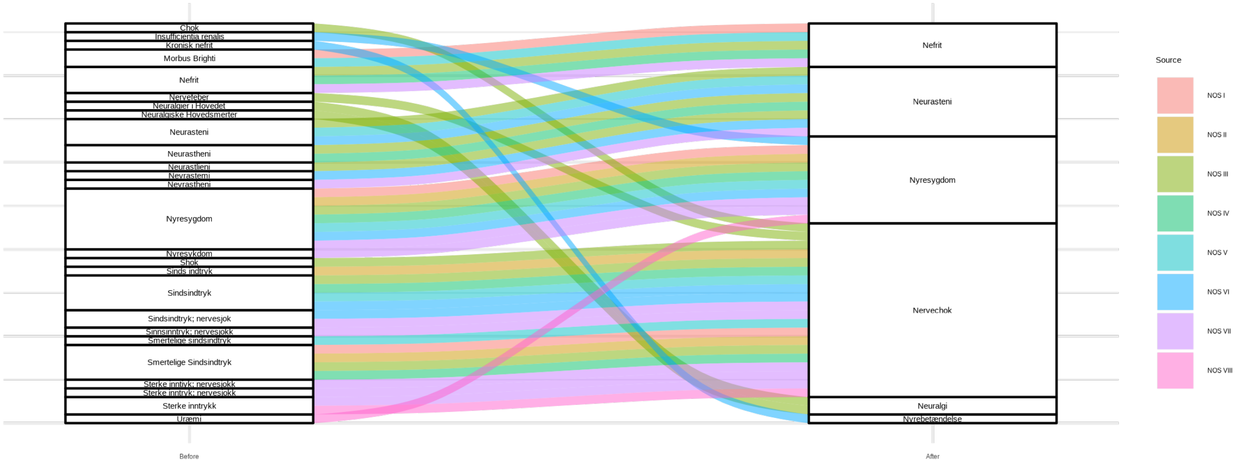

Figure 3 Sankey diagrams of causes for admissions as we harmonised them (*continued)*

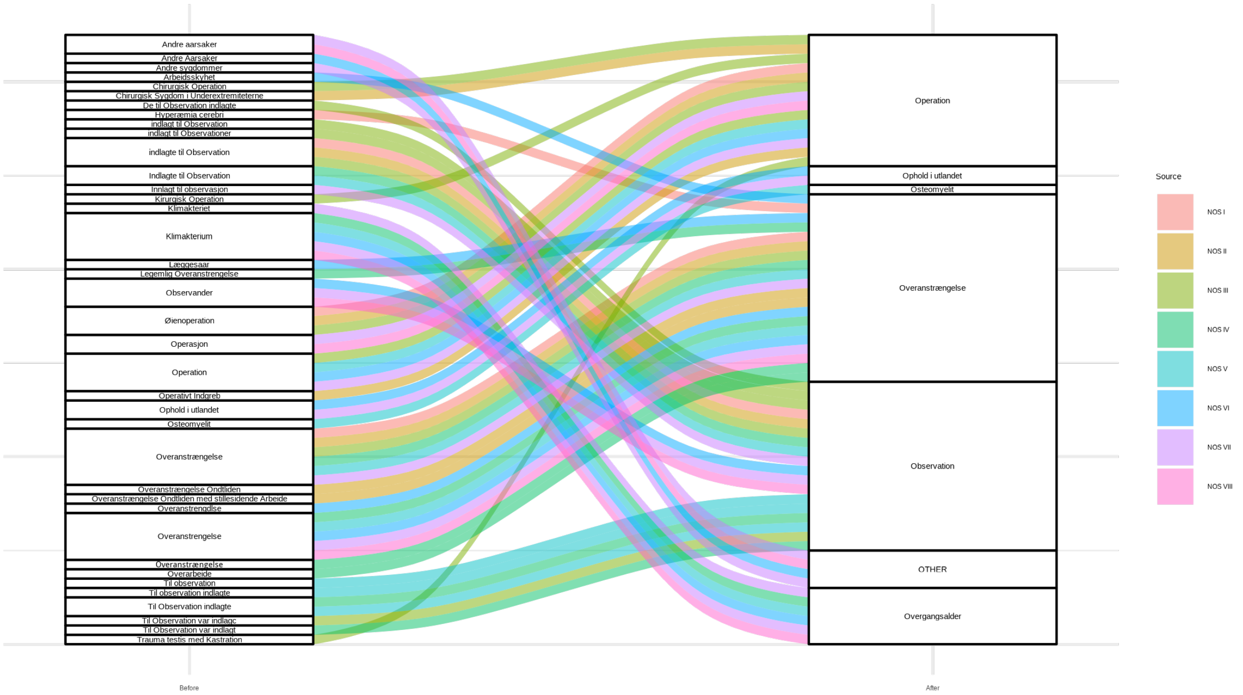

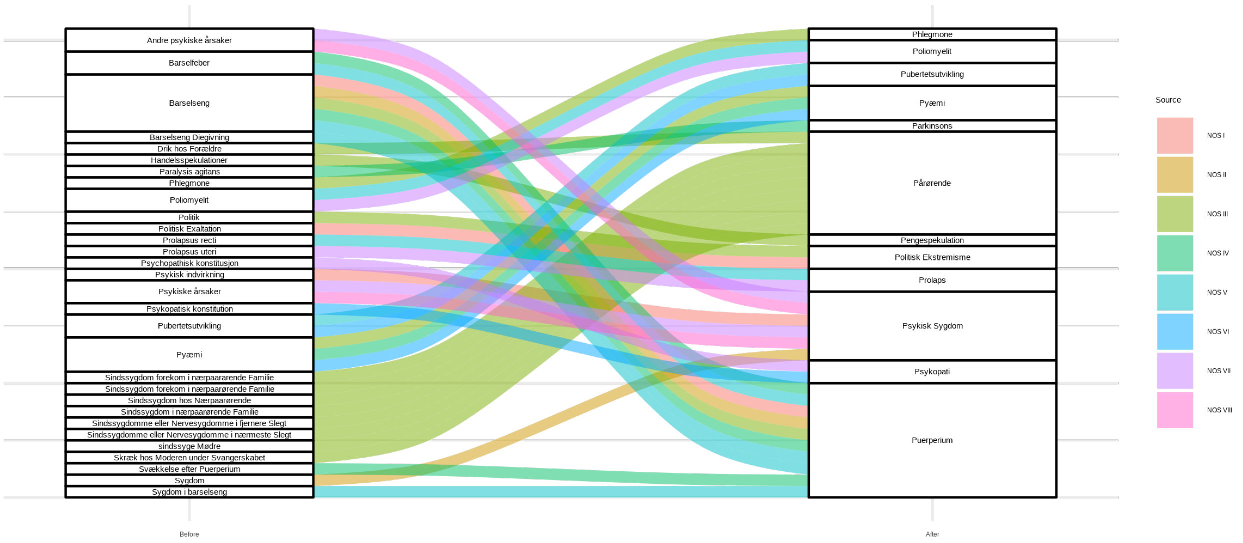

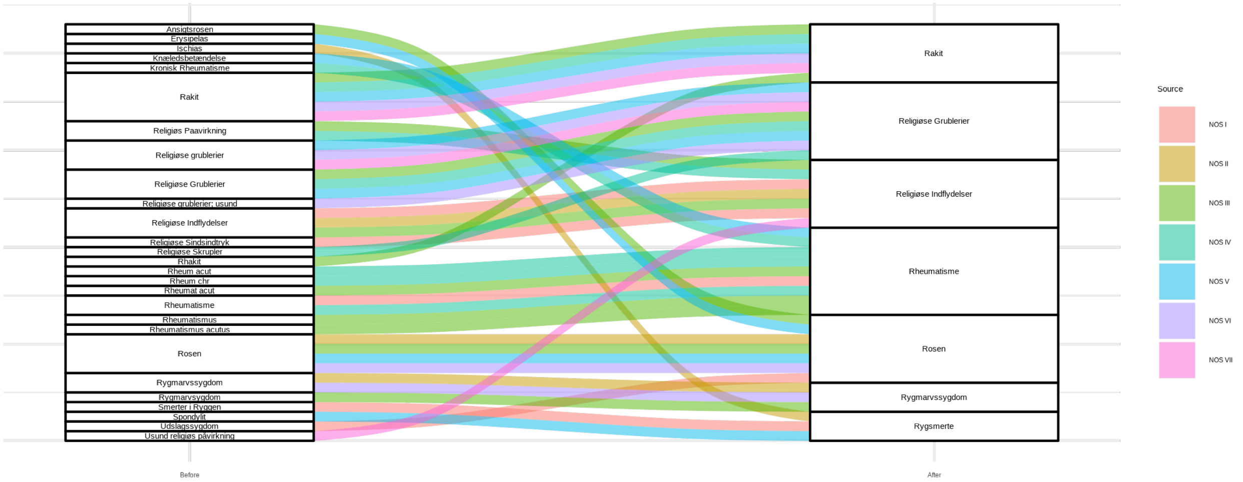

Figure 3 Sankey diagrams of causes for admissions as we harmonised them (*continued)*

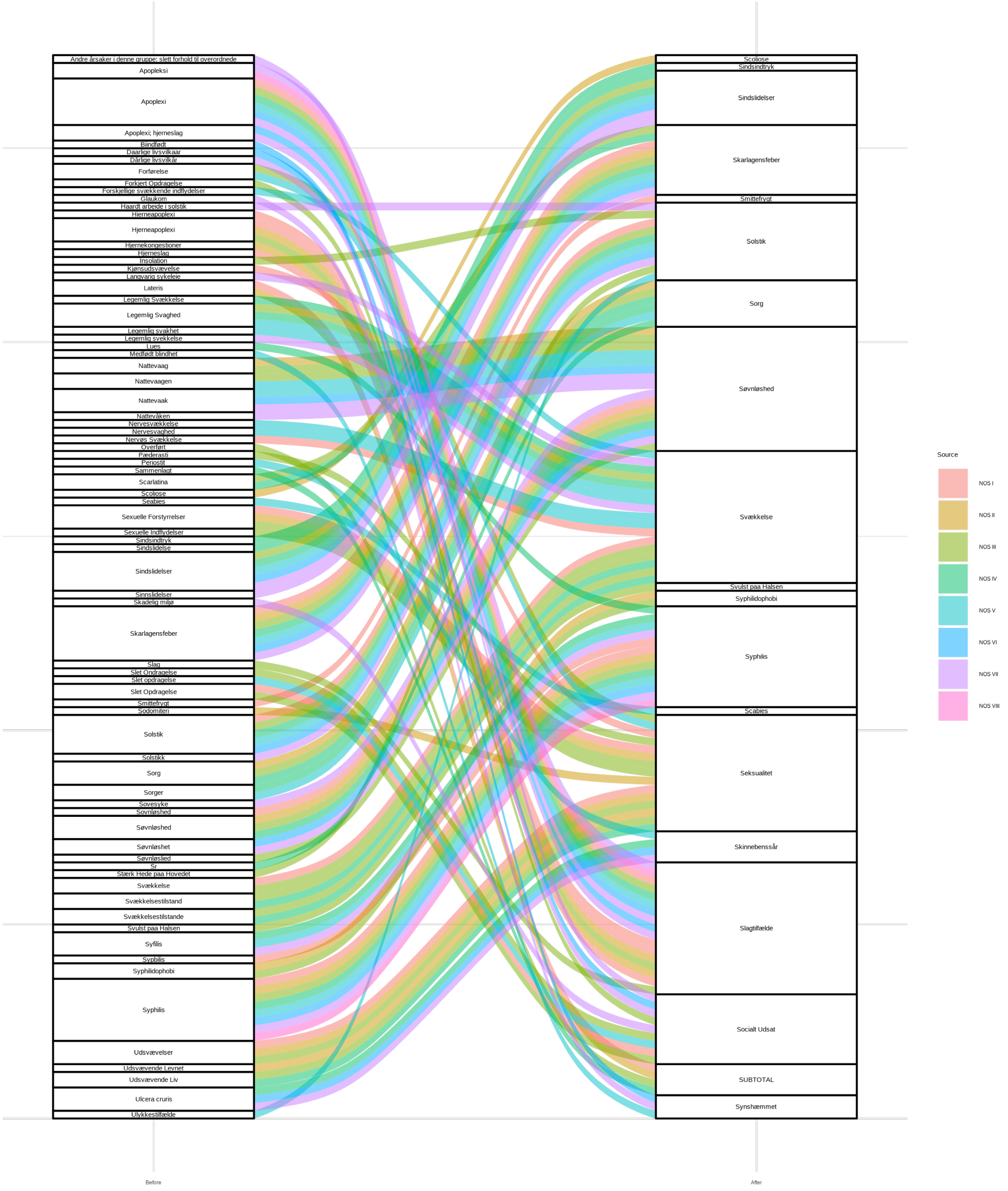

Figure 3 Sankey diagrams of causes for admissions as we harmonised them (*continued)*

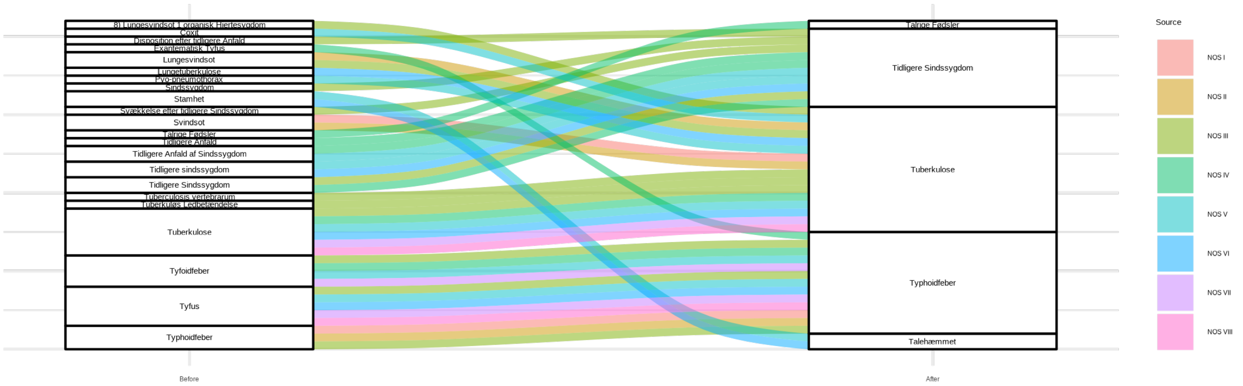

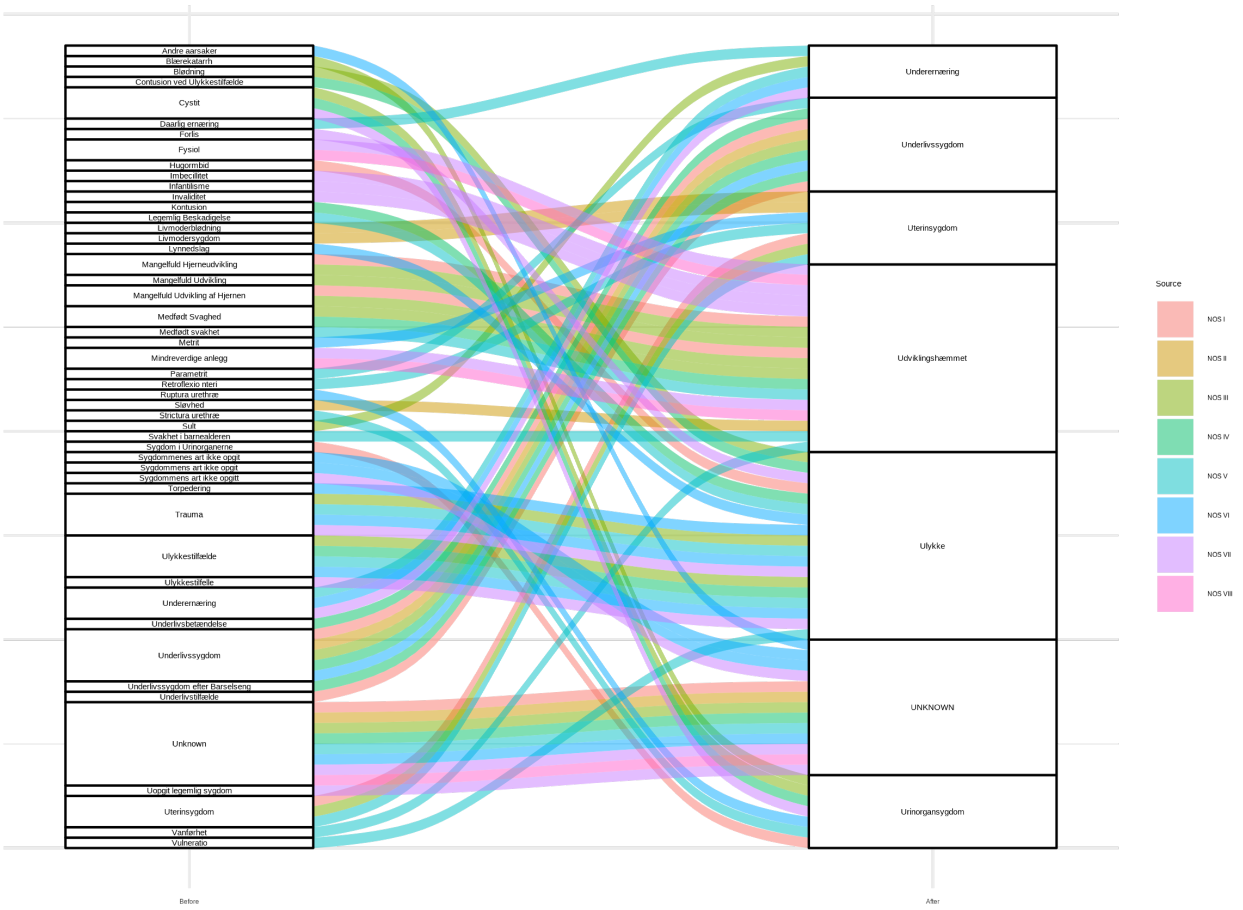

Figure 3 Sankey diagrams of causes for admissions as we harmonised them (*continued)*

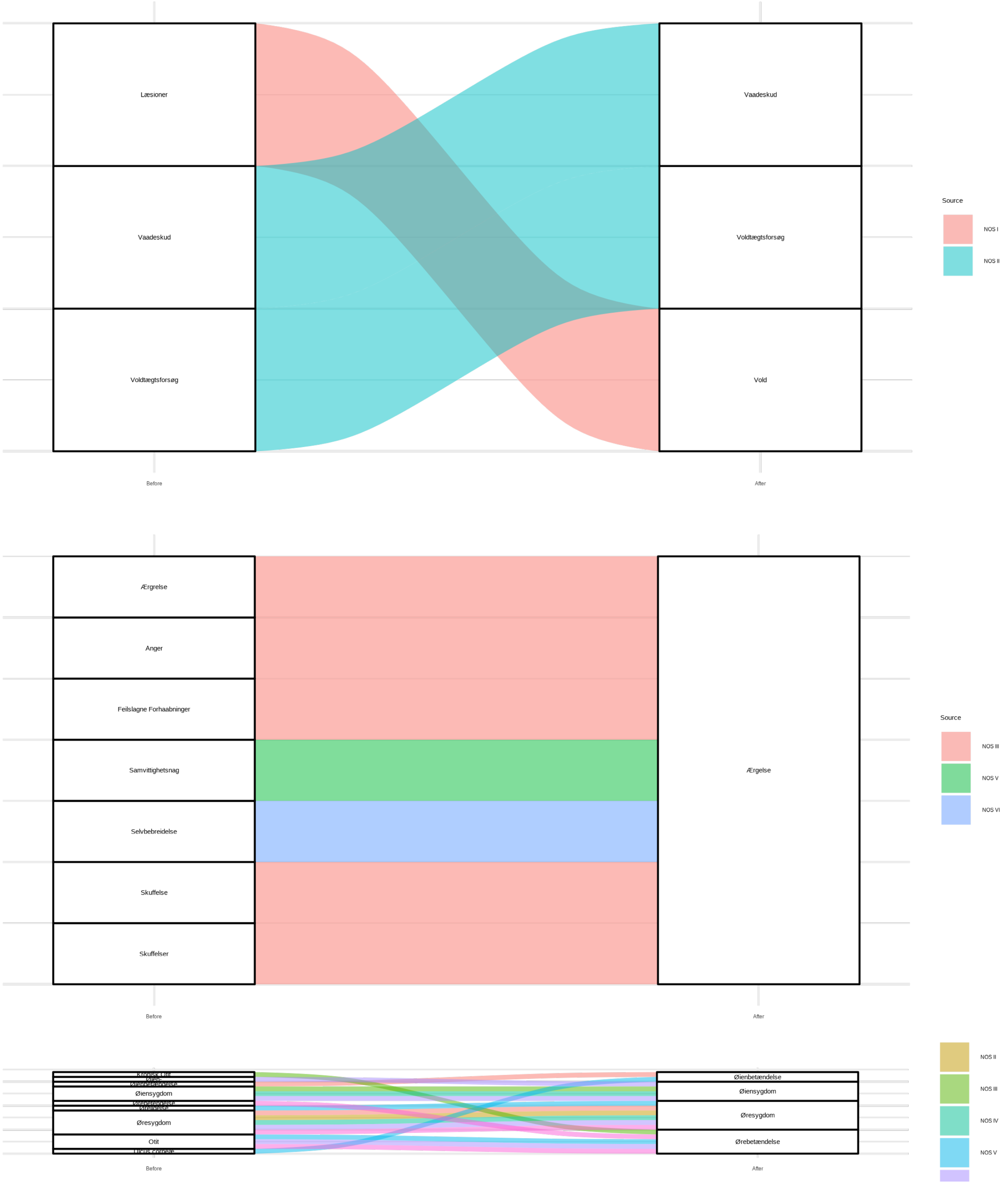

Figure 3 Sankey diagrams of causes for admissions as we harmonised them (*continued)*

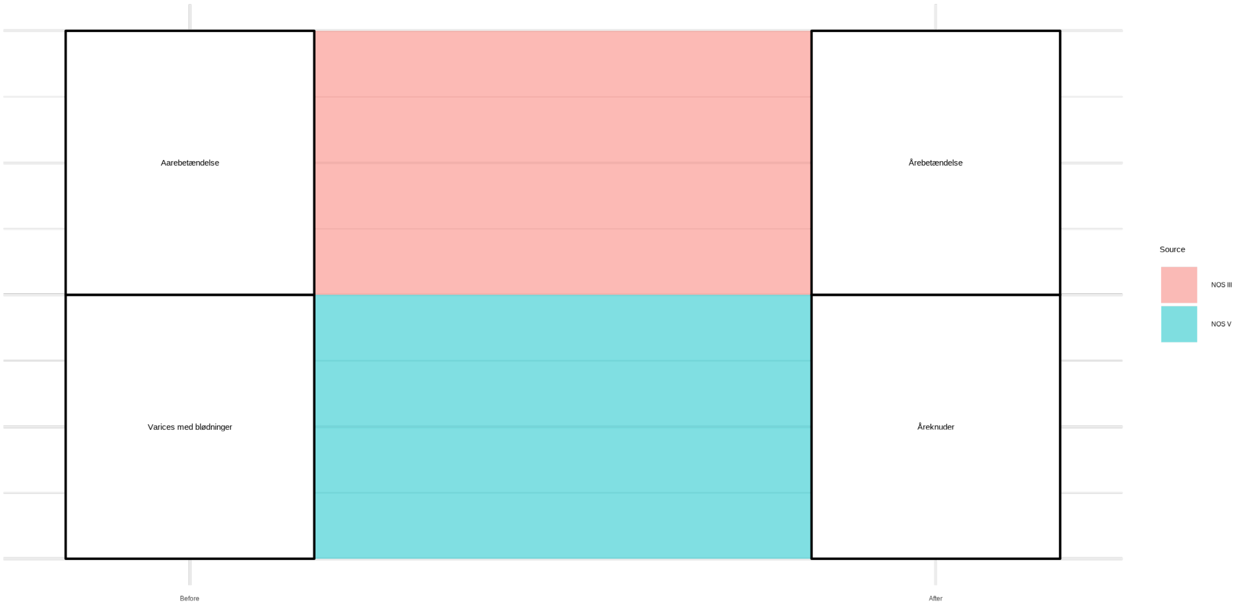

Table 5: Terms harmonised

Primary cause	Causes harmonised	Code	Bertillon	Categorisation
Abort	2	VI	Diseases of genito-urinary system	Women's health
Absces	1			
Ærgelse	7			
Afkræftelse	2			
Alderdom	3	XII	Diseases of old age	Aging
ALL	1			
Amerikaophold	1	XIII	Effects of external causes	
Anæmi	8	III	Diseases of circulatory system	Women's health
Angst	17			
Ankylose	1			
Appendicit	1			
Årebetændelse	1			
Åreknuder	1			
Arteriosklerose	3			
Arv	10			Hereditary
Asthma	2	IV	Diseases of respiratory system	
Atheromasi	4			
Bekymringer	1			
Beri-Beri	2			
Betændelse i Haanden	4			
Bronkit	3			
Brystbetændelse	2			
Brystsygdom	1			
Cholera	1	I-A	Epidemic diseases	Bacterial infection
Chorea	1			
COMPLETION	1			
Demens	6	XII	Diseases of old age	
Diabetes	2	I-B	Other general diseases	
Diarré	2			
Diegivning	2	VII	Puerperal diseases	Women's health
Difteri	2	I-A	Epidemic diseases	Bacterial infection
Diverse Handicap	6			
Drik	2	XIII	Effects of external causes	Substance use
Enterit	2			

Table 5: Terms harmonised *(continued)*

Primary cause	Causes harmonised	Code	Bertillon	Categorisation
Epilepsi	5			
Erysipelas	1			
Fængselsophold	13	XIII	Effects of external causes	Crime
Familiesorg	11	XIII	Effects of external causes	Relationships
Fattigdom	11	XIII	Effects of external causes	Poverty
Forbrænding	1			
Fordøjelsesproblemer	10	V	Diseases of digestive system	
Forfrysning	1			
Forgiftning	3			
Forkølelse	5	I-A	Epidemic diseases	Viral infection
Fraktur	6			
Fysisk Handicap	2			
Fysisk Sygdom	10			
Fysiske Smerter	1			
Gastrit	4	V	Diseases of digestive system	
Gastroenterit	1	V	Diseases of digestive system	
Gigtfeber	2	I-A	Epidemic diseases	Bacterial infection
Gjennemgaaet	1			
Gonorrhoe	2	I-A	Epidemic diseases	Bacterial infection
Græmmelse	1			
Graviditet	6	VII	Puerperal diseases	Women’s health
Gul Feber	1	I-A	Epidemic diseases	Viral infection
Halssygdom	2			
Herpes zoster	1	I-A	Epidemic diseases	Viral infection
Hjernebetændelse	9			Cerebral
Hjernebetændelse i Barndommen	2			Cerebral
Hjernefejl	4			Cerebral
Hjernerystelse	13			Cerebral
Hjerneskallebrud	2			Cerebral
Hjernesvulst	3			Cerebral
Hjernesygdom	4			Cerebral
Hjernesygdom i Barndommen	1	XI	Diseases of early infancy	Cerebral
Hjertefejl	3			Cardiovascular
Hjertesygdom	3			Cardiovascular
Hørehæmmet	6			
Hovedpine	4			
Hyperthyreose	4			Hormonal
Hysteri	3			Women’s health
Infektionssygdom	11	I-A	Epidemic diseases	
Inflammation	4	I-A	Epidemic diseases	
Influenza	2	I-A	Epidemic diseases	Viral infection
Intoxikationer	2	XIII	Effects of external causes	Substance use
Kærlighedssorg	10	XIII	Effects of external causes	Relationships
Kikhoste	1	I-A	Epidemic diseases	
Kosanguinitet	3			
Kræft	6			
Kusma	4	I-A	Epidemic diseases	Viral infection
Kyfose	1			
Læseri	1			
Lammelse	3			
Leprosi	1	I-A	Epidemic diseases	Bacterial infection
Livsstil	7			
Lungebetændelse	5			
Lupus	1			
Lymphangitis	2	I-A	Epidemic diseases	Bacterial infection
Mæslinger	2	I-A	Epidemic diseases	Viral infection
Malaria	1	I-A	Epidemic diseases	Bacterial infection
Marasmus	4			
Masturbation	5			
Mavesaar	3	V	Diseases of digestive system	
Mavesygdom	1	V	Diseases of digestive system	

Table 5: Terms harmonised *(continued)*

Primary cause	Causes harmonised	Code	Bertillon	Categorisation
Menorrhagi	5	VI	Diseases of genito-urinary system	Women's health
Menstruationsuordener	4	VI	Diseases of genito-urinary system	Women's health
Mental Handicap	13			
Microcephali	2	X	Malformations	Developmental
Militærtjeneste	1			
Misbrug af Chloral	3	XIII	Effects of external causes	Substance use
Misbrug af Kaffe	1	XIII	Effects of external causes	Substance use
Misbrug af Morfin	2	XIII	Effects of external causes	Substance use
Misbrug af Tobak	2	XIII	Effects of external causes	Substance use
Mishandling	1			Abuse
Mishandling under Opvækst	3	XIII	Effects of external causes	Abuse
MULTIPLE	9			
Nefrit	2			Renal
Nervechok	12			
Neuralgi	2	II	Diseases of nervous system and sense organs	
Neurasteni	5			
Nyrebetændelse	1	VI	Diseases of genito-urinary system	Renal
Nyresygdom	4	VI	Diseases of genito-urinary system	Renal
Observation	12			
Øienbetændelse	2	II	Diseases of nervous system and sense organs	Visual
Øiensygdom	2	II	Diseases of nervous system and sense organs	Visual
Operation	8			Surgical
Ophold i utlandet	1	XIII	Effects of external causes	
Ørebetændelse	3	II	Diseases of nervous system and sense organs	Aural
Øresygdom	2	II	Diseases of nervous system and sense organs	Aural
Osteomyelit	1	IX	Diseases of locomotor organs	
OTHER	3			
Overanstrængelse	11			
Overgangsalder	2	VI	Diseases of genito-urinary system	Women's health
Parkinsons	1			
Pårørende	9			
Pengespekulation	1	XIII	Effects of external causes	
Phlegmone	1			
Poliomyelit	1	I-A	Epidemic diseases	
Politisk Ekstremisme	2	XIII	Effects of external causes	
Prolaps	2			
Psykisk Sygdom	4			
Psykopati	2			
Pubertetsutvikling	1	VI	Diseases of genito-urinary system	Sexual health
Puerperium	5	VII	Puerperal diseases	Women's health
Pyæmi	1	I-A	Epidemic diseases	
Rakit	2	XIV	Ill-defined diseases	
Religiøse Grublerier	4	XIII	Effects of external causes	Religion
Religiøse Indflydelser	4	XIII	Effects of external causes	Religion
Rheumatisme	8	IX	Diseases of locomotor organs	
Rosen	4	I-A	Epidemic diseases	
Rygmarvssygdom	2			
Rygsmerte	3			
Scabies	1			
Seksualitet	9			Sexual health
Sindslidelser	3			
Skarlagensfeber	2	I-A	Epidemic diseases	Bacterial infection
Skinnebenssår	2	IX	Diseases of locomotor organs	
Slagtilfælde	8			Cerebral
Smittefrygt	1			
Socialt Udsat	8	XIII	Effects of external causes	

Table 5: Terms harmonised *(continued)*

Primary cause	Causes harmonised	Code	Bertillon	Categorisation
Solstik	6	VIII	Diseases of skin and annexes	
Sorg	3	XIII	Effects of external causes	
Søvnløshed	9	XIV	Ill-defined diseases	
SUBTOTAL	3			
Svækkelse	12			
Svulst paa Halsen	1			
Synshæmmet	3			Visual
Syphilidophobi	1	XIV	Ill-defined diseases	
Syphilis	4	I-A	Epidemic diseases	Bacterial infection
Talehæmmet	1			Oral
Talrige Fødsler	1	VII	Puerperal diseases	Women's health
Tidligere Sindssygdom	7			
Tuberkulose	9	I-A	Epidemic diseases	Bacterial infection
Typhoidfeber	4	I-A	Epidemic diseases	Bacterial infection
Udviklingshæmmet	13	X	Malformations	Developmental
Ulykke	12			Injury
Underernæring	3	V	Diseases of digestive system	Malnutrition
Underlivssygdom	5	VI	Diseases of genito-urinary system	Sexual health
UNKNOWN	6			
Urinorgansygdom	5	VI	Diseases of genito-urinary system	Sexual health
Uterinsygdom	5	VI	Diseases of genito-urinary system	Women's health
Vaadeskud	1	XIII	Effects of external causes	
Voldtægtsforsøg	1	XIII	Effects of external causes	Women's health

2.2 Graphical visualisation of harmonisation

In addition to the Sankey diagrams, we showcase the effects of harmonising the causes (before and after) in Figures 4, 5, and 6.

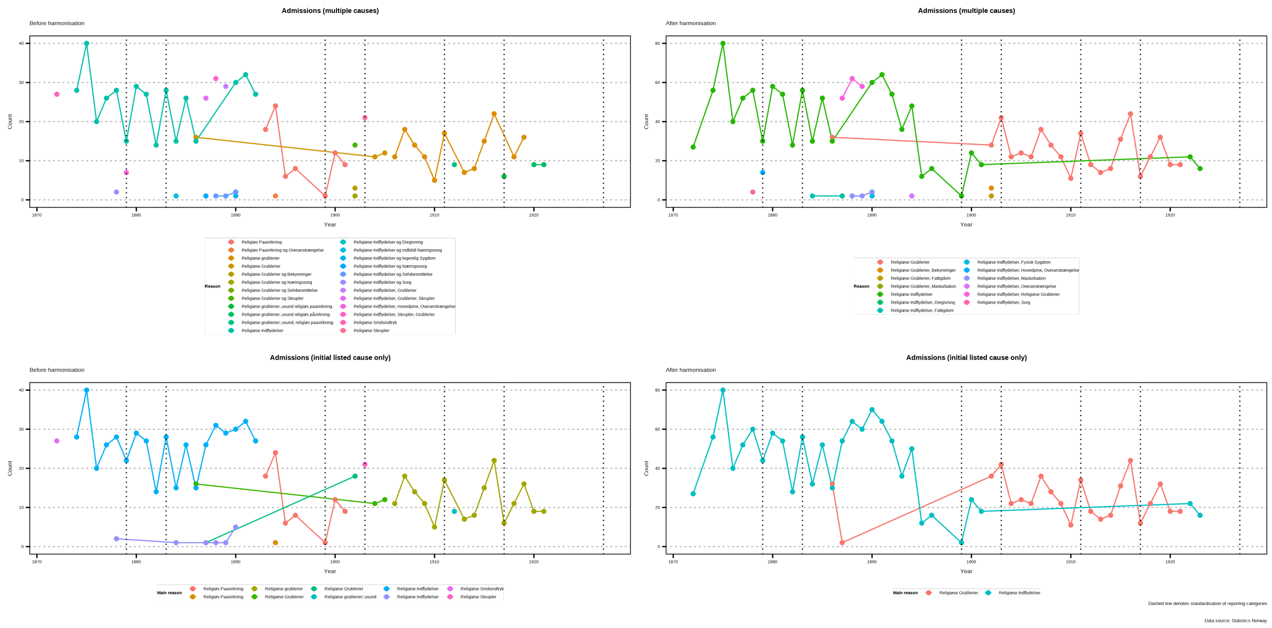

Figure 4: Example of the effect of harmonising terms related to religion

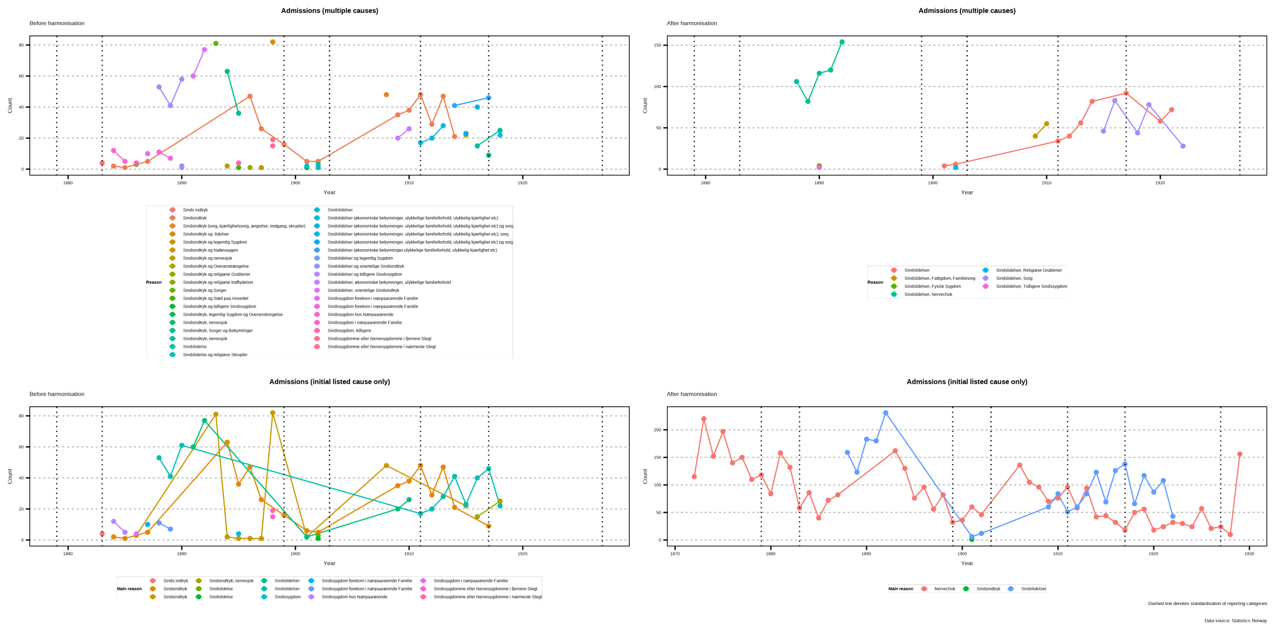

Figure 5: Example of the effect of harmonising terms related to psychological impressions

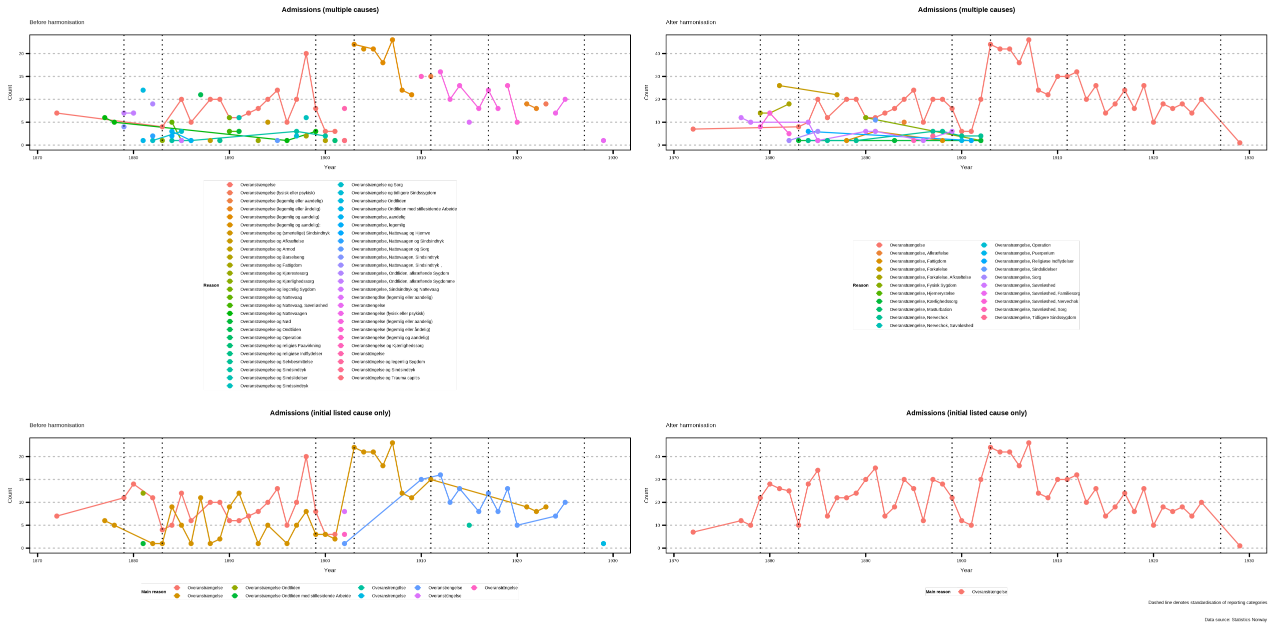

Figure 6: Example of the effect of harmonising terms related to overexertion

3 Appendix – Additional context

The population growth in Norway as given in censuses in 1865, 1875, 1900, 1910, and 1920 shows linear growth (Figure 7). This reflects the epidemiological transition of nineteenth century Norway and suggested "sustained growth" following the terminology of Omran [2005].

We provide Figure 8 that showcases the capacity by facility and Figure 9 which showcases the capacity by facility type. By virtue of having added the contemporary NUTS re-

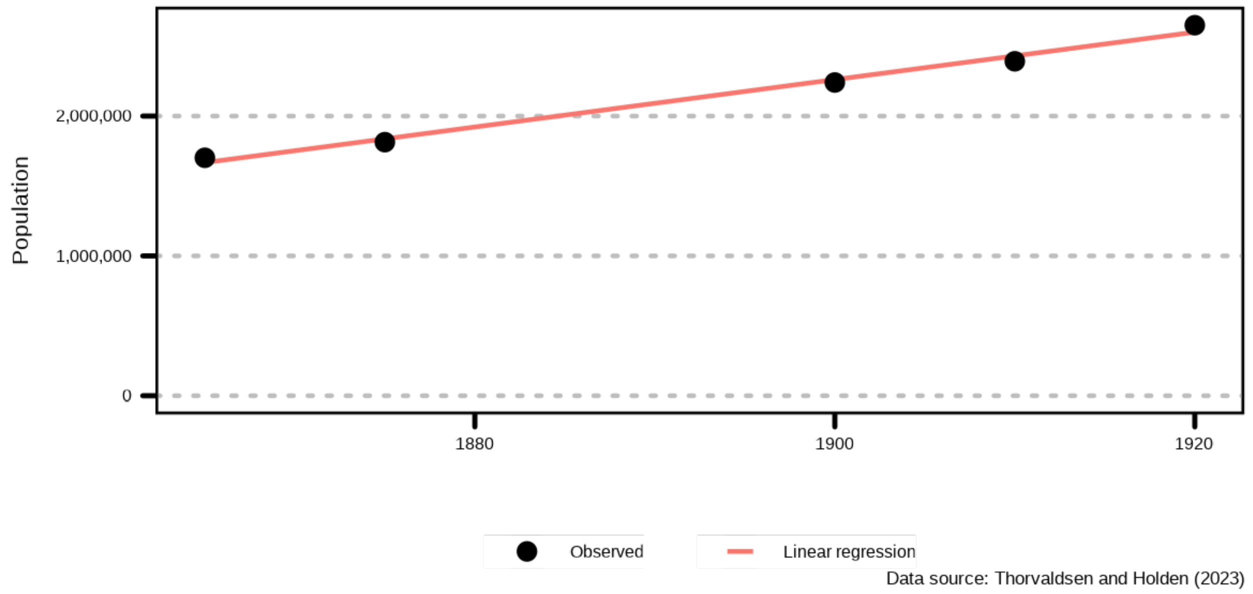

Figure 7: Population census values according to Thorvaldsen and Holden [2023] to provide a sense of the magnitude/burden

gions, we are also able to showcase capacity by region (Figure 10).

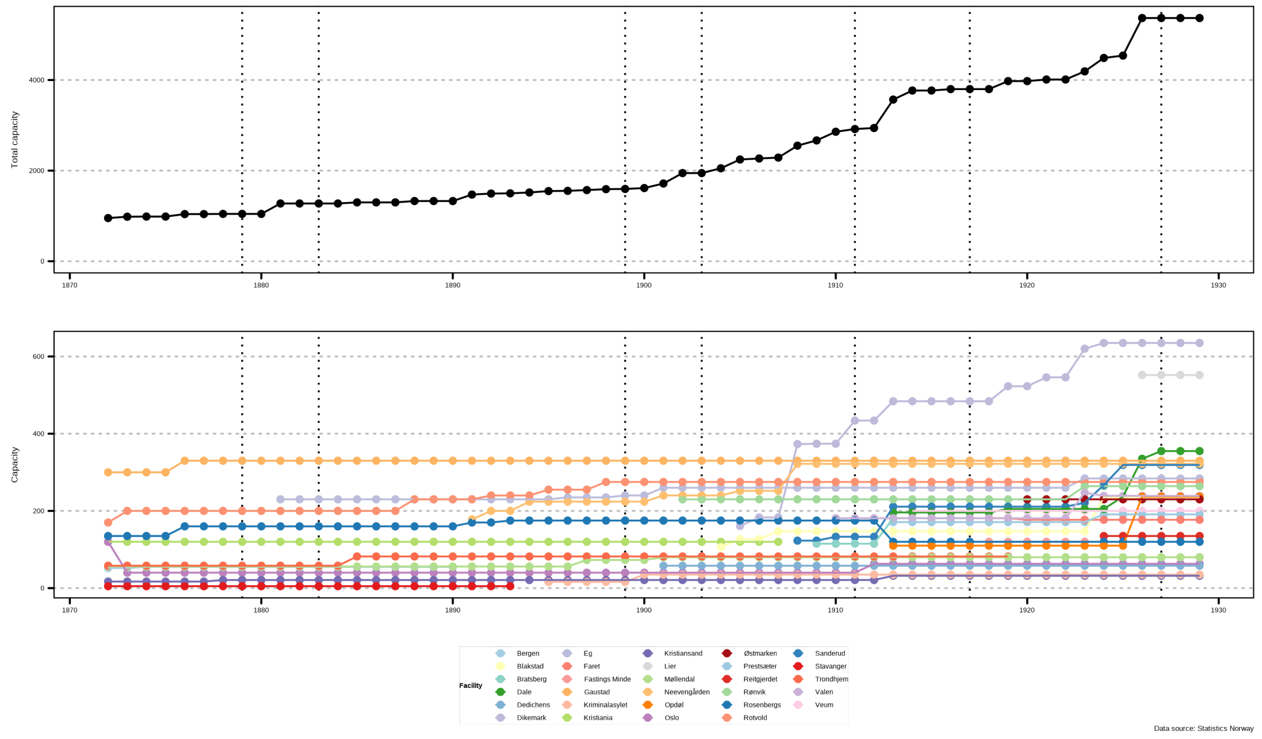

Figure 8: Total and individual capacity at facilities

The mapping of terms used in the comparison with other locations is given in Table 6. It is read right-to-left.

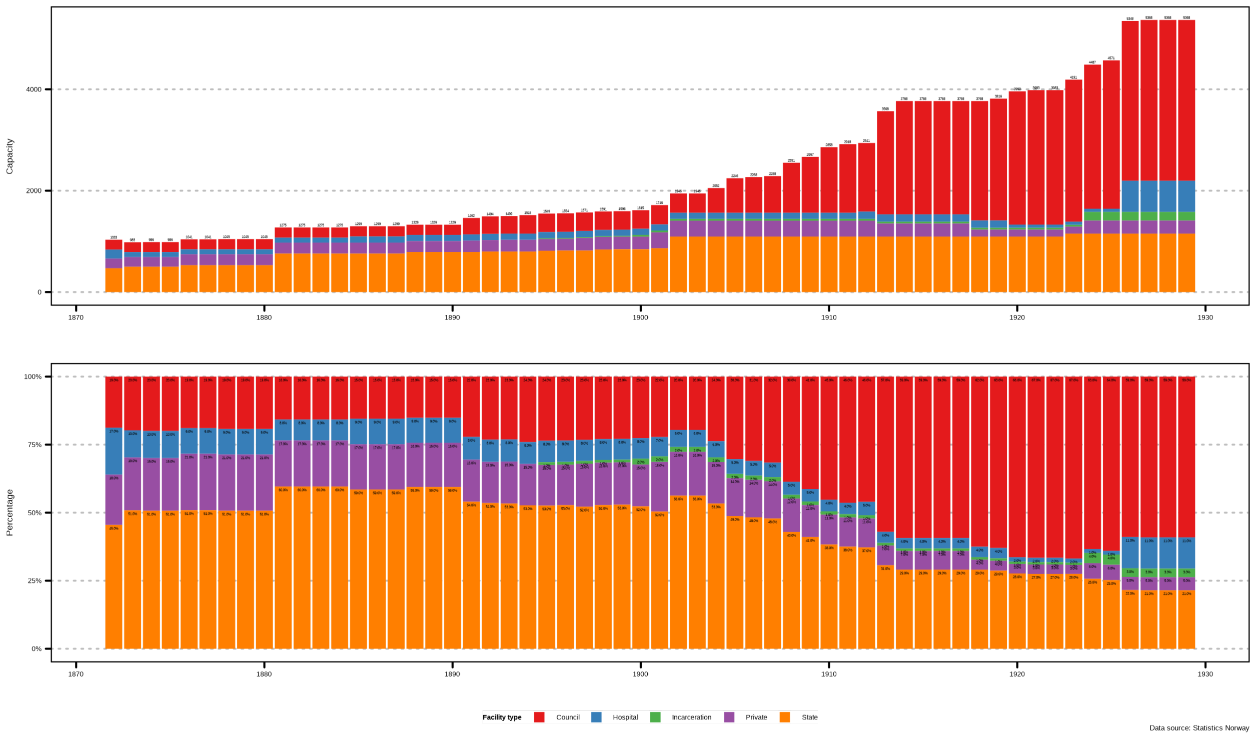

Figure 9: Capacity at facilities stratified by type of facility

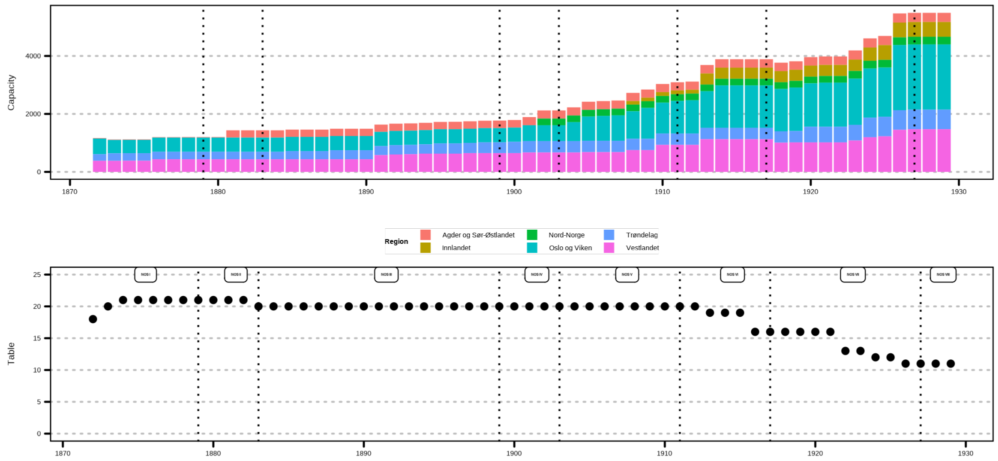

Figure 10: Capacity at as given in the introduction to the reports mapped to NUTS regions (above) and location of the records in the report (below)

Table 6: Mapping of terms used in comparison with other locations

Norway	Oregon	Wales	Sweden
Alderdom	Old age		
Angst	Paranoia	Neurotic disorder	
Angst	Paranoia	Neurotic disorder	Paranoia
Angst	Nervous prostration	Neurotic disorder	
Angst	Nervous prostration	Neurotic disorder	Paranoia
Angst	Fright	Neurotic disorder	
Angst	Fright	Neurotic disorder	Paranoia

Table 6: Mapping of terms used in comparison with other locations *(continued)*

Norway	Oregon	Wales	Sweden
Arteriosklerose		Organic disorder	
Arv	Heredity		
Barselseng	Childbirth		
Bekymringer	Worry		
Demens	Dementia	Dementia	Dementia
Demens	Dementia	Dementia	Insania simplex
Demens	Dementia	Dementia	
Demens	Dementia	Schizophrenia	Dementia
Demens	Dementia	Schizophrenia	Insania simplex
Demens	Dementia	Schizophrenia	
Diverse Handicap	General debility		
Diverse Handicap	General debility		Idiotia
Diverse Handicap	Congenital		
Diverse Handicap	Congenital		Idiotia
Drik		Alcohol/drug related disorder	
Drik		Alcohol/drug related disorder	Alcohol
Drik			Alcohol
Epilepsi	Epilepsy		
Familiesorg	Domestic Trouble		
Forgiftning	Lead poisoning		
Forkølelse	Exposure and sickness		
Forkølelse	Exposure and solitude		
Fysisk Sygdom	Ill health		
Hjernerystelse	Concussion of Brain		
Hjernerystelse	Injury to head		
Hjernesvulst	Brain Softening		
Hysteri			Hysteria
Hysteri, Nevrasteni			Hysteroneurasthenia
Influenza	Influenza		
Kærlighedssorg	Disappointment in Love		
Lammelse	Paralysis		
Livsstil	Dissipation		
Livsstil	Intemperance		
Livsstil, Epilepsi	Intemperance and epilepsy		
Masturbation	Masturbation		
Meningitis	Hydrocephalus		
Meningitis	Spinal meningitis		
Meningitis	Meningitis		
Mental handicap	Idiocy		
Mental Handicap		Mental handicap	
Misbrug af Morfin	Morphine and cocaine		
Misbrug af Morfin	Opium habit		
Misbrug af Morfin	Morphine habit		
Misbrug af Tobak	Tobacco		
Nevrasteni			Neurasthenia
Operation	Removal of ovaries		
Overanstrængelse	Overwork		
Overanstrængelse	Mental strain		
Overanstrængelse	Overstudy		
Overgangsalder	Menopause		
Pengespekulationer	Business Trouble		
Pengespekulationer	Financial Trouble		
Pneumoni	Pneumonia		
Psykopati		Personality disorder	
Puerperium	Puerperal trouble		
Religiøse Indflydelser	Christian Science		
Religiøse Indflydelser	Spiritualism		
Religiøse Indflydelser	Religion		
Sindsindtryk		Schizoaffective disorder	Psychosis
Sindslidelser		Manic episode	Mania
Sindslidelser		Manic episode	

Table 6: Mapping of terms used in comparison with other locations *(continued)*

Norway	Oregon	Wales	Sweden
Sindslidelser		Bipolar disorder	Mania
Sindslidelser		Bipolar disorder	
Skarlagensfeber	Scarlatina		
Slagtilfælde	Cerebral embolism		
Socialt Udsat	Trouble		
Solstik	Sunstroke		
Sorg	Grief	Depressive episode	
Sorg	Grief	Depressive episode	Melancholia
Sorg	Melancholia	Depressive episode	
Sorg	Melancholia	Depressive episode	Melancholia
Søvnløshed	Loss of sleep		
Syphilis	Syphilis		
Typhoidfeber	Typhoid fever		
Ulykke	Injury		
Ulykke, Religiøse Indflydelser	Injury and religion		
Underernæring	Starvation		
UNKNOWN	Solitude		
UNKNOWN	Solitude		Others
UNKNOWN	Solitude	Delusional disorder	
UNKNOWN	Solitude	Delusional disorder	Others
UNKNOWN	Solitude	Psychological/behavioural disorder	
UNKNOWN	Solitude	Psychological/behavioural disorder	Others
UNKNOWN			Others
UNKNOWN		Delusional disorder	
UNKNOWN		Delusional disorder	Others
UNKNOWN		Psychological/behavioural disorder	
UNKNOWN		Psychological/behavioural disorder	Others
UNKNOWN		Other psychoses	
UNKNOWN		Other psychoses	Others
UNKNOWN	Unknown		
UNKNOWN	Unknown		Others
UNKNOWN	Unknown	Delusional disorder	
UNKNOWN	Unknown	Delusional disorder	Others
UNKNOWN	Unknown	Psychological/behavioural disorder	
UNKNOWN	Unknown	Psychological/behavioural disorder	Others
UNKNOWN	Unknown	Other psychoses	
UNKNOWN	Unknown	Other psychoses	Others
UNKNOWN	Suppressed eruption		
UNKNOWN	Suppressed eruption		Others
UNKNOWN	Suppressed eruption	Delusional disorder	
UNKNOWN	Suppressed eruption	Delusional disorder	Others
UNKNOWN	Suppressed eruption	Psychological/behavioural disorder	
UNKNOWN	Suppressed eruption	Psychological/behavioural disorder	Others
UNKNOWN	Suppressed eruption	Other psychoses	
UNKNOWN	Suppressed eruption	Other psychoses	Others
UNKNOWN	Solitude	Other psychoses	
UNKNOWN	Solitude	Other psychoses	Others
Uterinsygdom	Uterine disease		

References

Departementet for det Indre. Oversigt over Sindssygeasylernes Virksomhed i Aaret 1872 provided via Statistics Norway using ScanSoft OmniPage 16, 1873. URL https://www.ssb.no/historisk-statistikk/emner/helse-og-sosiale-forhold. PDF specification 1.6.

Departementet for det Indre. Oversigt over Sindssygeasylernes Virksomhed i Aaret 1873 provided via Statistics Norway using ScanSoft OmniPage 16, 1874. URL https://www.ssb.no/historisk-statistikk/emner/helse-og-sosiale-forhold. PDF specification 1.5.

Departementet for det Indre. Oversikt over Sindssygeasylernes Virksomhed i Aaret 1874 provided via Statistics Norway using ScanSoft OmniPage 16, 1875. URL https://www.ssb.no/historisk-statistikk/emner/helse-og-sosiale-forhold. PDF specification 1.6.

direktøren for det Civile Medicinalvæsen. Oversigt over Sindssygeasylernes Virksomhed i Aaret 1875 provided via Statistics Norway using ScanSoft OmniPage 16, 1876. URL https://www.ssb.no/historisk-statistikk/emner/helse-og-sosiale-forhold. PDF specification 1.5.

direktøren for det Civile Medicinalvæsen. Oversigt over Sindssygeasylernes Virksomhed i Aaret 1876 provided via Statistics Norway using ScanSoft OmniPage 16, 1877. URL https://www.ssb.no/historisk-statistikk/emner/helse-og-sosiale-forhold. PDF specification 1.6.

direktøren for det Civile Medicinalvæsen. Oversigt over Sindssygeasylernes Virksomhed i Aaret 1877 provided via Statistics Norway using ScanSoft OmniPage 16, 1878. URL https://www.ssb.no/historisk-statistikk/emner/helse-og-sosiale-forhold. PDF specification 1.6.

direktøren for det Civile Medicinalvæsen. Oversigt over Sindssygeasylernes Virksomhed i Aaret 1878 provided via Statistics Norway using ScanSoft OmniPage 16, 1879. URL https://www.ssb.no/historisk-statistikk/emner/helse-og-sosiale-forhold. PDF specification 1.5.

direktøren for det Civile Medicinalvæsen. Oversigt over Sindssygeasylernes Virksomhed i Aaret 1879 provided via Statistics Norway using ScanSoft OmniPage 16, 1880. URL https://www.ssb.no/historisk-statistikk/emner/helse-og-sosiale-forhold. PDF specification 1.6.

direktøren for det Civile Medicinalvæsen. Oversigt over Sinssygeasylernes Virksomhed i Aaret 1880 provided via Statistics Norway using ScanSoft OmniPage 16, 1883. URL https://www.ssb.no/historisk-statistikk/emner/helse-og-sosiale-forhold. PDF specification 1.5.

direktøren for det Civile Medicinalvæsen. Oversigt over Sindssygeasylernes Virksomhed i Aaret 1881 provided via Statistics Norway using ScanSoft OmniPage 16, 1884a. URL https://www.ssb.no/historisk-statistikk/emner/helse-og-sosiale-forhold. PDF specification 1.5.

direktøren for det Civile Medicinalvæsen. Oversigt over Sindssygeasylernes Virksomhed i Aaret 1882 provided via Statistics Norway using ScanSoft OmniPage 16, 1884b. URL https://www.ssb.no/historisk-statistikk/emner/helse-og-sosiale-forhold. PDF specification 1.6.

direktøren for det Civile Medicinalvæsen. Oversigt over Sindssygeasylernes Virksomhed i Aaret 1883 provided via Statistics Norway using ScanSoft OmniPage 16, 1884c. URL https://www.ssb.no/historisk-statistikk/emner/helse-og-sosiale-forhold. PDF specification 1.6.

direktøren for det Civile Medicinalvæsen. Oversigt over Sindssygeasylernes Virksomhed i Aaret 1884 provided via Statistics Norway using ScanSoft OmniPage 16, 1885. URL https://www.ssb.no/historisk-statistikk/emner/helse-og-sosiale-forhold. PDF specification 1.6.

direktøren for det Civile Medicinalvæsen. Oversigt over Sindssygeasylenes Virksomhed i Aaret 1885 provided via Statistics Norway using ScanSoft OmniPage 16, 1886. URL https://www.ssb.no/historisk-statistikk/emner/helse-og-sosiale-forhold. PDF specification 1.6.

direktøren for det Civile Medicinalvæsen. Oversigt over Sindssygeasylernes Virksomhed i Aaret 1886 provided via Statistics Norway using ScanSoft OmniPage 16, 1887. URL https://www.ssb.no/historisk-statistikk/emner/helse-og-sosiale-forhold. PDF specification 1.5.

direktøren for det Civile Medicinalvæsen. Oversigt over Sindssygeasylernes Virksomhed i Aaret 1887 provided via Statistics Norway using ScanSoft OmniPage 16, 1888. URL https://www.ssb.no/historisk-statistikk/emner/helse-og-sosiale-forhold. PDF specification 1.5.

direktøren for det Civile Medicinalvæsen. Oversigt over Sindssygeasylernes Virksomhed i Aaret 1888 provided via Statistics Norway using ScanSoft OmniPage 16, 1890. URL https://www.ssb.no/historisk-statistikk/emner/helse-og-sosiale-forhold. PDF specification 1.6.

Medicinalstyrelsen. Oversigt over Sindssygeasylernes Virksomhed i Aaret 1889 provided via Statistics Norway using ScanSoft OmniPage 16, 1890. URL https://www.ssb.no/historisk-statistikk/emner/helse-og-sosiale-forhold. PDF specification 1.5.

Medicinaldirektøren. Oversigt over Sindssygeasylernes Virksomhed i Aaret 1890 provided via Statistics Norway using ScanSoft OmniPage 16, 1891. URL https://www.ssb.no/historisk-statistikk/emner/helse-og-sosiale-forhold. PDF specification 1.5.

Medicinaldirektøren. Oversigt over Sindssygeasylernes Virksomhed i Aaret 1891 provided via Statistics Norway using ScanSoft OmniPage 16, 1894a. URL https://www.ssb.no/historisk-statistikk/emner/helse-og-sosiale-forhold. PDF specification 1.6.

Medicinaldirektøren. Oversigt over Sindssygeasylernes Virksomhed i Aaret 1892 provided via Statistics Norway using ScanSoft OmniPage 16, 1894b. URL https://www.ssb.no/historisk-statistikk/emner/helse-og-sosiale-forhold. PDF specification 1.5.

Medicinaldirektøren. Oversigt over Sindssygeasylernes Virksomhed i Aaret 1893 provided via Statistics Norway using ScanSoft OmniPage 16, 1895. URL https://www.ssb.no/historisk-statistikk/emner/helse-og-sosiale-forhold. PDF specification 1.6.

Medicinaldirektøren. Oversigt over Sindssygeasylernes Virksomhed i Aaret 1894 provided via Statistics Norway using ScanSoft OmniPage 16, 1896. URL https://www.ssb.no/historisk-statistikk/emner/helse-og-sosiale-forhold. PDF specification 1.6.

Medicinaldirektøren. Oversigt over Sindssygeasylernes Virksomhed i Aaret 1895 provided via Statistics Norway using ScanSoft OmniPage 16, 1897. URL https://www.ssb.no/historisk-statistikk/emner/helse-og-sosiale-forhold. PDF specification 1.6.

Medicinaldirektøren. Oversigt over Sindssygeasylernes Virksomhed i Aaret 1896 provided via Statistics Norway using ScanSoft OmniPage 16, 1898. URL https://www.ssb.no/historisk-statistikk/emner/helse-og-sosiale-forhold. PDF specification 1.6.

Medicinaldirektøren. Oversikt over Sindssygeasylernes Virksomhed i Aaret 1897 provided via Statistics Norway using ScanSoft OmniPage 16, 1899. URL https://www.ssb.no/historisk-statistikk/emner/helse-og-sosiale-forhold. PDF specification 1.6.

Medicinaldirektøren. Oversigt over Sindssygeasylernes Virksomhed i Aaret 1898 provided via Statistics Norway using ScanSoft OmniPage 16, 1900. URL https://www.ssb.no/historisk-statistikk/emner/helse-og-sosiale-forhold. PDF specification 1.5.

Medicinaldirektøren. Oversigt over Sindssygeasylernes Virksomhed i Aaret 1899 provided via Statistics Norway using ScanSoft OmniPage 16, 1901. URL https://www.ssb.no/historisk-statistikk/emner/helse-og-sosiale-forhold. PDF specification 1.5.

Medicinaldirektøren. Oversigt over Sindssygeasylernes Virksomhed i Aaret 1900 provided via Statistics Norway using ScanSoft OmniPage 16, 1902. URL https://www.ssb.no/historisk-statistikk/emner/helse-og-sosiale-forhold. PDF specification 1.6.

Medicinaldirektøren. Oversigt over Sindssygeasylernes Virksomhet i Aaret 1901 provided via Statistics Norway using ScanSoft OmniPage 16, 1903a. URL https://www.ssb.no/historisk-statistikk/emner/helse-og-sosiale-forhold. PDF specification 1.5.

Medicinaldirektøren. Oversigt over Sindsygeasylernes Virksomhet i Aaret 1902 provided via Statistics Norway using ScanSoft OmniPage 16, 1903b. URL https://www.ssb.no/historisk-statistikk/emner/helse-og-sosiale-forhold. PDF specification 1.6.

Medicinaldirektøren. Oversigt over Sindssygeasylernes Virksomhed Aaret 1903 provided via Statistics Norway using ScanSoft OmniPage 16, 1905. URL https://www.ssb.no/historisk-statistikk/emner/helse-og-sosiale-forhold. PDF specification 1.6.

Medicinaldirektøren. Sindssygeasylernes Virksomhed 1904 provided via Statistics Norway using ScanSoft OmniPage 16, 1906. URL https://www.ssb.no/historisk-statistikk/emner/helse-og-sosiale-forhold. PDF specification 1.6.

Medicinaldirektøren. Sindssygeasylernes Virksomhed, 1905 provided via Statistics Norway using ScanSoft OmniPage 16, 1907. URL https://www.ssb.no/historisk-statistikk/emner/helse-og-sosiale-forhold. PDF specification 1.6.

Medicinaldirektøren. Sindssykeasylernes Virksomhet, 1906 provided via Statistics Norway using ScanSoft OmniPage 16, 1908. URL https://www.ssb.no/historisk-statistikk/emner/helse-og-sosiale-forhold. PDF specification 1.5.

Medicinaldirektøren. Sindssykeasylernes Virksomhet, 1907 provided via Statistics Norway using ScanSoft OmniPage 16, 1909a. URL https://www.ssb.no/historisk-statistikk/emner/helse-og-sosiale-forhold. PDF specification 1.5.

Medicinaldirektøren. Sindssykeasylernes Virksomhet, 1908, Efter de fra asylerne indkomne aarsberetninger provided via Statistics Norway using ScanSoft OmniPage 16, 1909b. URL https://www.ssb.no/historisk-statistikk/emner/helse-og-sosiale-forhold. PDF specification 1.6.

Medicinaldirektøren. Sindssykeasylernes Virksomhet, 1909. Efter de fra asylerne indkomne aarsberetninger provided via Statistics Norway using ScanSoft OmniPage 16, 1911. URL https://www.ssb.no/historisk-statistikk/emner/helse-og-sosiale-forhold. PDF specification 1.6.

Medicinaldirektøren. Sindsykeaylenes Virksomhet, 1910, Etter de fra asylene indkomne aarsberetninger provided via Statistics Norway using ScanSoft OmniPage 16, 1912. URL https://www.ssb.no/historisk-statistikk/emner/helse-og-sosiale-forhold. PDF specification 1.6.

Medicinaldirektøren. Sindsykeasylenes Virksomhet 1911 provided via Statistics Norway using ScanSoft OmniPage 16, 1913. URL https://www.ssb.no/historisk-statistikk/emner/helse-og-sosiale-forhold. PDF specification 1.5.

Medicinaldirektøren. Sindssykeasylenes virksomhet 1912 provided via Statistics Norway using ScanSoft OmniPage 16, 1914. URL https://www.ssb.no/historisk-statistikk/emner/helse-og-sosiale-forhold. PDF specification 1.5.

Medicinaldirektøren. Sindsykeasylenes virksomhet 1913 provided via Statistics Norway using ScanSoft OmniPage 16, 1915. URL https://www.ssb.no/historisk-statistikk/emner/helse-og-sosiale-forhold. PDF specification 1.6.

Medicinaldirektøren. Sindssykeasylenes virksomhet, 1914 provided via Statistics Norway using ScanSoft OmniPage 16, 1916. URL https://www.ssb.no/historisk-statistikk/emner/helse-og-sosiale-forhold. PDF specification 1.6.

Medicinaldirektøren. Sindssykeasylenes virksomhet, 1915, Efter de fra asylene indkomne aarsberetninger provided via Statistics Norway using ScanSoft OmniPage 16, 1917. URL https://www.ssb.no/historisk-statistikk/emner/helse-og-sosiale-forhold. PDF specification 1.5.

Medisinaldirektøren. Sinnssykeasylenes virksomhet, 1916, Efter de fra asylene innkomne årsberetninger provided via Statistics Norway using ScanSoft OmniPage 16, 1920a. URL https://www.ssb.no/historisk-statistikk/emner/helse-og-sosiale-forhold. PDF specification 1.5.

Medisinaldirektøren. Sinnssykeasylenes virksomhet 1917 provided via Statistics Norway using ScanSoft Omnipage 17, 1920b. URL https://www.ssb.no/historisk-statistikk/emner/helse-og-sosiale-forhold. PDF specification 1.6.

Medisinaldirektøren. Sinnssykeasylenes virksomhet 1918 provided via Statistics Norway using ScanSoft OmniPage 16, 1921. URL https://www.ssb.no/historisk-statistikk/emner/helse-og-sosiale-forhold. PDF specification 1.5.

Medisinaldirektøren. Sinnssykeasylenes virksomhet 1919 provided via Statistics Norway using ScanSoft OmniPage 16, 1923a. URL https://www.ssb.no/historisk-statistikk/emner/helse-og-sosiale-forhold. PDF specification 1.5.

Medisinaldirektøren. Sinnssykeasylenes virksomhet 1920 provided via Statistics Norway using ScanSoft OmniPage 16, 1923b. URL https://www.ssb.no/historisk-statistikk/emner/helse-og-sosiale-forhold. PDF specification 1.5.

Medisinaldirektøren. Sinnssykeasylenes virksomhet 1921 provided via Statistics Norway using ScanSoft OmniPage 16, 1923c. URL https://www.ssb.no/historisk-statistikk/emner/helse-og-sosiale-forhold. PDF specification 1.5.

Medisinaldirektøren. Sinnssykeasylenes virksomhet 1922 provided via Statistics Norway using ScanSoft OmniPage 16, 1925. URL https://www.ssb.no/historisk-statistikk/emner/helse-og-sosiale-forhold. PDF specification 1.6.

Overlægen for Sinnssykevesenet. Sinnssykeasylenes virksomhet 1923 provided via Statistics Norway using ScanSoft OmniPage 16, 1925a. URL https://www.ssb.no/historisk-statistikk/emner/helse-og-sosiale-forhold. PDF specification 1.5.

Overlægen for Sinnssykevesenet. Sinnssykeasylenes virksomhet 1924 provided via Statistics Norway using ScanSoft OmniPage 16, 1925b. URL https://www.ssb.no/historisk-statistikk/emner/helse-og-sosiale-forhold. PDF specification 1.5.

Overlægen for Sinnssykevesenet. Sinnssykeasylenes virksomhet 1925 provided via Statistics Norway using ScanSoft OmniPage 16, 1927. URL https://www.ssb.no/historisk-statistikk/emner/helse-og-sosiale-forhold. PDF specification 1.5.

Medisinaldirektøren. Sinnssykeasylenes virksomhet 1926 og opgave over antall offentlig forpleide sinnssyke i 1925 provided via Statistics Norway using ScanSoft OmniPage 16, 1928. URL https://www.ssb.no/historisk-statistikk/emner/helse-og-sosiale-forhold. PDF specification 1.5.

Medisinaldirektøren. Sinnssykeasylenes virksomhet 1927 og opgave over antall offentlig forpleide sinnssyke i 1926 provided via Statistics Norway using ScanSoft OmniPage 16, 1929. URL https://www.ssb.no/historisk-statistikk/emner/helse-og-sosiale-forhold. PDF specification 1.6.

Medisinaldirektøren. Sinnssykeasylenes virksomhet 1928 og opgave over antall offentlig forpleide sinnssyke i 1927 provided via Statistics Norway using ScanSoft OmniPage 16, 1930. URL https://www.ssb.no/historisk-statistikk/emner/helse-og-sosiale-forhold. PDF specification 1.6.

Medisinaldirektøren. Sinnssykeasylenes virksomhet 1929 og opgave over antall offentlig forpleide sinnssyke i 1928 provided via Statistics Norway using ScanSoft OmniPage 16, 1931. URL https://www.ssb.no/historisk-statistikk/emner/helse-og-sosiale-forhold. PDF specification 1.5.

A. R. Omran. The epidemiologic transition: A theory of the epidemiology of population change. *Milbank Quarterly*, 83(4):731–757, 2005. DOI 10.1111/j.1468-0009.2005.00398.x.

G. Thorvaldsen and L. Holden. The development of microhistorical databases in Norway. a historiography. *Historical Life Course Studies*, 13:127–47, 2023. DOI 10.51964/hlcs14315.
